# Exercise type, dose and mental health outcomes in youth: Which types and doses are sufficient?

**DOI:** 10.1002/gps3.70031

**Published:** 2026-06-12

**Authors:** Huagen Wang, Yisheng Aku, Shicun Xu, Zhanbing Ren, Yingjie Zhang, Runsen Chen, Jing An

**Affiliations:** ^1^ Department of Psychology, School of Humanities and Social Sciences Beijing Forestry University Beijing China; ^2^ Vanke School of Public Health Tsinghua University Beijing China; ^3^ College of Athletic Training Chengdu Sports University Chengdu Sichuan China; ^4^ Northeast Asian Research Center Jilin University Changchun Jilin China; ^5^ Department of Population, Resources and Environment, Northeast Asian Studies College Jilin University Changchun Jilin China; ^6^ College of Physical Education Shenzhen University Shenzhen Guangdong China; ^7^ Tsinghua University Hospital Beijing China; ^8^ Institute for Healthy China Tsinghua University Beijing China; ^9^ Beijing Huilongguan Hospital, Capital Medical University Beijing China; ^10^ Huilongguan Clinical Medical School Peking University Beijing China; ^11^ WHO Collaborating Center for Research and Training in Suicide Prevention Beijing China

## Abstract

**Background:**

Although experimental evidence supports links between exercise and certain mental health indicators, epidemiological research has focused primarily on the dose–response effect of a single type of exercise, overlooking potential differences across exercise types in their associations with overall and specific mental health outcomes.

**Aims:**

To examine the associations between different types of exercise and multiple mental health outcomes, including depression, anxiety, post‐traumatic stress disorder, suicidal ideation, suicide attempts and nonsuicidal self‐injury, and to explore their dose–response relationships in terms of exercise frequency and duration.

**Methods:**

Data were drawn from a large‐scale, cross‐sectional study of 79 011 university students in Jilin Province, China, in 2021 via an online survey. Mental health outcomes were evaluated using validated scales and diagnoses. Propensity score matching was first applied to balance covariates between exercise types. Logistic regression analysis was then used to examine the associations between exercise types and mental health outcomes. Additionally, generalised additive models were employed to explore the dose–response relationships between exercise frequency/duration and mental health outcomes, adjusting for sociodemographic factors.

**Results:**

Among the 79 011 students reporting participation in physical exercise, team ball sports showed the strongest protective associations, followed by single anaerobic activities, two‐player racket sports, single moderate‐intensity aerobic sports and single low‐intensity aerobic sports. U‐shaped associations were observed between exercise frequency, duration and mental health outcomes. Exercising three to four times per week for 90–120 min was associated with a lower probability of reporting mental health problems, whereas excessive exercise was associated with poorer outcomes.

**Conclusion:**

Participation in different types of exercise is differentially associated with mental health outcomes, with team ball sports showing the most favourable associations. Optimal levels of exercise dosage vary between exercise types, suggesting that individuals may benefit from selecting exercise patterns that best support their mental health.

## Abstract (highlights)

**WHAT IS ALREADY KNOWN ON THIS TOPIC**

- Although experimental evidence supports links between exercise and certain mental health indicators, epidemiological research has focused primarily on the dose–response effects of physical activity in general, neglecting the varied implications of different exercise types on overall and specific mental health outcomes, such as post‐traumatic stress disorder, suicidal ideation, suicide attempts and nonsuicidal self‐injury.

**WHAT THIS STUDY ADDS**

- Drawing on a large‐scale epidemiological sample of 79 011 university students who engaged in physical exercise, this study demonstrates that the protective effects of physical activity vary by exercise type, with team ball sports conferring the greatest mental health benefits, followed by single anaerobic activities, two‐player racket sports, single moderate‐intensity aerobic activities and single low‐intensity aerobic activities. Furthermore, it reveals nonlinear dose–response relationships, identifying an optimal threshold of three to four times per week for 90–120 min.

**HOW THIS STUDY MIGHT AFFECT RESEARCH, PRACTICE OR POLICY**

- These findings suggest that public health guidelines and clinical practice should move towards tailored exercise prescriptions that consider specific modalities and optimal dosages. Interventions for youth mental health should prioritise exercise types based on individual needs and carefully monitor exercise volume to maximise benefits while mitigating potential adverse effects of over‐exercising.

## INTRODUCTION

The psychological well‐being of adolescents and young adults has become a paramount global concern. Recent systematic analyses from the Global Burden of Disease Study 2021 highlight the significant and dynamic burden of mental disorders among individuals aged 10–24 years, emphasising the urgent need for prioritised prevention in the postpandemic era.^1^ The complexity of this issue is further underscored by the intricate network of associations between depressive symptoms, anxiety and suicidal ideation.^2^ Among these psychological challenges, nonsuicidal self‐injury (NSSI) and suicidal behaviours are particularly critical. Understanding the mechanisms underlying NSSI, especially its co‐occurrence with major depressive disorder, is essential for developing effective prevention strategies.^3^

Participation in physical activity and sports has been associated with better mental health and well‐being.^4^, ^5^, ^6^ Prospective and meta‐analytic studies report a reduced risk of depression and lower levels of anxiety symptoms among physically active individuals.^7^, ^8^, ^9^ Randomised and time‐targeted exercise interventions show promising adjunctive effects for post‐traumatic stress disorder (PTSD) and depressive symptoms across age groups. Observational studies also report inverse associations between physical activity and psychosis/schizophrenia as well as suicidal behaviours.^10^, ^11^, ^12^, ^13^ Several neurobiological and behavioural mechanisms have been proposed to explain the transdiagnostic links between physical activity and mental health outcomes, including improved neurobiological functioning, enhanced neuroplasticity and strengthened cognitive control and emotion regulation.^6^, ^14^, ^15^, ^16^

Despite this evidence, existing literature often treats exercise as a homogeneous construct. However, different exercise modalities may not provide equivalent psychological benefits. Compared with individual activities, participation in group‐based activities such as running groups or team sports has been associated with better mental health outcomes and greater social connectedness.^17^ These differences may reflect two primary, and likely synergistic, pathways through which sport influences mental health: physiological mechanisms and social relationships. From a physiological perspective, exercise may influence mental health through neurobiological pathways, including effects on brain‐derived neurotrophic factor and executive function.^15^, ^16^ Different exercise modalities modulate distinct neural pathways and brain regions.^6^, ^18^ For example, aerobic exercise enhances functional connectivity in the primary motor cortex, whereas anaerobic exercise more strongly engages the prefrontal lobe,^19^ a region central to cognition and emotional regulation that is frequently implicated in mental health conditions.^20^ From a social perspective, the ‘mental health through sport’ conceptual model explicitly recognises that the physical, mental and social benefits of sport participation are context‐dependent (e.g., individual vs. team and organised vs. informal).^5^ Sport participation provides a unique opportunity to form social relationships and develop supportive networks. Accordingly, distinguishing activities not only by physiological characteristics such as intensity but also by their social context is important. A meta‐analysis showed that aerobic and resistance training as exercise types showed large effects, whereas mixed aerobic and resistance training showed small effects.^21^ Engagement in social exercise (e.g., group walking or group golf) has been linked to better self‐rated health and lower depressive symptoms compared with similar activities performed individually.^22^ Team sports, in particular, enhance psychological resilience and self‐worth through unique mechanisms such as fostering a sense of belonging and companionship, providing social support and offering behavioural guidance and a clear sense of purpose through role‐based identity within a team.^6^

Although the evidence highlights the importance of context, intensity and social characteristics in shaping the mental health benefits of exercise, few studies have systematically compared a comprehensive range of sport modalities across multiple mental health outcomes. To address this gap, this study examines the associations between five categories of physical activity and multiple mental health outcomes in a large cohort. The activity categories comprised team ball sports, single anaerobic activities, two‐player racket sports, single moderate‐intensity aerobic sports and single low‐intensity aerobic sports; mental health outcomes included depression, anxiety, PTSD, NSSI, suicidal ideation and suicide attempts. These categories were structured based on a synergistic framework combining two critical axes: social complexity (individual, paired and team activities) and physiological demand (low‐intensity aerobic, moderate‐intensity aerobic and anaerobic activities). Our primary aims are to (1) compare the relative associations of these five categories of physical activity with each mental health outcome, (2) test whether activities with higher social engagement (e.g., team ball sports) show stronger associations with mental health outcomes than low‐social/low‐intensity activities and (3) evaluate the effect size and clinical relevance of these associations to inform evidence‐based public health and exercise recommendations.

Furthermore, we hypothesise that different types of exercise modalities exhibit distinct dose–response relationships for specific mental health outcomes. Although meta‐analyses confirm an inverse curvilinear association between general physical activity and outcomes such as depression, these findings are based on aggregating heterogeneous activities and likely mask important modality‐specific differences.^21^, ^23^ The ‘cost’ (time and energy) and ‘gain’ (psychological and social outcomes) curves likely shift depending on the unique attributes of each activity type.^5^ Given that different sport modalities trigger distinct neurobiological and social pathways, it is biologically plausible that the optimal dose required to activate these pathways differs by exercise type. For instance, team ball sports may reach maximal psychological benefit with a shorter duration compared to solitary aerobic activities, as their greater social engagement may provide additional psychological benefit. By testing this specific hypothesis across five exercise categories, this study aims to provide more precise evidence to inform tailored and optimised exercise recommendations for mental health.

## METHODS

### Data source and participants

This study used data from a large‐scale, cross‐sectional study conducted between 26 October and 18 November 2021 among university and college students in Jilin Province, China. The online questionnaire was distributed via a Quick Response code to all 63 universities and colleges in the province. All the participants provided informed consent online before completing the questionnaire. Participants were informed of their right to withdraw from the survey at any time. All participants received information regarding how to access mental health support if they experienced any emotional distress during or after the study. Participants deemed to be at high risk for suicide were provided with information on suicide prevention hotlines and advised to seek professional help. Help‐seeking information from local hospitals' mental health departments was also available. Participants received small cash incentives for completing the survey. See Supporting Information S2 for the Checklist for Reporting Results of Internet E‐Surveys. Patients and the public were not involved in the design, conduct, reporting or dissemination of this research.

**FIGURE 1:**
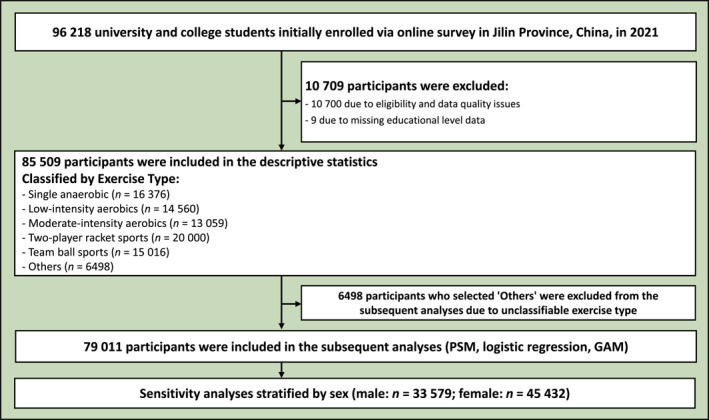
Study flowchart. BMI, body mass index; GAM, generalised additive model; PSM, propensity score matching.

The study initially involved 96 218 participants. Participants were excluded from the initial sample based on the following eligibility and data quality criteria: (1) being under 17 years old; (2) inability to understand the contents of the questionnaire; (3) answering fewer than three out of the four attention check questions correctly; (4) reporting implausible height and weight values; (5) providing logically contradictory responses or having missing responses; (6) selecting responses unrelated to the study objectives (e.g., addictive behaviours); and (7) showing straightlining behaviour (e.g., consistently selecting the first response option). After this initial quality screening, we further excluded individuals who had missing data on key variables (height, weight and education level), yielding an analytic sample of 85 509 participants (table 1). For the main regression analyses, participants reporting exercise types outside our predefined categories (i.e., ‘exercises not otherwise classified’; *n* = 6498) were additionally excluded, resulting in a final cohort of 79 011 participants (tables 2 and 3). The number of participants at each stage is presented in figure 1.

**TABLE 1:** Participant characteristics stratified by total exercise types

	Single anaerobic (*n* = 16 376)	Low‐intensity aerobics (*n* = 14 560)	Moderate‐intensity aerobics (*n* = 13 059)	Two‐player racket sport (*n* = 20 000)	Team ball (*n* = 15 016)	Others (*n* = 6498)	Overall (*n* = 85 509)
Age							
≤ 18	4584 (28.0%)	4002 (27.5%)	3554 (27.2%)	5514 (27.6%)	4053 (27.0%)	1747 (26.9%)	23 454 (27.4%)
19–21	9917 (60.6%)	9118 (62.6%)	8187 (62.7%)	12 386 (61.9%)	9126 (60.8%)	4039 (62.2%)	52 773 (61.7%)
22–24	1578 (9.6%)	1236 (8.5%)	1118 (8.6%)	1791 (9.0%)	1529 (10.2%)	586 (9.0%)	7838 (9.2%)
> 24	297 (1.8%)	204 (1.4%)	200 (1.5%)	309 (1.5%)	308 (2.1%)	126 (1.9%)	1444 (1.7%)
Gender							
Male	8778 (53.6%)	2621 (18.0%)	4312 (33.0%)	7428 (37.1%)	10 440 (69.5%)	1937 (29.8%)	35 516 (41.5%)
Female	7598 (46.4%)	11 939 (82.0%)	8747 (67.0%)	12 572 (62.9%)	4576 (30.5%)	4561 (70.2%)	49 993 (58.5%)
BMI							
Mean (SD)	22.7 (5.22)	22.7 (5.21)	22.7 (5.22)	22.7 (5.26)	22.6 (5.26)	22.7 (5.19)	22.7 (5.23)
Gender identification							
Cisgender	14 749 (90.1%)	13 336 (91.6%)	11 847 (90.7%)	18 131 (90.7%)	13 325 (88.7%)	5674 (87.3%)	77 062 (90.1%)
Transgender	232 (1.4%)	204 (1.4%)	217 (1.7%)	292 (1.5%)	219 (1.5%)	109 (1.7%)	1273 (1.5%)
Nonbinary/genderqueer	222 (1.4%)	353 (2.4%)	317 (2.4%)	374 (1.9%)	185 (1.2%)	144 (2.2%)	1595 (1.9%)
Uncertainty	271 (1.7%)	188 (1.3%)	174 (1.3%)	291 (1.5%)	245 (1.6%)	133 (2.0%)	1302 (1.5%)
Questioning	902 (5.5%)	479 (3.3%)	504 (3.9%)	912 (4.6%)	1042 (6.9%)	438 (6.7%)	4277 (5.0%)
Ethnicity							
Others	1544 (9.4%)	1479 (10.2%)	1347 (10.3%)	1948 (9.7%)	1833 (12.2%)	834 (12.8%)	8985 (10.5%)
Han	14 832 (90.6%)	13 081 (89.8%)	11 712 (89.7%)	18 052 (90.3%)	13 183 (87.8%)	5664 (87.2%)	76 524 (89.5%)
Only child							
No	8719 (53.2%)	7708 (52.9%)	6655 (51.0%)	10 907 (54.5%)	7354 (49.0%)	3609 (55.5%)	44 952 (52.6%)
Yes	7657 (46.8%)	6852 (47.1%)	6404 (49.0%)	9093 (45.5%)	7662 (51.0%)	2889 (44.5%)	40 557 (47.4%)
Father's education level							
No education	200 (1.2%)	119 (0.8%)	108 (0.8%)	194 (1.0%)	163 (1.1%)	94 (1.4%)	878 (1.0%)
Primary school	3116 (19.0%)	2540 (17.4%)	1905 (14.6%)	3715 (18.6%)	2589 (17.2%)	1390 (21.4%)	15 255 (17.8%)
Junior high school	6368 (38.9%)	5312 (36.5%)	4481 (34.3%)	7727 (38.6%)	5671 (37.8%)	2426 (37.3%)	31 985 (37.4%)
High school/technical school	3720 (22.7%)	3376 (23.2%)	3142 (24.1%)	4447 (22.2%)	3409 (22.7%)	1279 (19.7%)	19 373 (22.7%)
Associate degree	1172 (7.2%)	1279 (8.8%)	1360 (10.4%)	1611 (8.1%)	1258 (8.4%)	463 (7.1%)	7143 (8.4%)
Bachelor's degree	997 (6.1%)	1185 (8.1%)	1377 (10.5%)	1371 (6.9%)	1116 (7.4%)	387 (6.0%)	6433 (7.5%)
Master's/PhD/postdoctoral	133 (0.8%)	132 (0.9%)	182 (1.4%)	167 (0.8%)	159 (1.1%)	40 (0.6%)	813 (1.0%)
Not sure	341 (2.1%)	303 (2.1%)	226 (1.7%)	379 (1.9%)	350 (2.3%)	214 (3.3%)	1813 (2.1%)
Others	329 (2.0%)	314 (2.2%)	278 (2.1%)	389 (1.9%)	301 (2.0%)	205 (3.2%)	1816 (2.1%)
Mother's education level							
No education	433 (2.6%)	301 (2.1%)	263 (2.0%)	480 (2.4%)	401 (2.7%)	240 (3.7%)	2118 (2.5%)
Primary school	3756 (22.9%)	3027 (20.8%)	2373 (18.2%)	4444 (22.2%)	3026 (20.2%)	1576 (24.3%)	18 202 (21.3%)
Junior high school	6047 (36.9%)	5154 (35.4%)	4294 (32.9%)	7435 (37.2%)	5433 (36.2%)	2299 (35.4%)	30 662 (35.9%)
High school/technical school	3545 (21.6%)	3351 (23.0%)	3096 (23.7%)	4228 (21.1%)	3357 (22.4%)	1273 (19.6%)	18 850 (22.0%)
Associate degree	1083 (6.6%)	1177 (8.1%)	1315 (10.1%)	1497 (7.5%)	1181 (7.9%)	443 (6.8%)	6696 (7.8%)
Bachelor's degree	867 (5.3%)	982 (6.7%)	1221 (9.3%)	1214 (6.1%)	1004 (6.7%)	334 (5.1%)	5622 (6.6%)
Master's/PhD/postdoctoral	95 (0.6%)	92 (0.6%)	130 (1.0%)	110 (0.6%)	111 (0.7%)	29 (0.4%)	567 (0.7%)
Not sure	353 (2.2%)	310 (2.1%)	229 (1.8%)	392 (2.0%)	346 (2.3%)	219 (3.4%)	1849 (2.2%)
Others	197 (1.2%)	166 (1.1%)	138 (1.1%)	200 (1.0%)	157 (1.0%)	85 (1.3%)	943 (1.1%)
Household income							
< ¥6000	5248 (32.0%)	4243 (29.1%)	3352 (25.7%)	5942 (29.7%)	4350 (29.0%)	2228 (34.3%)	25 363 (29.7%)
¥6000–¥14 000	5333 (32.6%)	4777 (32.8%)	4138 (31.7%)	6777 (33.9%)	4669 (31.1%)	2062 (31.7%)	27 756 (32.5%)
¥14 000–¥23 000	2582 (15.8%)	2541 (17.5%)	2290 (17.5%)	3356 (16.8%)	2536 (16.9%)	993 (15.3%)	14 298 (16.7%)
¥23 000–¥36 000	1455 (8.9%)	1473 (10.1%)	1475 (11.3%)	1946 (9.7%)	1505 (10.0%)	519 (8.0%)	8373 (9.8%)
¥36 000–¥70 000	1020 (6.2%)	983 (6.8%)	1055 (8.1%)	1232 (6.2%)	1098 (7.3%)	396 (6.1%)	5784 (6.8%)
> ¥70 000	738 (4.5%)	543 (3.7%)	749 (5.7%)	747 (3.7%)	858 (5.7%)	300 (4.6%)	3935 (4.6%)
Drinking							
Never	6348 (38.8%)	7395 (50.8%)	5577 (42.7%)	8476 (42.4%)	3985 (26.5%)	2773 (42.7%)	34 554 (40.4%)
≤ 1 time/month	7591 (46.4%)	5877 (40.4%)	5742 (44.0%)	9165 (45.8%)	7622 (50.8%)	2771 (42.6%)	38 768 (45.3%)
2–4 times/month	1912 (11.7%)	1027 (7.1%)	1347 (10.3%)	1906 (9.5%)	2734 (18.2%)	699 (10.8%)	9625 (11.3%)
2–3 times/week	322 (2.0%)	176 (1.2%)	279 (2.1%)	291 (1.5%)	444 (3.0%)	155 (2.4%)	1667 (1.9%)
≥ 4 times/week	203 (1.2%)	85 (0.6%)	114 (0.9%)	162 (0.8%)	231 (1.5%)	100 (1.5%)	895 (1.0%)
Smoking							
No	13 814 (84.4%)	13 777 (94.6%)	11 712 (89.7%)	18 104 (90.5%)	11 226 (74.8%)	5582 (85.9%)	74 215 (86.8%)
Yes	2562 (15.6%)	783 (5.4%)	1347 (10.3%)	1896 (9.5%)	3790 (25.2%)	916 (14.1%)	11 294 (13.2%)

*Note*: ‘Others’ in father's or mother's education level indicates that the father's or mother's role was absent, for example, the mother or father has passed away etc.

Abbreviations: BMI, body mass index; SD, standard deviation.

**TABLE 2:** Logistic regression results of exercise types on various mental health outcomes

Predictors	NSSI			SI			SA			Depression			Anxiety			PTSD		
	Odds ratios	95% CI	*p*‐value (FDR)	Odds ratios	95% CI	*p*‐value (FDR)	Odds ratios	95% CI	*p*‐value (FDR)	Odds ratios	95% CI	*p*‐value (FDR)	Odds ratios	95% CI	*p*‐value (FDR)	Odds ratios	95% CI	*p*‐value (FDR)
Reference: team ball																		
Two‐player racket sport	1.05	0.96–1.15	0.491	1.24	1.18–1.32	**< 0.001**	1.08	0.92–1.28	0.428	1.17	1.08–1.26	**< 0.001**	1.12	1.01–1.25	0.067	1.11	1.05–1.18	**0.002**
Low‐intensity aerobics	1.40	1.27–1.55	**< 0.001**	1.62	1.52–1.72	**< 0.001**	1.47	1.23–1.75	**< 0.001**	1.63	1.50–1.78	**< 0.001**	1.64	1.47–1.84	**< 0.001**	1.29	1.20–1.38	**< 0.001**
Moderate‐intensity aerobics	1.25	1.14–1.38	**< 0.001**	1.45	1.37–1.54	**< 0.001**	1.35	1.13–1.61	**0.002**	1.31	1.20–1.42	**< 0.001**	1.38	1.24–1.54	**< 0.001**	1.19	1.12–1.28	**< 0.001**
Single anaerobics	1.14	1.04–1.25	**0.016**	1.15	1.09–1.22	**< 0.001**	1.18	0.99–1.41	0.104	1.17	1.08–1.27	**< 0.001**	1.24	1.11–1.38	**< 0.001**	1.12	1.05–1.19	**0.002**
Reference: two‐player racket sport																		
Low‐intensity aerobics	1.34	1.23–1.45	**< 0.001**	1.30	1.24–1.37	**< 0.001**	1.35	1.16–1.57	**< 0.001**	1.40	1.30–1.50	**< 0.001**	1.46	1.33–1.61	**< 0.001**	1.16	1.09–1.23	**< 0.001**
Moderate‐intensity aerobics	1.19	1.10–1.30	**< 0.001**	1.17	1.11–1.23	**< 0.001**	1.24	1.08–1.44	**0.009**	1.12	1.04–1.20	**0.004**	1.23	1.12–1.35	**< 0.001**	1.07	1.01–1.14	**0.028**
Single anaerobics	1.09	1.01–1.18	0.078	0.93	0.88–0.97	**0.005**	1.09	0.94–1.26	0.366	1.00	0.93–1.07	0.966	1.10	1.01–1.20	0.078	1.00	0.95–1.06	0.938
Team ball	0.95	0.87–1.05	0.491	0.80	0.76–0.85	**< 0.001**	0.92	0.78–1.09	0.428	0.86	0.79–0.93	**< 0.001**	0.89	0.80–0.99	0.071	0.90	0.85–0.96	**0.002**
Reference: low‐intensity aerobics																		
Two‐player racket sport	0.75	0.69–0.81	**< 0.001**	0.77	0.73–0.81	**< 0.001**	0.74	0.64–0.86	**< 0.001**	0.72	0.66–0.77	**< 0.001**	0.68	0.62–0.75	**< 0.001**	0.86	0.81–0.92	**< 0.001**
Moderate‐intensity aerobics	0.89	0.82–0.98	**0.032**	0.90	0.85–0.95	**< 0.001**	0.92	0.79–1.08	0.385	0.80	0.74–0.87	**< 0.001**	0.84	0.76–0.93	**0.002**	0.93	0.87–0.99	**0.040**
Single anaerobics	0.81	0.75–0.89	**< 0.001**	0.71	0.67–0.75	**< 0.001**	0.81	0.69–0.94	**0.017**	0.72	0.66–0.77	**< 0.001**	0.75	0.68–0.83	**< 0.001**	0.87	0.81–0.92	**< 0.001**
Team ball	0.71	0.65–0.79	**< 0.001**	0.62	0.58–0.66	**< 0.001**	0.68	0.57–0.82	**< 0.001**	0.61	0.56–0.67	**< 0.001**	0.61	0.54–0.68	**< 0.001**	0.78	0.72–0.83	**< 0.001**
Reference: moderate‐intensity aerobics																		
Two‐player racket sport	0.84	0.77–0.91	**< 0.001**	0.86	0.81–0.90	**< 0.001**	0.80	0.70–0.93	**0.009**	0.89	0.83–0.96	**0.004**	0.81	0.74–0.89	**< 0.001**	0.93	0.88–0.99	**0.030**
Low‐intensity aerobics	1.12	1.02–1.22	**0.034**	1.12	1.05–1.18	**< 0.001**	1.09	0.93–1.27	0.385	1.25	1.15–1.35	**< 0.001**	1.19	1.08–1.31	**0.002**	1.08	1.01–1.15	**0.042**
Single anaerobics	0.91	0.84–0.99	0.073	0.79	0.75–0.84	**< 0.001**	0.88	0.75–1.02	0.157	0.89	0.83–0.97	**0.007**	0.89	0.81–0.98	**0.048**	0.93	0.88–0.99	**0.049**
Team ball	0.80	0.73–0.88	**< 0.001**	0.69	0.65–0.73	**< 0.001**	0.74	0.62–0.88	**0.002**	0.77	0.70–0.83	**< 0.001**	0.72	0.65–0.81	**< 0.001**	0.84	0.78–0.90	**< 0.001**
Reference: single anaerobic																		
Two‐player racket sport	0.92	0.85–0.99	0.074	1.08	1.03–1.13	**0.005**	0.92	0.79–1.06	0.354	1.00	0.93–1.07	0.966	0.91	0.83–0.99	0.078	1.00	0.94–1.05	0.938
Low‐intensity aerobics	1.23	1.12–1.34	**< 0.001**	1.41	1.33–1.49	**< 0.001**	1.24	1.06–1.45	**0.019**	1.40	1.29–1.51	**< 0.001**	1.33	1.21–1.47	**< 0.001**	1.15	1.08–1.23	**< 0.001**
Moderate‐intensity aerobics	1.10	1.01–1.20	0.074	1.26	1.20–1.33	**< 0.001**	1.14	0.98–1.33	0.157	1.12	1.04–1.21	**0.007**	1.12	1.02–1.23	0.051	1.07	1.01–1.14	0.051
Team ball	0.88	0.80–0.96	**0.017**	0.87	0.82–0.92	**< 0.001**	0.85	0.71–1.01	0.108	0.86	0.79–0.93	**< 0.001**	0.81	0.73–0.90	**< 0.001**	0.90	0.84–0.96	**0.002**

*Note*: Propensity scores for the five exercise types were included as weights, and several covariates were included in the model. These covariates included age, sex assigned at birth, gender identity, ethnicity, BMI, only‐child status, education level, smoking status and alcohol consumption, family structure, household income and parental education level. Detailed information on these analyses can be found in tables S3–S7. The bold values indicate statistical significance (FDR‐adjusted *p* < 0.05). To account for multiple comparisons, *p*‐values were adjusted using the Benjamini‐Hochberg procedure to control the False Discovery Rate (FDR).

Abbreviations: BMI, body mass index; CI, confidence interval; FDR, false discovery rate; NSSI, nonsuicidal self‐injury; PTSD, post‐traumatic stress disorder; SA, suicide attempt; SI, suicidal ideation.

**TABLE 3:** Smooth terms influencing exercise dose as determined by logistic generalised additive modelling (*n* = 79 011)^a^

Predictors	NSSI			SI			SA			Depression			Anxiety			PTSD		
	Edf	Chi.sq	*p*‐value	Edf	Chi.sq	*p*‐value	Edf	Chi.sq	*p*‐value	Edf	Chi.sq	*p*‐value	Edf	Chi.sq	*p*‐value	Edf	Chi.sq	*p*‐value
Frequency																		
Two‐player racket sport	2.234	28.323	**<** **0.001**	2.546	116.537	**<** **0.001**	2.042	7.128	**0.035**	2.637	167.854	**<** **0.001**	2.391	77.099	**<** **0.001**	2.255	72.580	**<** **0.001**
Low‐intensity aerobics	1.344	2.238	0.357	2.149	32.612	**<** **0.001**	1.006	2.843	0.094	2.677	100.255	**<** **0.001**	2.300	36.410	**<** **0.001**	2.479	70.270	**<** **0.001**
Moderate‐intensity aerobics	1.307	0.135	0.869	2.891	43.829	**<** **0.001**	2.088	6.584	0.089	2.889	64.073	**<** **0.001**	2.741	46.932	**<** **0.001**	2.237	23.217	**<** **0.001**
Single anaerobics	1.799	6.451	**0.047**	2.845	78.546	**<** **0.001**	1.819	3.582	0.167	2.481	102.584	**<** **0.001**	2.030	45.793	**<** **0.001**	2.581	38.942	**<** **0.001**
Team ball	2.179	60.156	**<** **0.001**	2.591	201.798	**<** **0.001**	1.005	14.715	**<** **0.001**	2.742	206.662	**<** **0.001**	2.822	132.343	**<** **0.001**	2.539	104.158	**<** **0.001**
Duration																		
Two‐player racket sport	1.007	6.062	**0.014**	2.857	30.839	**<** **0.001**	2.446	10.103	**0.036**	2.551	98.519	**<** **0.001**	2.391	68.594	**<** **0.001**	2.088	58.819	**<** **0.001**
Low‐intensity aerobics	1.094	12.784	**0.001**	1.075	101.014	**<** **0.001**	2.064	8.515	**0.034**	2.249	127.396	**<** **0.001**	2.499	61.490	**<** **0.001**	2.106	40.851	**<** **0.001**
Moderate‐intensity aerobics	2.273	27.838	**<** **0.001**	1.888	131.534	**<** **0.001**	1.829	11.460	**0.005**	2.421	76.130	**<** **0.001**	2.222	30.915	**<** **0.001**	1.002	29.716	**<** **0.001**
Single anaerobics	1.805	29.626	**<** **0.001**	2.579	134.860	**<** **0.001**	2.739	10.912	**0.022**	2.675	107.003	**<** **0.001**	2.694	52.310	**<** **0.001**	1.881	55.072	**<** **0.001**
Team ball	2.040	64.317	**<** **0.001**	2.311	159.759	**<** **0.001**	1.817	19.026	**<** **0.001**	2.498	187.458	**<** **0.001**	2.342	100.043	**<** **0.001**	2.452	89.539	**<** **0.001**

*Note*: Significant values are in bold. These covariates included age, sex assigned at birth, gender identity, ethnicity, BMI, only‐child status, education level, smoking status and alcohol consumption, family structure, household income and parental education level. Detailed information on these analyses can be found in tables S8 and S9.

Abbreviations: BMI, body mass index; Chi.sq, chi‐squared test statistic; Edf, estimated degrees of freedom; NSSI, nonsuicidal self‐injury; PTSD, post‐traumatic stress disorder; SA, suicide attempt; SI, suicidal ideation.

^a^ The smooth terms included in the generalised additive model are summarised by their Edf.

### Measures

#### Sociodemographic characteristics

Sociodemographic characteristics collected included age, sex assigned at birth, gender identity, ethnicity, only‐child status, education level, smoking status and alcohol consumption, family structure, household income and parental education level. Body mass index (BMI) was calculated from self‐reported height and weight. These sociodemographic characteristics were included as covariates in the regression analysis.

### Procedures

#### Exercise classification and assessment

Exercise type was determined using a precategorised questionnaire item. Participants selected the single category that best characterised their predominant physical activity from six mutually exclusive options. Participants were asked, ‘What type of physical activity or exercise did you spend most of your time doing?’ with response options: 1 = single anaerobic exercises (e.g., sprinting, high jump, long jump, push‐ups, sit‐ups and weightlifting); 2 = single low‐intensity aerobic exercises (e.g., walking, Tai Chi and yoga); 3 = single moderate‐intensity aerobic exercises (e.g., swimming, running, rope skipping, cycling, aerobics and ice skating); 4 = two‐player racket sports (e.g., table tennis, badminton and tennis); 5 = team ball sports (e.g., volleyball, basketball, football, rugby and baseball); 6 = other (please specify: ______) (see table S1). Participants also reported exercise frequency (number of sessions per week) and typical session duration (minutes or hours per session). Frequency was recorded across four categories: 0–1, 2–3, 4–5 or 6–7 sessions per week. Duration was reported across six intervals: 0–0.5, 0.5–1.0, 1.0–1.5, 1.5–2.0, 2.0–2.5 and 2.5–3.0 h. For dose–response analyses with mental health outcomes, duration categories were recoded to corresponding minute values of 30, 60, 90, 120, 150 and 180 min.

### Outcome variables

#### Nonsuicidal self‐injury

The adapted Clinician‐Rated Severity of Nonsuicidal Self‐Injury (CRS‐NSSI; American Psychiatric Association) was used to measure NSSI. CRS‐NSSI is a single‐item tool assessing the presence and severity of NSSI, with good reliability (weighted Cohen's *κ* = 0.91).^24^ To assess NSSI history, a two‐part question was administered. The first question asked whether participants had intentionally inflicted harm on themselves without suicidal intent. Affirmative responses were followed by a frequency rating of NSSI behaviour over the past year, ranging from 0 to 12 days or more (1 = 0 days, 2 = 1–4 days, 3 = 5–7 days, 4 = 8–11 days, 5 = 12 days or more). NSSI was then coded dichotomously as present or absent based on lifetime history.

#### Suicidality

Suicidal ideation and suicide attempts were measured through the Chinese version of the 4‐item Suicidal Behaviors Questionnaire‐Revised (SBQ‐R). The SBQ‐R is a self‐report scale designed to assess suicidal risk, with good reliability and validity (internal consistency ranging from 0.79 to 0.86, area under the receiver operating characteristic curve = 0.78).^25^ Both outcomes were derived from the first item of the SBQ‐R and coded dichotomously. This item assesses lifetime suicidal ideation and/or attempts, asking whether participants have ever thought about or attempted to kill themselves. Response options included (1) ‘never’, (2) ‘it was just a brief passing thought’, (3a) ‘I have had a plan at least once to kill myself but did not try to do it’, (3b) ‘I have had a plan at least once to kill myself and really wanted to die’, (4a) ‘I have attempted to kill myself, but did not want to die’ and (4b) ‘I have attempted to kill myself, and really hoped to die’.^26^ Participants choosing Option 4 were characterised as having suicide attempts, whereas participants choosing Options 2, 3 and 4 were characterised as having suicidal ideation.

#### Depressive symptoms

The severity of depressive symptoms was measured using the Chinese version of the nine‐item Patient Health Questionnaire, with a cut‐off score of 10.^27^, ^28^

#### Anxiety symptoms

Anxiety symptoms were measured using the seven‐item Generalised Anxiety Disorder scale, with a cut‐off score of 10.^29^

#### Post‐traumatic stress disorder

PTSD symptoms were measured using the Trauma Screening Questionnaire (TSQ). The TSQ has demonstrated good predictive validity for future PTSD, with a sensitivity of 0.85, a specificity of 0.89 and a cut‐off score of 6.^30^

### Statistical analysis

**FIGURE 2:**
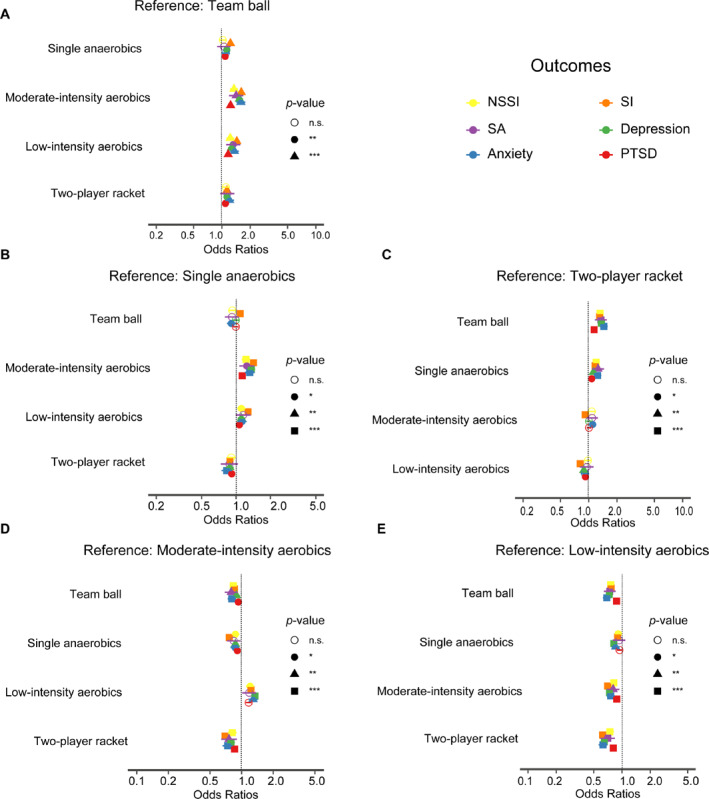
ORs and 95% CIs for mental health outcomes by exercise type. Unadjusted results are presented to illustrate relative differences across exercise types. Each panel uses a different exercise type as the reference category: (A) team ball sports, (B) single anaerobic activities, (C) two‐player racket sports, (D) moderate‐intensity aerobic sports and (E) low‐intensity aerobic sports. CI, confidence interval; n.s., not significant; NSSI, nonsuicidal self‐injury; OR, odds ratio; PTSD, post‐traumatic stress disorder; SA, suicide attempt; SI, suicidal ideation.

Logistic regression analyses were conducted to examine the associations between the five exercise types and mental health outcomes, adjusting for a set of individual‐level covariates. Covariates included age, sex assigned at birth, gender identity, BMI, ethnicity, only‐child status, educational attainment, tobacco and alcohol use, family structure, household income and parental education level. However, individuals engaging in different types of exercise (e.g., team ball sports vs. single low‐intensity aerobic activities) may differ systematically in characteristics such as age or household income. To address this self‐selection bias, we implemented a multinomial propensity‐weighting framework using the R *twang* package. Propensity scores representing each participant's probability of belonging to a given exercise category conditional on the covariate set were estimated via generalised boosted regression trees. These scores were then used to derive the inverse probability of treatment weights, which were winsorised at the 99th percentile to minimise the influence of extreme weights. This approach is consistent with methodological precedents in large‐scale observational health studies (e.g., Chekroud et al.). These final, stabilised weights were then included in all subsequent logistic regression analyses examining the associations between exercise type and mental health outcomes. Odds ratios (ORs) were calculated for each exercise type, using the other types as references, to determine their relative associations with each mental health outcome (figure 2).

**FIGURE 3:**
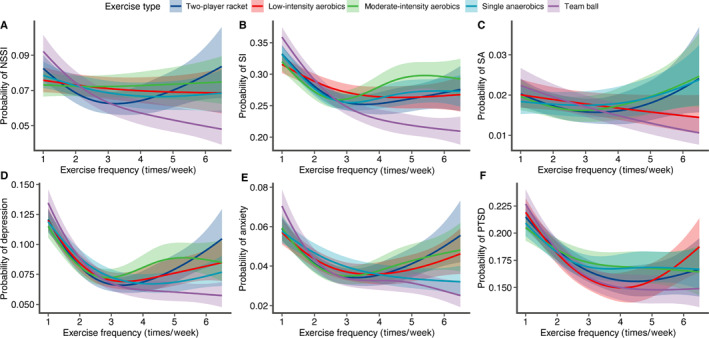
Nonlinear associations between exercise frequency and mental health outcomes estimated by generalised additive models. Solid lines represent smooth functions (splines) for each exercise type after adjusting for all covariates; shaded bands represent 95% CIs. The *y*‐axis is on the probability scale. (A) NSSI, (B) SI, (C) SA, (D) depression, (E) anxiety and (F) PTSD. CI, confidence interval; NSSI, nonsuicidal self‐injury; PTSD, post‐traumatic stress disorder; SA, suicide attempt; SI, suicidal ideation.

We further examined the associations between exercise duration and frequency and multiple mental health outcomes using generalised additive models (GAMs). Penalised cubic regression splines were specified for the smooth terms representing exercise duration and frequency, whereas the complete set of covariates was included as parametric terms. Models were implemented in R using the *mgcv* package, with cubic regression splines as basis functions and an effective basis dimension of *k* = 4. To visualise potential nonlinear relationships, we plotted the estimated smooth functions for duration and frequency with pointwise 95% confidence intervals (CIs), both overall and stratified by exercise type, using smoothed conditional means (figure 3).

Given well‐documented sex differences in mental health prevalence and physical activity patterns, sensitivity analyses stratified by sex were conducted to assess whether the observed associations and dose–response patterns remained consistent across sexes. The reporting of this study adheres to the Strengthening the Reporting of Observational Studies in Epidemiology statement for cross‐sectional studies.^31^

### Equity, diversity and inclusion statement

The group of authors comprises researchers from various stages of their careers, including junior, mid‐career and senior researchers, from diverse disciplinary backgrounds, and demonstrates gender balance. However, all authors are from the same country. The study population of this research includes participants who identify as cisgender, transgender, nonbinary/genderqueer or have doubts about their gender identity. Additionally, participants come from a range of socioeconomic backgrounds and household structures, including foster families, left‐behind families and other household arrangements.

## RESULTS

A total of 85 509 out of 96 218 (88.9%) participants were included in the descriptive statistics. The baseline characteristics of participants reporting engagement in different exercise types are summarised in table 1. Among these, 79 011 (92.4%) participants reported engaging in single anaerobic activities (16 376 [19.2%]), low‐intensity aerobic activities (14 560 [17.0%]), moderate‐intensity aerobic activities (13 059 [15.3%]), two‐player racket sports (20 000 [23.4%]) or team ball sports (15 016 [17.6%]). The 6498 participants (7.6%) who reported engaging in physical activities not specified in the list were excluded from both the logistic regression and GAM analyses. Table S2 outlines exercise frequency, duration and various mental health outcomes across different exercise types. Most two‐player racket sport participants (10 346 [51.7%]) exercised 0–1 times per week, most commonly for 0.5–1.0 h per session (7561 [37.8%]). Similarly, most low‐intensity aerobic exercise participants (6266 [43.0%]) exercised 0–1 times per week, for 0.5–1.0 h per session (5953 [40.9%]). Most moderate‐intensity aerobic (5153 [39.5%]) and single anaerobic exercise participants (6314 [38.6%]) exercised 2–3 times per week, most commonly for 0.5–1.0 h per session (5396 [41.3%] and 6559 [40.1%], respectively). Most team ball sport participants (6321 [42.1%]) also exercised 2–3 times per week, but for longer sessions of 1.0–1.5 h (4417 [29.4%]). Among those reporting other types of exercise, most (3151 [48.5%]) exercised 0–1 times per week for 0–0.5 h per session (2889 [44.5%]).

After adjusting for sociodemographic and health‐behaviour covariates (see table 2 note; tables S3–S7), mental health outcomes differed significantly by exercise type (table 2). With team ball sports as the reference (figure 2A), all other exercise categories showed higher odds of adverse mental health outcomes. The greatest excess risk appeared for low‐intensity aerobic activities, which had substantially elevated odds of depression (OR = 1.63, 95% CI 1.50–1.78, *p* < 0.001) and anxiety (OR = 1.64, 95% CI 1.47–1.84, *p* < 0.001). Moderate‐intensity aerobic activities (e.g., depression: OR = 1.31, 95% CI 1.20–1.42, *p* < 0.001) and single anaerobic activities (e.g., depression: OR = 1.17, 95% CI 1.08–1.27, *p* < 0.001) also carried a significantly higher risk than team ball sports.

When single anaerobic activities served as the reference (figure 2B), low‐intensity aerobic activities (e.g., depression: OR = 1.40, 95% CI 1.29–1.51, *p* < 0.001) and moderate‐intensity aerobic activities (e.g., depression: OR = 1.12, 95% CI 1.04–1.21, *p* = 0.007) continued to show higher odds of adverse outcomes, whereas team ball sports showed a significant protective effect (e.g., depression: OR = 0.86, 95% CI 0.79–0.93, *p* < 0.001).

Relative to two‐player racket sports (figure 2C), low‐intensity aerobic activities (e.g., depression: OR = 1.40, 95% CI 1.30–1.50, *p* < 0.001) and moderate‐intensity aerobic activities (e.g., depression: OR = 1.12, 95% CI 1.04–1.20, *p* = 0.004) likewise showed increased risks of adverse mental health outcomes. When moderate‐intensity aerobic activities served as the reference (figure 2D), all other exercise types demonstrated protective associations: Team ball sports showed the strongest protection (e.g., depression: OR = 0.77, 95% CI 0.70–0.83, *p* < 0.001), followed by single anaerobic activities (e.g., depression: OR = 0.89, 95% CI 0.83–0.97, *p* = 0.007) and two‐player racket sports (e.g., depression: OR = 0.89, 95% CI 0.83–0.96, *p* = 0.004). The contrast was most pronounced when low‐intensity aerobic activities served as the reference category (figure 2E): All four remaining exercise types were significantly protective across all six mental health outcomes (all ORs < 1, *p* < 0.05). Team ball sports again showed the strongest protective association (e.g., depression: OR = 0.61, 95% CI 0.56–0.67, *p* < 0.001; anxiety: OR = 0.61, 95% CI 0.54–0.68, *p* < 0.001).

Accordingly, ranked by protective association with mental health, the exercise types fell in the following order: team ball sports > single anaerobic activities > two‐player racket sports > moderate‐intensity aerobic activities > low‐intensity aerobic activities.

We found significant nonlinear associations between exercise frequency and mental health outcomes, including anxiety, depression, PTSD, suicidal ideation and NSSI, after adjusting for sociodemographic variables. However, these associations were not uniform across all exercise types. Specifically, low‐intensity aerobic exercise frequency was not significantly associated with NSSI (Edf = 1.344, *p* = 0.357) or suicide attempts (Edf = 1.006, *p* = 0.094). Similarly, moderate‐intensity aerobic exercise frequency was not significantly associated with NSSI (Edf = 1.307, *p* = 0.869) or suicide attempts (Edf = 2.088, *p* = 0.089), as shown in table 3 and table S8. As shown in figure 3, increasing team ball sport frequency was monotonically associated with reduced odds of all mental health outcomes (all *p* < 0.001). Moreover, significant U‐shaped associations were observed between exercise frequency and both anxiety and depression for two‐player racket sports, low‐intensity aerobic activities and moderate‐intensity aerobic activities (all *p* < 0.001, all Edf ≥ 2.3). Specifically, individuals exercising three to four times per week had a lower probability of anxiety and depression than those who exercised fewer than three or more than four times, as illustrated in figure 3D, E and figures S2 and S3. Additionally, for two‐player racket (Edf = 2.546, *p* < 0.001), moderate‐intensity aerobic (Edf = 2.891, *p* < 0.001) and single anaerobic (Edf = 2.845, *p* < 0.001) exercise frequencies, we found a U‐shaped relationship with suicidal ideation. Individuals performing these exercises three to four times per week had a lower probability of suicidal ideation than those exercising fewer than three or more than four times, as shown in figure 3B and figure S6.

**FIGURE 4:**
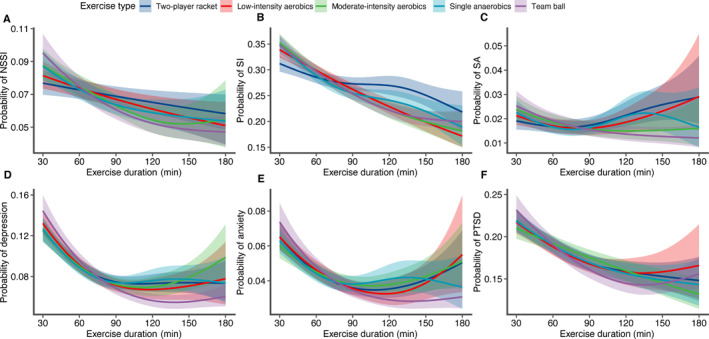
Nonlinear associations between exercise duration and mental health outcomes estimated by generalised additive models. Solid lines represent smooth functions (splines) for each exercise type after adjusting for all covariates; shaded bands represent 95% CIs. The *y*‐axis is on the probability scale. (A) NSSI, (B) SI, (C) SA, (D) depression, (E) anxiety and (F) PTSD. CI, confidence interval; NSSI, nonsuicidal self‐injury; PTSD, post‐traumatic stress disorder; SA, suicide attempt; SI, suicidal ideation.

Similarly, we observed significant nonlinear relationships between all mental health outcomes and exercise duration (all *p* < 0.05) while adjusting for sociodemographic variables, as presented in table 3 and table S9. Figure 4 shows that 150 min per session of team ball sport was associated with the lowest probability of all mental health outcomes (all *p* < 0.001, all Edf > 1.8). Additionally, exercise durations ranging from 90 to 120 min of single anaerobic (e.g., anxiety: Edf = 2.694, *p* < 0.001; depression: Edf = 2.675, *p* < 0.001), low‐intensity aerobic (e.g., anxiety: Edf = 2.499, *p* < 0.001; depression: Edf = 2.249, *p* < 0.001), moderate‐intensity aerobic (e.g., anxiety: Edf = 2.222, *p* < 0.001; depression: Edf = 2.421, *p* < 0.001) and two‐player racket exercises (e.g., anxiety: Edf = 2.391, *p* < 0.001; depression: Edf = 2.551, *p* < 0.001) were associated with the lowest probability of anxiety and depression, with the nadir at around 120 min (figure 4D, E; figures S8 and S9). Furthermore, exercising for 180 min or more per session was associated with the lowest probability of NSSI and SI for most exercise types (all *p* < 0.05) (figure 4A, B; figures S10 and S13). For two‐player racket (Edf = 2.088, *p* < 0.001), single anaerobic (Edf = 1.881, *p* < 0.001) and moderate‐intensity aerobic (Edf = 1.002, *p* < 0.001), exercising for 180 min or more was associated with a decrease in the probability of PTSD, whereas low‐intensity aerobic exercise for 120 min was associated with the lowest probability of PTSD (Edf = 2.106, *p* < 0.001) (figure 4F and figure S11). Finally, exercising for 60–90 min for single anaerobic (Edf = 2.739, *p* = 0.022), low‐intensity aerobic (Edf = 2.064, *p* = 0.034) and two‐player racket sports (Edf = 2.446, *p* = 0.036) was associated with the lowest probability of suicide attempts, whereas exercising for 120 min of moderate‐intensity aerobic exercise was linked with the lowest probability of suicide attempts (Edf = 1.829, *p* = 0.005) (figure 4C and figure S12).

### Sensitivity analyses

Sex‐stratified sensitivity analyses were conducted to verify the robustness of our findings. For the male subgroup, the ranking of protective associations mirrored that of the overall population (e.g., relative to team ball sports, low‐intensity aerobic activities showed the highest odds for depression: OR = 2.13, 95% CI 1.88–2.41, *p* < 0.001; and anxiety: OR = 2.16, 95% CI 1.83–2.54, *p* < 0.001). Conversely, in the female subgroup, two‐player racket sports demonstrated the strongest protective association, followed by single anaerobic activities and team ball sports (which showed comparable effects) and then moderate‐intensity and low‐intensity aerobic activities (e.g., when using low‐intensity aerobic activities as the reference, two‐player racket sports showed the strongest protection against depression: OR = 0.79, 95% CI 0.73–0.85, *p* < 0.001; and anxiety: OR = 0.75, 95% CI 0.68–0.83, *p* < 0.001) (tables S10 and S11).

Regarding the dose–response relationships, the nonlinear trends (predominantly U‐shaped) were largely preserved across sexes, with specific exceptions for severe outcomes. For exercise frequency in females (table S12 and figure S14), significant nonlinear associations were found for depression, anxiety, suicidal ideation and PTSD across almost all exercise types; however, significant nonlinear associations with suicide attempts were observed only for single anaerobic (Edf = 2.683, *p* = 0.006) and team ball sports (Edf = 1.005, *p* = 0.048) and with NSSI only for two‐player racket sports (Edf = 2.218, *p* = 0.009). In males, although most exercise types maintained nonlinear relationships with depression, anxiety, suicidal ideation and PTSD, only two‐player racket (Edf = 2.052, *p* = 0.002) and team ball sports (Edf = 1.003, *p* = 0.012) showed significant nonlinear associations with suicide attempts, and only two‐player racket (Edf = 2.209, *p* < 0.001), single anaerobic (Edf = 1.727, *p* = 0.030) and team ball sports (Edf = 2.434, *p* < 0.001) with NSSI (table S13 and figure S16).

Regarding exercise duration, females exhibited significant nonlinear associations for depression, anxiety, suicidal ideation and PTSD across most types (*p* < 0.05), but only low‐intensity aerobic (Edf = 2.091, *p* = 0.030), moderate‐intensity aerobic (Edf = 1.891, *p* = 0.025) and two‐player racket sports (Edf = 2.011, *p* = 0.042) showed nonlinearity with suicide attempts (refer to table S12 and figure S15). In males, all exercise types displayed significant nonlinear associations with depression, anxiety, suicidal ideation, NSSI and PTSD, with the exception of suicide attempts, for which no significant nonlinear trends were found (all *p* > 0.05; e.g., team ball: *p* = 0.688) (refer to table S13 and figure S17).

Overall, these sensitivity analyses confirm that the primary findings—the hierarchy of protective exercise types and the nonlinear dose–response patterns—are robust across sexes.

## DISCUSSION

### Main findings

This study demonstrates that exercise type is significantly associated with mental health outcomes, even after controlling for various sociodemographic factors. Previous studies have suggested that exercise positively affects mental health outcomes, including depression,^32^, ^33^ anxiety^34^ and PTSD.^35^, ^36^ Findings on suicidal ideation and suicide attempts are mixed: Several studies report significant protective associations,^13^, ^37^ whereas others find no significant link.^38^ Our study further suggests that physical exercise is associated with lower odds of suicidal ideation, suicide attempts and NSSI. In particular, participation in team ball sports, single anaerobic activities and two‐player racket sports was associated with lower odds of adverse mental health outcomes than participation in single, moderate‐ or low‐intensity aerobic activities.

The ‘mental health through sport’ conceptual model explicitly recognises that the physical, mental and social benefits of sport participation vary significantly by its context.^5^ To capture this variation, we classified exercises into five types based on distinct combinations of social complexity and physiological demand. For example, team ball sports represent the highest levels of both social interaction and physiological demand, whereas single low‐intensity aerobic sports involve minimal social interaction and low physiological intensity. Our results are consistent with this hierarchical categorisation: Team ball sports, single anaerobic activities and two‐player racket sports were associated with significantly lower odds of adverse mental health outcomes compared to moderate‐ and low‐intensity aerobic sports. This finding strongly supports the notion that both physical and social mechanisms underlie these protective effects. The observed differences in efficacy can be understood by examining the two primary pathways. Regarding the social and psychosocial pathway, sport participation fosters the development of robust social support networks and enduring social relationships. Empirical evidence confirms that engaging in structured social settings (e.g., group walking or group golf) is directly linked to better self‐rated health and lower depressive symptoms compared to nongroup equivalents, highlighting that social context matters.^22^ Team sports, in particular, enhance psychological resilience and self‐worth through unique mechanisms that go beyond mere co‐presence: belonging and companionship, social support, behavioural guidance, purpose and meaning (e.g., role‐based identity within a team). Our findings that team ball sports provide the most robust protection against mental health disorders resonate with the social‐ecological framework of suicide prevention, which underscores the value of strengthening social support and interpersonal relationships.^39^ Although restlessness and a sad mood are key drivers of suicidal ideation,^2^ the socially interactive nature of team ball sports may directly alleviate these symptoms by fostering resilience and a sense of social connectedness. Regarding the neurobiological and cognitive pathway, exercise affects mental health by stimulating neurobiological systems, enhancing neuroplasticity and improving cognitive control and emotional regulation. Recent research indicates that aerobic sports primarily enhance functional brain connectivity in the primary motor cortex, whereas anaerobic sports demonstrate superior connectivity in the prefrontal lobe.^19^ This distinction is particularly relevant because the prefrontal lobe plays an essential role in cognitive control and emotional regulation, and emotional dysregulation is a core feature implicated in numerous mental disorders.^20^ Consistent with this, participation in team ball sports has been associated with enhanced executive function performance among adolescents.^40^ Together, these findings suggest that different exercise types exert distinct effects on neuroplasticity, supporting integrative models in which exercise improves mental health through both neurobiological and cognitive mechanisms.^16^ In line with our results and the proposed mechanisms, team ball sports (high physical/high social properties) are most strongly linked to reduced mental health problems. Single anaerobic activities and two‐player racket sports also confer high mental health benefits, underscoring the vital role of either high physiological demand or social complexity. Even moderate‐ and low‐intensity aerobic sports, despite showing smaller effect sizes, maintained a significant inverse association with adverse mental health outcomes. Ultimately, this hierarchy aligns with the notion that regular physical exercise increases the salience of rewards by facilitating involvement in purposeful activities or improved physical fitness, which consequently triggers positive affect and enhances motivation for sustained engagement.^41^, ^42^

Regarding the dose effects of different exercise types on mental health, our results suggest that the argument of “more is better” does not always hold true beyond certain volumes. Our results suggest that the argument of ‘more is better’ does not always hold true beyond certain volumes. Recent research indicated that exercise intensity scaled with neuroplasticity in healthy young adults.^43^ We found that extreme ranges of over five times per week or longer than 150 min per session for most types of exercise result in worse mental health. This corresponds to the U‐shaped curves seen for frequency and duration in depression, anxiety and suicidal ideation for most types of exercise. The U‐shaped curves between physical exercise and mental health burden,^4^ such as psychosomatic complaints,^44^ have been observed in previous research. Khan et al. found that physical activity decreases psychosomatic complaints, with benefits plateauing at 5 days per week.^44^ Moreover, Chekroud et al. revealed that 2–6 h per week of exercise was optimal for a lower mental health burden.^4^ The U‐shaped dose–response relationship identified in our research emphasises the importance of ‘optimal’ rather than ‘maximal’ lifestyle modification. This aligns with findings that an improved overall lifestyle, including regular physical activity and healthy dietary habits, serves as a viable intervention to mitigate symptoms of depression and anxiety, provided that it is managed as a balanced strategy.^45^ Our tracking of 79 011 students extends this by quantifying the precise frequency and duration (3–4 times per week; 90–120 min) required to achieve these protective effects.

One important point is that our results show only small effect sizes (ORs = 1–2 and 0.6–1.0). This difference from the large standardised mean differences (SMDs) reported in some meta‐analyses (e.g., aerobic SMD = −1.156; resistance training SMD = −1.042) largely reflects fundamental differences in study design and analytic approach, together with features of the study population and measurement. Our logistic models use propensity score matching weighting and are based on intergroup comparisons in which each exercise category serves as the reference for the others. Thus, the reported ORs represent the modest relative difference in efficacy between two groups of actively exercising individuals. Because all groups are deriving benefit from physical activity, the marginal differences in risk between exercise types are expectedly small, and ORs, therefore, tend to lie close to 1. Moreover, propensity score matching rigorously controls for many demographic and socioeconomic confounders that are often associated with exercise selection. Although this increases internal validity by isolating the exercise‐type effect, it removes noncausal advantages (e.g., higher‐income groups choosing a particular sport and having better mental health for resource‐related reasons), which would otherwise inflate the total effect. Finally, because our sample is drawn from the general population with relatively low baseline mental‐health risk (nonclinical participants), a ceiling effect limits the measurable improvement and further attenuates ORs.

### Limitations

A primary limitation of this study is its cross‐sectional design, which precludes establishing causality for the association between exercise and mental health. Specifically, this design makes it impossible to determine the temporal sequence between exercise and mental health outcomes. We cannot disambiguate, for example, whether engaging in team ball sports is associated with improved mental health or whether individuals with better pre‐existing mental health are simply more likely to select (or have the resources for) team ball sports. This possibility of reverse causality is a critical limitation. Furthermore, our approach only compared the relationship between the primary exercise types and mental health. Although this approach somewhat controls the combined effect of other exercise types, this is also one of the study's limitations because many individuals simultaneously participate in multiple exercise types. Thus, the cumulative effects of each type and their respective exercise frequency and duration patterns may have varying consequences for dimensions of psychological well‐being.^46^ Secondly, the absence of an inactive category limits the study's conclusions, as the results only demonstrate relative differences across exercise categories. Although several randomised controlled trials have demonstrated a causal relationship between physical activity and mental health,^47^ this study did not directly compare differences between physical activity categories and an inactive group.

Furthermore, it is important to note that the measurement of exercise in this study was solely based on self‐reports. Although this method is common, self‐reports are susceptible to recall bias and may not accurately quantify the true frequency and duration of the activity. This imprecision presents a significant challenge, as it complicates the interpretation of the underlying mechanisms driving our observed associations. For example, it is difficult to disentangle whether the strong protective association of team ball sports is primarily due to its typical high intensity (a physiological effect) or its inherent social component. Future studies should therefore utilise passive mobile or wearable sensor data (e.g., accelerometers) to objectively investigate the actual frequency, duration and especially intensity of exercise. Such objective data would be invaluable for elucidating the specific physiological versus psychosocial mechanisms by which different exercise types relate to mental health. Finally, to address these limitations, future research should employ longitudinal designs to clarify temporal relationships. Furthermore, where feasible, researchers should consider applying instrumental variable analysis to better address endogeneity and further validate these causal pathways.

## CONCLUSIONS

Our study suggests that participating in various sports can enhance psychological well‐being. Team ball sports appear to have a stronger positive impact on mental health compared to single anaerobic activities, followed by two‐player racket sports, single moderate‐intensity aerobic sports, and lastly, single low‐intensity aerobic sports. Moreover, exercise dosage appears to be a critical factor, as the optimal dosage varies across exercise types.

### Implications

This study provides critical empirical validation for the heterogeneity of exercise efficacy, fundamentally challenging traditional models that treat physical activity as a homogeneous factor. The hierarchy established here shows that team ball sports and single anaerobic activities confer superior mental health benefits compared to low‐intensity activities. Our results challenge the nonspecific assumption that ‘more exercise is always better’. They enable practitioners to define a ‘sweet spot’ for maximising mental health benefits while minimising the risks of overtraining or relapse. Specifically, our findings suggest that exercising three to four times per week for 90–120 min per session represents an optimal dosage for maximising mental health benefits.

## AUTHOR CONTRIBUTIONS

Huagen Wang: research design, data analysis, drafting the manuscript. Yisheng Aku: writing and editing. Shicun Xu: conducting data collection. Zhanbing Ren and Yingjie Zhang: guidance on the conceptual classification, such as exercise type. Runsen Chen and Jing An: review.

## FUNDING

This work was supported by the Youth Fund Project of Humanities and Social Sciences of the Ministry of Education of China (Grant No. 25YJCZH250) and ‘the Fundamental Research Funds for the Central Universities’ (Grant No. XJJSKYQD202501).

## CONFLICT OF INTEREST STATEMENT

The authors declare no conflicts of interest.

## ETHICS STATEMENT

This study was approved by the Ethics Committee (ID: 2021929).

## Supporting information

Supporting Information S1

Supporting Information S2

## Data Availability

The dataset for this specific manuscript is available from the corresponding author upon request.
