# Supplementary material for: Exercise type, dose and mental health outcomes in youth: Which types and doses are sufficient?
Source: Gen Psychiatr. 2026 Jun 12;39(3):e70031. doi: 10.1002/gps3.70031 (PMC13261303; doi:10.1002/gps3.70031)

## Supplementary File

## S Table1.

Five categories of exercise: single anaerobic exercises, single low-intensity aerobic exercises, single moderate-intensity aerobic exercises, two-player swing-type exercises, and team ball exercises.

| Activity group | Activity name |
| --- | --- |
| Single anaerobic exercises  (N=16 376) | Sprinting,  high jump,  long jump,  push-ups,  sit-ups,  weight  lifting |
| Single low-intensity aerobic exercises  (N=14 560) | Walking,  Tai chi,  Yoga |
| Single moderate-intensity aerobic exercises  (N=13 059) | Swimming,  running,  rope skipping,  cycling,  aerobics,  skating |
| Two-player racket sports  (double swing) (N=20 000) | Table tennis,  badminton,  tennis |
| Team ball sports  (N=15 016) | Volleyball,  basketball,  soccer,  soccer,  rugby,  baseball |
| Others  (N=6498) | - |

**S Table 2:** Frequency, duration, and mental health outcomes of participants based on different types of exercise.

| **Characteristic** | **Others**  N = 6498^1^ | **Single anaerobics**  N = 16 376^1^ | **Low-intensity aerobics**  N = 14 560^1^ | **Medium-intensity aerobics**  N = 13 059^1^ | **Two-player racket**   N = 20 000^1^ | **Team ball**  N = 15 016^1^ | ***P* value**^2^ | **Statistic** |
| --- | --- | --- | --- | --- | --- | --- | --- | --- |
| Exercise frequency |  |  |  |  |  |  | < 0.001 | X^2^ = 3957.55 |
| 0–1 times/week | 3151 (48%) | 5764 (35%) | 6266 (43%) | 4888 (37%) | 10 346 (52%) | 4370 (29%) |  |  |
| 2–3 times/week | 1612 (25%) | 6314 (39%) | 5708 (39%) | 5153 (39%) | 7568 (38%) | 6321 (42%) |  |  |
| 4–5 times/week | 838 (13%) | 2695 (16%) | 1644 (11%) | 2075 (16%) | 1532 (7.7%) | 2757 (18%) |  |  |
| 6–7 times/week | 897 (14%) | 1603 (9.8%) | 942 (6.5%) | 943 (7.2%) | 554 (2.8%) | 1568 (10%) |  |  |
| Exercise duration |  |  |  |  |  |  | < 0.001 | X^2^ = 12 606.43 |
| 0–0.5 h | 2889 (44%) | 5204 (32%) | 5217 (36%) | 3669 (28%) | 4105 (21%) | 1433 (9.5%) |  |  |
| 0.5–1 h | 1573 (24%) | 6559 (40%) | 5953 (41%) | 5396 (41%) | 7561 (38%) | 3303 (22%) |  |  |
| 1–1.5 h | 914 (14%) | 2890 (18%) | 2512 (17%) | 2465 (19%) | 5300 (27%) | 4417 (29%) |  |  |
| 1.5–2 h | 496 (7.6%) | 1099 (6.7%) | 616 (4.2%) | 1023 (7.8%) | 2102 (11%) | 3148 (21%) |  |  |
| 2–2.5 h | 180 (2.8%) | 275 (1.7%) | 112 (0.8%) | 249 (1.9%) | 499 (2.5%) | 1267 (8.4%) |  |  |
| 2.5–3 h | 446 (6.9%) | 349 (2.1%) | 150 (1.0%) | 257 (2.0%) | 433 (2.2%) | 1448 (9.6%) |  |  |
| Depression score (PHQ-9) | 6.6 (5.4) | 4.8 (4.4) | 5.5 (4.5) | 5.1 (4.4) | 5.0 (4.2) | 4.7 (4.3) | < 0.001 | F = 173.61 |
| Depressive symptom | 1357 (21%) | 1789 (11%) | 1927 (13%) | 1546 (12%) | 2136 (11%) | 1440 (9.6%) | < 0.001 | X^2^ = 643.58 |
| Anxiety score  (GAD-7) | 4.6 (4.5) | 3.5 (3.9) | 4.0 (3.9) | 3.8 (3.9) | 3.6 (3.7) | 3.3 (3.7) | < 0.001 | F = 126.94 |
| Anxiety symptom | 754 (12%) | 1016 (6.2%) | 1116 (7.7%) | 927 (7.1%) | 1139 (5.7%) | 762 (5.1%) | < 0.001 | X^2^ = 378.05 |
| PTSD score  (TSQ) | 3.40 (2.99) | 2.67 (2.90) | 2.89 (2.85) | 2.86 (2.87) | 2.77 (2.82) | 2.61 (2.85) | < 0.001 | F = 76.84 |
| PTSD symptom | 1684 (26%) | 3103 (19%) | 2951 (20%) | 2620 (20%) | 3757 (19%) | 2712 (18%) | < 0.001 | X^2^ = 204.84 |
| Lifetime NSSI | 904 (14%) | 1305 (8.0%) | 1415 (9.7%) | 1219 (9.3%) | 1508 (7.5%) | 1046 (7.0%) | < 0.001 | X^2^ = 351.15 |
| Lifetime SI | 2553 (39%) | 4025 (25%) | 4815 (33%) | 4116 (32%) | 5495 (27%) | 3153 (21%) | < 0.001 | X^2^ = 1130.83 |
| Lifetime SA | 346 (5.3%) | 373 (2.3%) | 420 (2.9%) | 370 (2.8%) | 434 (2.2%) | 271 (1.8%) | < 0.001 | X^2^ = 257.78 |
| ^1^n (%); Mean (SD) | | | | | | | | |
| ^2^Pearson's Chi-squared test; Kruskal-Wallis rank sum test  Abbreviations: BMI, body mass index; GAD-7, Generalised Anxiety Disorder scale; NSSI, non-suicidal self-injury; PHQ-9, Patient Health Questionnaire; PTSD, post-traumatic stress disorder; SA, suicide attempt; SD, standard deviation; SI, suicidal ideation; TSQ, Trauma Screening Questionnaire. | | | | | | | | |

| S Table 3. Exercise type, reference level: team ball. | | | | | | | | | | | | | | | | | | |
| --- | --- | --- | --- | --- | --- | --- | --- | --- | --- | --- | --- | --- | --- | --- | --- | --- | --- | --- |
|  | **Lifetime NSSI** | | | **Lifetime SI** | | | **Lifetime SA** | | | **Depression** | | | **Anxiety** | | | **PTSD** | | |
| *Predictors* | *Odds ratios* | *CI (95%)* | *P value (fdr)* | *Odds ratios* | *CI (95%)* | *P value (fdr)* | *Odds ratios* | *CI (95%)* | *P value (fdr)* | *Odds ratios* | *CI (95%)* | *P value (fdr)* | *Odds ratios* | *CI (95%)* | *P value (fdr)* | *Odds ratios* | *CI (95%)* | *P value (fdr)* |
| Exercise type (double swing) | 1.05 | 0.96–1.15 | 0.491 | 1.24 | 1.18–1.32 | **< 0.001** | 1.08 | 0.92–1.28 | 0.428 | 1.17 | 1.08–1.26 | **< 0.001** | 1.12 | 1.01–1.25 | 0.067 | 1.11 | 1.05–1.18 | **0.002** |
| Exercise type (low intensity aerobics) | 1.40 | 1.27–1.55 | **< 0.001** | 1.62 | 1.52–1.72 | **< 0.001** | 1.47 | 1.23–1.75 | **< 0.001** | 1.63 | 1.50–1.78 | **< 0.001** | 1.64 | 1.47–1.84 | **< 0.001** | 1.29 | 1.20–1.38 | **< 0.001** |
| Exercise type (medium intensity aerobics) | 1.25 | 1.14–1.38 | **< 0.001** | 1.45 | 1.37–1.54 | **< 0.001** | 1.35 | 1.13–1.61 | **0.002** | 1.31 | 1.20–1.42 | **< 0.001** | 1.38 | 1.24–1.54 | **< 0.001** | 1.19 | 1.12–1.28 | **< 0.001** |
| Exercise type (single anaerobics) | 1.14 | 1.04–1.25 | **0.016** | 1.15 | 1.09–1.22 | **< 0.001** | 1.18 | 0.99–1.41 | 0.104 | 1.17 | 1.08–1.27 | **< 0.001** | 1.24 | 1.11–1.38 | **< 0.001** | 1.12 | 1.05–1.19 | **0.002** |
| Education level (Postgraduate) | 1.08 | 0.89–1.30 | 0.635 | 1.00 | 0.88–1.12 | 0.956 | 1.17 | 0.83–1.63 | 0.447 | 1.08 | 0.91–1.27 | 0.432 | 1.03 | 0.84–1.27 | 0.862 | 1.00 | 0.88–1.15 | 0.966 |
| Education level (Doctoral candidate) | 0.85 | 0.41–1.78 | 0.757 | 0.82 | 0.49–1.37 | 0.579 | 1.78 | 0.60–5.29 | 0.396 | 0.36 | 0.14–0.92 | **0.049** | 0.35 | 0.12–1.00 | 0.105 | 1.10 | 0.65–1.86 | 0.780 |
| Age | 1.00 | 0.98–1.02 | 0.817 | 0.99 | 0.98–1.00 | 0.319 | 0.98 | 0.94–1.02 | 0.396 | 0.99 | 0.97–1.01 | 0.232 | 1.00 | 0.98–1.02 | 0.977 | 0.99 | 0.98–1.01 | 0.336 |
| BMI | 0.99 | 0.99–1.00 | **0.022** | 1.00 | 0.99–1.00 | 0.053 | 0.99 | 0.98–1.00 | **0.018** | 1.00 | 0.99–1.00 | 0.116 | 1.00 | 0.99–1.01 | 0.862 | 1.00 | 1.00–1.00 | 0.700 |
| Gender (Female) | 1.73 | 1.61–1.85 | **< 0.001** | 1.66 | 1.59–1.72 | **< 0.001** | 2.03 | 1.78–2.31 | **< 0.001** | 1.19 | 1.12–1.26 | **< 0.001** | 1.21 | 1.12–1.30 | **< 0.001** | 1.26 | 1.20–1.31 | **< 0.001** |
| Gender identification (Transgender) | 2.39 | 2.01–2.83 | **< 0.001** | 2.01 | 1.77–2.28 | **< 0.001** | 1.88 | 1.41–2.52 | **< 0.001** | 2.40 | 2.06–2.80 | **< 0.001** | 2.74 | 2.29–3.28 | **< 0.001** | 1.84 | 1.61–2.12 | **< 0.001** |
| Gender identification (Nonbinary or genderquee) | 3.91 | 3.41–4.47 | **< 0.001** | 3.28 | 2.92–3.68 | **< 0.001** | 3.64 | 2.93–4.51 | **< 0.001** | 2.98 | 2.61–3.40 | **< 0.001** | 3.03 | 2.60–3.54 | **< 0.001** | 2.45 | 2.17–2.76 | **< 0.001** |
| Gender identification (Uncertainty) | 2.04 | 1.71–2.44 | **< 0.001** | 1.74 | 1.52–1.98 | **< 0.001** | 1.98 | 1.46–2.69 | **< 0.001** | 2.17 | 1.86–2.54 | **< 0.001** | 1.92 | 1.58–2.34 | **< 0.001** | 1.90 | 1.66–2.17 | **< 0.001** |
| Gender identification (Questioning) | 1.08 | 0.94–1.24 | 0.479 | 0.87 | 0.80–0.95 | **0.006** | 1.15 | 0.91–1.45 | 0.366 | 1.20 | 1.07–1.34 | **0.002** | 1.08 | 0.93–1.26 | 0.502 | 1.09 | 0.99–1.20 | 0.104 |
| Ethnicity (Han) | 1.03 | 0.94–1.13 | 0.701 | 1.02 | 0.96–1.08 | 0.623 | 0.96 | 0.82–1.12 | 0.629 | 0.95 | 0.88–1.03 | 0.304 | 0.97 | 0.88–1.07 | 0.767 | 0.98 | 0.92–1.05 | 0.722 |
| Family category (Extended family [three generations or more]) | 0.98 | 0.91–1.06 | 0.744 | 1.00 | 0.96–1.05 | 0.956 | 0.97 | 0.84–1.12 | 0.733 | 0.98 | 0.92–1.05 | 0.655 | 1.03 | 0.94–1.12 | 0.767 | 1.05 | 1.00–1.11 | 0.088 |
| Family category (Foster/Adoptive family) | 1.81 | 1.04–3.15 | 0.077 | 1.79 | 1.17–2.74 | **0.015** | 1.86 | 0.79–4.38 | 0.262 | 2.12 | 1.37–3.27 | **< 0.001** | 2.26 | 1.35–3.78 | **0.005** | 1.33 | 0.83–2.13 | 0.336 |
| Family category (Blended family) | 1.72 | 1.50–1.97 | **< 0.001** | 1.71 | 1.55–1.89 | **< 0.001** | 1.89 | 1.51–2.36 | **< 0.001** | 1.70 | 1.49–1.92 | **< 0.001** | 1.58 | 1.35–1.86 | **< 0.001** | 1.52 | 1.37–1.70 | **< 0.001** |
| Family category (Single-parent family) | 1.52 | 1.38–1.68 | **< 0.001** | 1.54 | 1.43–1.65 | **< 0.001** | 1.46 | 1.23–1.74 | **< 0.001** | 1.42 | 1.29–1.56 | **< 0.001** | 1.33 | 1.18–1.50 | **< 0.001** | 1.32 | 1.22–1.43 | **< 0.001** |
| Family category (Left-behind family) | 2.07 | 1.71–2.51 | **< 0.001** | 2.23 | 1.95–2.55 | **< 0.001** | 1.93 | 1.42–2.60 | **< 0.001** | 1.74 | 1.47–2.07 | **< 0.001** | 1.82 | 1.47–2.25 | **< 0.001** | 1.46 | 1.26–1.70 | **< 0.001** |
| Fathers education level (Primary school graduation) | 0.69 | 0.53–0.90 | **0.016** | 0.99 | 0.82–1.19 | 0.956 | 0.54 | 0.35–0.85 | **0.018** | 0.67 | 0.53–0.84 | **< 0.001** | 0.93 | 0.67–1.28 | 0.809 | 0.94 | 0.76–1.15 | 0.625 |
| Fathers education level (Junior high school graduation) | 0.62 | 0.47–0.81 | **0.002** | 0.85 | 0.71–1.03 | 0.143 | 0.44 | 0.28–0.69 | **< 0.001** | 0.57 | 0.45–0.72 | **< 0.001** | 0.79 | 0.57–1.10 | 0.305 | 0.87 | 0.71–1.07 | 0.288 |
| Fathers education level (High school/Technical school graduation) | 0.66 | 0.50–0.87 | **0.009** | 0.90 | 0.75–1.10 | 0.415 | 0.44 | 0.28–0.70 | **< 0.001** | 0.57 | 0.45–0.73 | **< 0.001** | 0.84 | 0.60–1.17 | 0.502 | 0.91 | 0.74–1.13 | 0.529 |
| Fathers education level (Associate degree graduation) | 0.74 | 0.56–0.99 | 0.089 | 1.04 | 0.86–1.27 | 0.772 | 0.52 | 0.32–0.84 | **0.018** | 0.61 | 0.48–0.79 | **< 0.001** | 0.87 | 0.61–1.23 | 0.639 | 0.92 | 0.74–1.14 | 0.542 |
| Fathers education level (Bachelor's degree graduation) | 0.70 | 0.52–0.94 | **0.039** | 1.08 | 0.89–1.33 | 0.570 | 0.55 | 0.33–0.89 | **0.034** | 0.68 | 0.53–0.89 | **0.006** | 0.92 | 0.65–1.32 | 0.809 | 0.92 | 0.73–1.15 | 0.544 |
| Fathers education level (Master's/Ph.D./ Postdoctoral graduation) | 0.77 | 0.51–1.17 | 0.392 | 0.99 | 0.75–1.31 | 0.956 | 0.41 | 0.20–0.86 | **0.038** | 0.92 | 0.64–1.32 | 0.655 | 1.52 | 0.96–2.40 | 0.147 | 0.99 | 0.72–1.35 | 0.961 |
| Fathers education level (Not sure) | 0.83 | 0.58–1.18 | 0.479 | 0.92 | 0.72–1.19 | 0.644 | 0.71 | 0.38–1.34 | 0.396 | 0.86 | 0.62–1.18 | 0.396 | 0.84 | 0.55–1.31 | 0.648 | 1.05 | 0.80–1.38 | 0.783 |
| Fathers education level (Others [father's role missing, such as father passing away, etc.]) | 0.74 | 0.54–1.01 | 0.114 | 1.01 | 0.81–1.26 | 0.956 | 0.59 | 0.35–0.99 | 0.087 | 0.68 | 0.52–0.90 | **0.011** | 1.03 | 0.70–1.50 | 0.958 | 1.02 | 0.80–1.30 | 0.938 |
| Mothers education level (Primary school graduation) | 0.94 | 0.77–1.14 | 0.685 | 0.89 | 0.79–1.01 | 0.123 | 1.10 | 0.79–1.54 | 0.627 | 0.96 | 0.81–1.13 | 0.648 | 0.96 | 0.77–1.19 | 0.816 | 0.90 | 0.79–1.04 | 0.230 |
| Mothers education level (Junior high school graduation) | 0.85 | 0.69–1.03 | 0.179 | 0.80 | 0.70–0.90 | **< 0.001** | 0.82 | 0.59–1.16 | 0.381 | 0.86 | 0.72–1.01 | 0.104 | 0.85 | 0.68–1.05 | 0.263 | 0.81 | 0.71–0.93 | **0.005** |
| Mothers education level (High school/Technical school graduation) | 0.92 | 0.75–1.13 | 0.618 | 0.82 | 0.72–0.93 | **0.006** | 0.90 | 0.63–1.28 | 0.615 | 0.87 | 0.73–1.04 | 0.172 | 0.88 | 0.70–1.11 | 0.491 | 0.74 | 0.64–0.85 | **< 0.001** |
| Mothers education level (Associate degree graduation) | 1.06 | 0.85–1.32 | 0.744 | 0.88 | 0.76–1.01 | 0.123 | 0.87 | 0.59–1.28 | 0.557 | 0.90 | 0.74–1.09 | 0.329 | 1.01 | 0.79–1.30 | 0.959 | 0.80 | 0.69–0.94 | **0.012** |
| Mothers education level (Bachelor's degree graduation) | 1.07 | 0.85–1.36 | 0.710 | 0.77 | 0.67–0.90 | **0.002** | 0.93 | 0.61–1.40 | 0.733 | 0.84 | 0.68–1.03 | 0.116 | 0.93 | 0.72–1.21 | 0.770 | 0.77 | 0.66–0.91 | **0.005** |
| Mothers education level (Master's/Ph.D./ Postdoctoral graduation) | 1.24 | 0.83–1.84 | 0.479 | 0.99 | 0.76–1.29 | 0.956 | 1.55 | 0.81–2.98 | 0.301 | 0.80 | 0.55–1.15 | 0.276 | 1.02 | 0.66–1.57 | 0.959 | 0.88 | 0.65–1.18 | 0.529 |
| Mothers education level (Not sure) | 0.98 | 0.72–1.33 | 0.884 | 0.83 | 0.68–1.02 | 0.123 | 0.67 | 0.36–1.22 | 0.301 | 0.89 | 0.67–1.17 | 0.432 | 0.83 | 0.57–1.19 | 0.502 | 0.60 | 0.48–0.75 | **< 0.001** |
| Mothers education level (Others [mother's role missing, such as mother passing away, etc.]) | 1.02 | 0.76–1.38 | 0.884 | 1.13 | 0.93–1.39 | 0.309 | 1.19 | 0.74–1.91 | 0.557 | 1.10 | 0.86–1.41 | 0.496 | 0.97 | 0.69–1.34 | 0.916 | 0.92 | 0.74–1.14 | 0.542 |
| Household income￥6000–14 000 | 1.01 | 0.94–1.08 | 0.865 | 1.05 | 1.01–1.10 | **0.049** | 0.84 | 0.74–0.96 | **0.020** | 0.88 | 0.82–0.93 | **< 0.001** | 0.90 | 0.83–0.98 | **0.027** | 0.94 | 0.89–0.98 | **0.019** |
| Household income￥14 000–23 000 | 1.01 | 0.93–1.11 | 0.817 | 1.10 | 1.04–1.16 | **< 0.001** | 0.93 | 0.80–1.08 | 0.428 | 0.83 | 0.77–0.90 | **< 0.001** | 0.83 | 0.76–0.92 | **< 0.001** | 0.87 | 0.81–0.92 | **< 0.001** |
| Household income￥23 000–36 000 | 0.98 | 0.88–1.09 | 0.757 | 1.08 | 1.01–1.15 | **0.039** | 0.82 | 0.67–0.99 | 0.082 | 0.81 | 0.73–0.89 | **< 0.001** | 0.94 | 0.84–1.06 | 0.502 | 0.87 | 0.81–0.94 | **< 0.001** |
| Household income￥36 000–70 000 | 1.04 | 0.93–1.18 | 0.668 | 1.09 | 1.01–1.18 | **0.045** | 0.87 | 0.69–1.09 | 0.339 | 0.75 | 0.67–0.83 | **< 0.001** | 0.81 | 0.70–0.93 | **0.007** | 0.90 | 0.83–0.98 | **0.031** |
| Household income (＞￥70,000) | 0.96 | 0.83–1.11 | 0.723 | 0.97 | 0.89–1.07 | 0.674 | 1.04 | 0.81–1.34 | 0.757 | 0.79 | 0.69–0.90 | **< 0.001** | 1.03 | 0.89–1.21 | 0.809 | 0.90 | 0.82–1.00 | 0.092 |
| Drinking (≤1 time/month) | 1.60 | 1.49–1.71 | **< 0.001** | 1.45 | 1.39–1.51 | **< 0.001** | 1.29 | 1.14–1.45 | **< 0.001** | 1.33 | 1.25–1.40 | **< 0.001** | 1.14 | 1.06–1.23 | **< 0.001** | 1.38 | 1.32–1.45 | **< 0.001** |
| Drinking 2–4 times/month | 2.59 | 2.36–2.85 | **< 0.001** | 1.79 | 1.68–1.90 | **< 0.001** | 2.08 | 1.76–2.46 | **< 0.001** | 1.96 | 1.80–2.13 | **< 0.001** | 1.66 | 1.49–1.85 | **< 0.001** | 1.90 | 1.78–2.03 | **< 0.001** |
| Drinking 2–3 times/week | 3.30 | 2.79–3.90 | **< 0.001** | 2.17 | 1.92–2.47 | **< 0.001** | 3.18 | 2.41–4.18 | **< 0.001** | 2.99 | 2.58–3.46 | **< 0.001** | 2.74 | 2.29–3.29 | **< 0.001** | 2.65 | 2.33–3.01 | **< 0.001** |
| Drinking (≥4 times/week) | 3.30 | 2.69–4.06 | **<0 .001** | 2.03 | 1.71–2.41 | **< 0.001** | 4.03 | 2.97–5.45 | **< 0.001** | 2.98 | 2.47–3.60 | **< 0.001** | 2.81 | 2.24–3.52 | **< 0.001** | 2.94 | 2.49–3.47 | **< 0.001** |
| Smoking (Yes) | 1.82 | 1.67–1.99 | **< 0.001** | 1.14 | 1.08–1.21 | **< 0.001** | 1.94 | 1.66–2.27 | **< 0.001** | 1.70 | 1.58–1.84 | **< 0.001** | 1.46 | 1.32–1.61 | **< 0.001** | 1.53 | 1.44–1.62 | **< 0.001** |
| Observations | 79 011 | | | 79 011 | | | 79 011 | | | 79 011 | | | 79 011 | | | 79 011 | | |
| R^2^/R^2^ adjusted | 0.052/0.052 | | | 0.035/0.035 | | | 0.050/0.049 | | | 0.042/0.041 | | | 0.032/0.032 | | | 0.027/0.026 | | |

Abbreviations: CI, confidence interval; FDR, false discovery rate; NSSI, non-suicidal self-injury; PTSD, post-traumatic stress disorder; SA, suicide attempt; SI, suicidal ideation.

| S Table 4. Exercise type, reference level: double swing. | | | | | | | | | | | | | | | | | | |
| --- | --- | --- | --- | --- | --- | --- | --- | --- | --- | --- | --- | --- | --- | --- | --- | --- | --- | --- |
|  | **Lifetime NSSI** | | | **Lifetime SI** | | | **Lifetime SA** | | | **Depression** | | | **Anxiety** | | | **PTSD** | | |
| *Predictors* | *Odds ratios* | *CI (95%)* | *P value (fdr)* | *Odds ratios* | *CI (95%)* | *P value (fdr)* | *Odds ratios* | *CI (95%)* | *P value (fdr)* | *Odds ratios* | *CI (95%)* | *P value (fdr)* | *Odds ratios* | *CI (95%)* | *P value (fdr)* | *Odds ratios* | *CI (95%)* | *P value (fdr)* |
| Exercise type (low intensity aerobics) | 1.34 | 1.23–1.45 | **< 0.001** | 1.30 | 1.24–1.37 | **< 0.001** | 1.35 | 1.16–1.57 | **< 0.001** | 1.40 | 1.30–1.50 | **< 0.001** | 1.46 | 1.33–1.61 | **< 0.001** | 1.16 | 1.09–1.23 | **< 0.001** |
| Exercise type (medium intensity aerobics) | 1.19 | 1.10–1.30 | **< 0.001** | 1.17 | 1.11–1.23 | **< 0.001** | 1.24 | 1.08–1.44 | **0.009** | 1.12 | 1.04–1.20 | **0.004** | 1.23 | 1.12–1.35 | **< 0.001** | 1.07 | 1.01–1.14 | **0.028** |
| Exercise type (single anaerobics) | 1.09 | 1.01–1.18 | 0.078 | 0.93 | 0.88–0.97 | **0.005** | 1.09 | 0.94–1.26 | 0.366 | 1.00 | 0.93–1.07 | 0.966 | 1.10 | 1.01–1.20 | 0.078 | 1.00 | 0.95–1.06 | 0.938 |
| Exercise type (team ball) | 0.95 | 0.87–1.05 | 0.491 | 0.80 | 0.76–0.85 | **< 0.001** | 0.92 | 0.78–1.09 | 0.428 | 0.86 | 0.79–0.93 | **< 0.001** | 0.89 | 0.80–0.99 | 0.071 | 0.90 | 0.85–0.96 | **0.002** |
| Education level (Postgraduate) | 1.08 | 0.89–1.30 | 0.635 | 1.00 | 0.88–1.12 | 0.956 | 1.17 | 0.83–1.63 | 0.447 | 1.08 | 0.91–1.27 | 0.443 | 1.03 | 0.84–1.27 | 0.862 | 1.00 | 0.88–1.15 | 0.966 |
| Education level (Doctoral candidate) | 0.85 | 0.41–1.78 | 0.757 | 0.82 | 0.49–1.37 | 0.579 | 1.78 | 0.60–5.29 | 0.396 | 0.36 | 0.14–0.92 | 0.050 | 0.35 | 0.12–1.00 | 0.105 | 1.10 | 0.65–1.86 | 0.799 |
| Age | 1.00 | 0.98–1.02 | 0.817 | 0.99 | 0.98–1.00 | 0.319 | 0.98 | 0.94–1.02 | 0.396 | 0.99 | 0.97–1.01 | 0.238 | 1.00 | 0.98–1.02 | 0.977 | 0.99 | 0.98–1.01 | 0.346 |
| BMI | 0.99 | 0.99–1.00 | **0.023** | 1.00 | 0.99–1.00 | 0.053 | 0.99 | 0.98–1.00 | **0.018** | 1.00 | 0.99–1.00 | 0.119 | 1.00 | 0.99–1.01 | 0.862 | 1.00 | 1.00–1.00 | 0.718 |
| Gender (Female) | 1.73 | 1.61–1.85 | **< 0.001** | 1.66 | 1.59–1.72 | **< 0.001** | 2.03 | 1.78–2.31 | **< 0.001** | 1.19 | 1.12–1.26 | **< 0.001** | 1.21 | 1.12–1.30 | **< 0.001** | 1.26 | 1.20–1.31 | **< 0.001** |
| Gender identification (Transgender) | 2.39 | 2.01–2.83 | **< 0.001** | 2.01 | 1.77–2.28 | **< 0.001** | 1.88 | 1.41–2.52 | **< 0.001** | 2.40 | 2.06–2.80 | **< 0.001** | 2.74 | 2.29–3.28 | **< 0.001** | 1.84 | 1.61–2.12 | **< 0.001** |
| Gender identification (Nonbinary or genderquee) | 3.91 | 3.41–4.47 | **< 0.001** | 3.28 | 2.92–3.68 | **< 0.001** | 3.64 | 2.93–4.51 | **< 0.001** | 2.98 | 2.61–3.40 | **< 0.001** | 3.03 | 2.60–3.54 | **< 0.001** | 2.45 | 2.17–2.76 | **< 0.001** |
| Gender identification (Uncertainty) | 2.04 | 1.71–2.44 | **< 0.001** | 1.74 | 1.52–1.98 | **< 0.001** | 1.98 | 1.46–2.69 | **< 0.001** | 2.17 | 1.86–2.54 | **< 0.001** | 1.92 | 1.58–2.34 | **< 0.001** | 1.90 | 1.66–2.17 | **< 0.001** |
| Gender identification (Questioning) | 1.08 | 0.94–1.24 | 0.479 | 0.87 | 0.80–0.95 | **0.006** | 1.15 | 0.91–1.45 | 0.366 | 1.20 | 1.07–1.34 | **0.003** | 1.08 | 0.93–1.26 | 0.502 | 1.09 | 0.99–1.20 | 0.108 |
| Ethnicity (Han) | 1.03 | 0.94–1.13 | 0.701 | 1.02 | 0.96–1.08 | 0.623 | 0.96 | 0.82–1.12 | 0.629 | 0.95 | 0.88–1.03 | 0.312 | 0.97 | 0.88–1.07 | 0.767 | 0.98 | 0.92–1.05 | 0.740 |
| Family category (Extended family [three generations or more]) | 0.98 | 0.91–1.06 | 0.744 | 1.00 | 0.96–1.05 | 0.956 | 0.97 | 0.84–1.12 | 0.733 | 0.98 | 0.92–1.05 | 0.670 | 1.03 | 0.94–1.12 | 0.767 | 1.05 | 1.00–1.11 | 0.092 |
| Family category (Foster/Adoptive family) | 1.81 | 1.04–3.15 | 0.078 | 1.79 | 1.17–2.74 | **0.015** | 1.86 | 0.79–4.38 | 0.272 | 2.12 | 1.37–3.27 | **< 0.001** | 2.26 | 1.35–3.78 | **0.005** | 1.33 | 0.83–2.13 | 0.346 |
| Family category (Blended family) | 1.72 | 1.50–1.97 | **< 0.001** | 1.71 | 1.55–1.89 | **< 0.001** | 1.89 | 1.51–2.36 | **< 0.001** | 1.70 | 1.49–1.92 | **< 0.001** | 1.58 | 1.35–1.86 | **< 0.001** | 1.52 | 1.37–1.70 | **< 0.001** |
| Family category (Single-parent family) | 1.52 | 1.38–1.68 | **< 0.001** | 1.54 | 1.43–1.65 | **< 0.001** | 1.46 | 1.23–1.74 | **< 0.001** | 1.42 | 1.29–1.56 | **< 0.001** | 1.33 | 1.18–1.50 | **< 0.001** | 1.32 | 1.22–1.43 | **< 0.001** |
| Family category (Left-behind family) | 2.07 | 1.71–2.51 | **< 0.001** | 2.23 | 1.95–2.55 | **< 0.001** | 1.93 | 1.42–2.60 | **< 0.001** | 1.74 | 1.47–2.07 | **< 0.001** | 1.82 | 1.47–2.25 | **< 0.001** | 1.46 | 1.26–1.70 | **< 0.001** |
| Fathers education level (Primary school graduation) | 0.69 | 0.53–0.90 | **0.017** | 0.99 | 0.82–1.19 | 0.956 | 0.54 | 0.35–0.85 | **0.018** | 0.67 | 0.53–0.84 | **< 0.001** | 0.93 | 0.67–1.28 | 0.809 | 0.94 | 0.76–1.15 | 0.641 |
| Fathers education level (Junior high school graduation) | 0.62 | 0.47–0.81 | **0.002** | 0.85 | 0.71–1.03 | 0.143 | 0.44 | 0.28–0.69 | **< 0.001** | 0.57 | 0.45–0.72 | **< 0.001** | 0.79 | 0.57–1.10 | 0.305 | 0.87 | 0.71–1.07 | 0.298 |
| Fathers education level (High school/Technical school graduation) | 0.66 | 0.50–0.87 | **0.009** | 0.90 | 0.75–1.10 | 0.415 | 0.44 | 0.28–0.70 | **< 0.001** | 0.57 | 0.45–0.73 | **< 0.001** | 0.84 | 0.60–1.17 | 0.502 | 0.91 | 0.74–1.13 | 0.545 |
| Fathers education level (Associate degree graduation) | 0.74 | 0.56–0.99 | 0.089 | 1.04 | 0.86–1.27 | 0.772 | 0.52 | 0.32–0.84 | **0.018** | 0.61 | 0.48–0.79 | **< 0.001** | 0.87 | 0.61–1.23 | 0.639 | 0.92 | 0.74–1.14 | 0.557 |
| Fathers education level (Bachelor's degree graduation) | 0.70 | 0.52–0.94 | **0.041** | 1.08 | 0.89–1.33 | 0.570 | 0.55 | 0.33–0.89 | **0.034** | 0.68 | 0.53–0.89 | **0.007** | 0.92 | 0.65–1.32 | 0.809 | 0.92 | 0.73–1.15 | 0.559 |
| Fathers education level (Master's /Ph.D./ Postdoctoral graduation) | 0.77 | 0.51–1.17 | 0.392 | 0.99 | 0.75–1.31 | 0.956 | 0.41 | 0.20–0.86 | **0.038** | 0.92 | 0.64–1.32 | 0.670 | 1.52 | 0.96–2.40 | 0.147 | 0.99 | 0.72–1.35 | 0.961 |
| Fathers education level (Not sure) | 0.83 | 0.58–1.18 | 0.479 | 0.92 | 0.72–1.19 | 0.644 | 0.71 | 0.38–1.34 | 0.396 | 0.86 | 0.62–1.18 | 0.406 | 0.84 | 0.55–1.31 | 0.648 | 1.05 | 0.80–1.38 | 0.802 |
| Fathers education level (Others [father's role missing, such as the father passing away, etc.]) | 0.74 | 0.54–1.01 | 0.114 | 1.01 | 0.81–1.26 | 0.956 | 0.59 | 0.35–0.99 | 0.087 | 0.68 | 0.52–0.90 | **0.012** | 1.03 | 0.70–1.50 | 0.958 | 1.02 | 0.80–1.30 | 0.938 |
| Mothers education level (Primary school graduation) | 0.94 | 0.77–1.14 | 0.685 | 0.89 | 0.79–1.01 | 0.123 | 1.10 | 0.79–1.54 | 0.627 | 0.96 | 0.81–1.13 | 0.663 | 0.96 | 0.77–1.19 | 0.816 | 0.90 | 0.79–1.04 | 0.238 |
| Mothers education level (Junior high school graduation) | 0.85 | 0.69–1.03 | 0.179 | 0.80 | 0.70–0.90 | **< 0.001** | 0.82 | 0.59–1.16 | 0.381 | 0.86 | 0.72–1.01 | 0.107 | 0.85 | 0.68–1.05 | 0.263 | 0.81 | 0.71–0.93 | **0.005** |
| Mothers education level (High school/Technical school graduation) | 0.92 | 0.75–1.13 | 0.618 | 0.82 | 0.72–0.93 | **0.006** | 0.90 | 0.63–1.28 | 0.615 | 0.87 | 0.73–1.04 | 0.177 | 0.88 | 0.70–1.11 | 0.491 | 0.74 | 0.64–0.85 | **< 0.001** |
| Mothers education level (Associate degree graduation) | 1.06 | 0.85–1.32 | 0.744 | 0.88 | 0.76–1.01 | 0.123 | 0.87 | 0.59–1.28 | 0.557 | 0.90 | 0.74–1.09 | 0.338 | 1.01 | 0.79–1.30 | 0.959 | 0.80 | 0.69–0.94 | **0.013** |
| Mothers education level (Bachelor's degree graduation) | 1.07 | 0.85–1.36 | 0.710 | 0.77 | 0.67–0.90 | **0.002** | 0.93 | 0.61–1.40 | 0.733 | 0.84 | 0.68–1.03 | 0.119 | 0.93 | 0.72–1.21 | 0.770 | 0.77 | 0.66–0.91 | **0.005** |
| Mothers education level (Master's /Ph.D./ Postdoctoral graduation) | 1.24 | 0.83–1.84 | 0.479 | 0.99 | 0.76–1.29 | 0.956 | 1.55 | 0.81–2.98 | 0.311 | 0.80 | 0.55–1.15 | 0.284 | 1.02 | 0.66–1.57 | 0.959 | 0.88 | 0.65–1.18 | 0.545 |
| Mothers education level (Not sure) | 0.98 | 0.72–1.33 | 0.884 | 0.83 | 0.68–1.02 | 0.123 | 0.67 | 0.36–1.22 | 0.311 | 0.89 | 0.67–1.17 | 0.443 | 0.83 | 0.57–1.19 | 0.502 | 0.60 | 0.48–0.75 | **< 0.001** |
| Mothers education level (Others [mother's role missing, such as the mother passing away, etc.]) | 1.02 | 0.76–1.38 | 0.884 | 1.13 | 0.93–1.39 | 0.309 | 1.19 | 0.74–1.91 | 0.557 | 1.10 | 0.86–1.41 | 0.508 | 0.97 | 0.69–1.34 | 0.916 | 0.92 | 0.74–1.14 | 0.557 |
| Household income￥6000–14 000 | 1.01 | 0.94–1.08 | 0.865 | 1.05 | 1.01–1.10 | **0.049** | 0.84 | 0.74–0.96 | **0.020** | 0.88 | 0.82–0.93 | **< 0.001** | 0.90 | 0.83–0.98 | **0.028** | 0.94 | 0.89–0.98 | **0.020** |
| Household income￥14 000–23 000 | 1.01 | 0.93–1.11 | 0.817 | 1.10 | 1.04–1.16 | **< 0.001** | 0.93 | 0.80–1.08 | 0.428 | 0.83 | 0.77–0.90 | **< 0.001** | 0.83 | 0.76–0.92 | **0.001** | 0.87 | 0.81–0.92 | **< 0.001** |
| Household income￥23 000–36 000 | 0.98 | 0.88–1.09 | 0.757 | 1.08 | 1.01–1.15 | **0.039** | 0.82 | 0.67–0.99 | 0.082 | 0.81 | 0.73–0.89 | **< 0.001** | 0.94 | 0.84–1.06 | 0.502 | 0.87 | 0.81–0.94 | **< 0.001** |
| Household income￥36 000–70 000 | 1.04 | 0.93–1.18 | 0.668 | 1.09 | 1.01–1.18 | **0.045** | 0.87 | 0.69–1.09 | 0.350 | 0.75 | 0.67–0.83 | **< 0.001** | 0.81 | 0.70–0.93 | **0.008** | 0.90 | 0.83–0.98 | **0.033** |
| Household income (＞￥70,000) | 0.96 | 0.83–1.11 | 0.723 | 0.97 | 0.89–1.07 | 0.674 | 1.04 | 0.81–1.34 | 0.757 | 0.79 | 0.69–0.90 | **< 0.001** | 1.03 | 0.89–1.21 | 0.809 | 0.90 | 0.82–1.00 | 0.095 |
| Drinking (≤1 time/month) | 1.60 | 1.49–1.71 | **< 0.001** | 1.45 | 1.39–1.51 | **< 0.001** | 1.29 | 1.14–1.45 | **< 0.001** | 1.33 | 1.25–1.40 | **< 0.001** | 1.14 | 1.06–1.23 | **< 0.001** | 1.38 | 1.32–1.45 | **< 0.001** |
| Drinking 2–4 times/month | 2.59 | 2.36–2.85 | **< 0.001** | 1.79 | 1.68–1.90 | **< 0.001** | 2.08 | 1.76–2.46 | **< 0.001** | 1.96 | 1.80–2.13 | **< 0.001** | 1.66 | 1.49–1.85 | **< 0.001** | 1.90 | 1.78–2.03 | **< 0.001** |
| Drinking 2–3 times/week | 3.30 | 2.79–3.90 | **< 0.001** | 2.17 | 1.92–2.47 | **< 0.001** | 3.18 | 2.41–4.18 | **< 0.001** | 2.99 | 2.58–3.46 | **< 0.001** | 2.74 | 2.29–3.29 | **< 0.001** | 2.65 | 2.33–3.01 | **< 0.001** |
| Drinking (≥4 times/week) | 3.30 | 2.69–4.06 | **< 0.001** | 2.03 | 1.71–2.41 | **< 0.001** | 4.03 | 2.97–5.45 | **< 0.001** | 2.98 | 2.47–3.60 | **< 0.001** | 2.81 | 2.24–3.52 | **< 0.001** | 2.94 | 2.49–3.47 | **< 0.001** |
| Smoking (Yes) | 1.82 | 1.67–1.99 | **< 0.001** | 1.14 | 1.08–1.21 | **< 0.001** | 1.94 | 1.66–2.27 | **< 0.001** | 1.70 | 1.58–1.84 | **< 0.001** | 1.46 | 1.32–1.61 | **< 0.001** | 1.53 | 1.44–1.62 | **< 0.001** |
| Observations | 79 011 | | | 79 011 | | | 79 011 | | | 79 011 | | | 79 011 | | | 79 011 | | |
| R^2^/R^2^ adjusted | 0.052/0.052 | | | 0.035/0.035 | | | 0.050/0.049 | | | 0.042/0.041 | | | 0.032/0.032 | | | 0.027/0.026 | | |

Abbreviations: CI, confidence interval; FDR, false discovery rate; NSSI, non-suicidal self-injury; PTSD, post-traumatic stress disorder; SA, suicide attempt; SI, suicidal ideation.

| S Table 5. Exercise type, reference level: single anaerobics. | | | | | | | | | | | | | | | | | | |
| --- | --- | --- | --- | --- | --- | --- | --- | --- | --- | --- | --- | --- | --- | --- | --- | --- | --- | --- |
|  | **Lifetime NSSI** | | | **Lifetime SI** | | | **Lifetime SA** | | | **Depression** | | | **Anxiety** | | | **PTSD** | | |
| *Predictors* | *Odds ratios* | *CI (95%)* | *P value (fdr)* | *Odds ratios* | *CI (95%)* | *P value (fdr)* | *Odds ratios* | *CI (95%)* | *P value (fdr)* | *Odds ratios* | *CI (95%)* | *P value (fdr)* | *Odds ratios* | *CI (95%)* | *P valued (fdr)* | *Odds ratios* | *CI (95%)* | *P value (fdr)* |
| Exercise type (double swing) | 0.92 | 0.85–0.99 | 0.074 | 1.08 | 1.03–1.13 | **0.005** | 0.92 | 0.79–1.06 | 0.354 | 1.00 | 0.93–1.07 | 0.966 | 0.91 | 0.83–0.99 | 0.078 | 1.00 | 0.94–1.05 | 0.938 |
| Exercise type (low intensity aerobics) | 1.23 | 1.12–1.34 | **< 0.001** | 1.41 | 1.33–1.49 | **< 0.001** | 1.24 | 1.06–1.45 | **0.019** | 1.40 | 1.29–1.51 | **< 0.001** | 1.33 | 1.21–1.47 | **< 0.001** | 1.15 | 1.08–1.23 | **< 0.001** |
| Exercise type (medium intensity aerobics) | 1.10 | 1.01–1.20 | 0.074 | 1.26 | 1.20–1.33 | **< 0.001** | 1.14 | 0.98–1.33 | 0.157 | 1.12 | 1.04–1.21 | **0.007** | 1.12 | 1.02–1.23 | 0.051 | 1.07 | 1.01–1.14 | 0.051 |
| Exercise type (team ball) | 0.88 | 0.80–0.96 | **0.017** | 0.87 | 0.82–0.92 | **< 0.001** | 0.85 | 0.71–1.01 | 0.108 | 0.86 | 0.79–0.93 | **< 0.001** | 0.81 | 0.73–0.90 | **< 0.001** | 0.90 | 0.84–0.96 | **0.002** |
| Education level (Postgraduate) | 1.08 | 0.89–1.30 | 0.635 | 1.00 | 0.88–1.12 | 0.956 | 1.17 | 0.83–1.63 | 0.447 | 1.08 | 0.91–1.27 | 0.443 | 1.03 | 0.84–1.27 | 0.862 | 1.00 | 0.88–1.15 | 0.966 |
| Education level (Doctoral candidate) | 0.85 | 0.41–1.78 | 0.757 | 0.82 | 0.49–1.37 | 0.579 | 1.78 | 0.60–5.29 | 0.385 | 0.36 | 0.14–0.92 | 0.050 | 0.35 | 0.12–1.00 | 0.105 | 1.10 | 0.65–1.86 | 0.799 |
| Age | 1.00 | 0.98–1.02 | 0.817 | 0.99 | 0.98–1.00 | 0.319 | 0.98 | 0.94–1.02 | 0.385 | 0.99 | 0.97–1.01 | 0.238 | 1.00 | 0.98–1.02 | 0.977 | 0.99 | 0.98–1.01 | 0.346 |
| BMI | 0.99 | 0.99–1.00 | **0.023** | 1.00 | 0.99–1.00 | 0.053 | 0.99 | 0.98–1.00 | **0.019** | 1.00 | 0.99–1.00 | 0.119 | 1.00 | 0.99–1.01 | 0.862 | 1.00 | 1.00–1.00 | 0.718 |
| Gender (Female) | 1.73 | 1.61–1.85 | **< 0.001** | 1.66 | 1.59–1.72 | **< 0.001** | 2.03 | 1.78–2.31 | **< 0.001** | 1.19 | 1.12–1.26 | **< 0.001** | 1.21 | 1.12–1.30 | **< 0.001** | 1.26 | 1.20–1.31 | **< 0.001** |
| Gender identification (Transgender) | 2.39 | 2.01–2.83 | **< 0.001** | 2.01 | 1.77–2.28 | **< 0.001** | 1.88 | 1.41–2.52 | **< 0.001** | 2.40 | 2.06–2.80 | **< 0.001** | 2.74 | 2.29–3.28 | **< 0.001** | 1.84 | 1.61–2.12 | **< 0.001** |
| Gender identification (Nonbinary or genderquee) | 3.91 | 3.41–4.47 | **< 0.001** | 3.28 | 2.92–3.68 | **< 0.001** | 3.64 | 2.93–4.51 | **< 0.001** | 2.98 | 2.61–3.40 | **< 0.001** | 3.03 | 2.60–3.54 | **< 0.001** | 2.45 | 2.17–2.76 | **< 0.001** |
| Gender identification (Uncertainty) | 2.04 | 1.71–2.44 | **< 0.001** | 1.74 | 1.52–1.98 | **< 0.001** | 1.98 | 1.46–2.69 | **< 0.001** | 2.17 | 1.86–2.54 | **< 0.001** | 1.92 | 1.58–2.34 | **< 0.001** | 1.90 | 1.66–2.17 | **< 0.001** |
| Gender identification (Questioning) | 1.08 | 0.94–1.24 | 0.463 | 0.87 | 0.80–0.95 | **0.006** | 1.15 | 0.91–1.45 | 0.354 | 1.20 | 1.07–1.34 | **0.003** | 1.08 | 0.93–1.26 | 0.502 | 1.09 | 0.99–1.20 | 0.108 |
| Ethnicity (Han) | 1.03 | 0.94–1.13 | 0.701 | 1.02 | 0.96–1.08 | 0.623 | 0.96 | 0.82–1.12 | 0.629 | 0.95 | 0.88–1.03 | 0.312 | 0.97 | 0.88–1.07 | 0.767 | 0.98 | 0.92–1.05 | 0.740 |
| Family category (Extended family [three generations or more]) | 0.98 | 0.91–1.06 | 0.744 | 1.00 | 0.96–1.05 | 0.956 | 0.97 | 0.84–1.12 | 0.733 | 0.98 | 0.92–1.05 | 0.670 | 1.03 | 0.94–1.12 | 0.767 | 1.05 | 1.00–1.11 | 0.092 |
| Family category (Foster/Adoptive family) | 1.81 | 1.04–3.15 | 0.074 | 1.79 | 1.17–2.74 | **0.015** | 1.86 | 0.79–4.38 | 0.262 | 2.12 | 1.37–3.27 | **0.001** | 2.26 | 1.35–3.78 | **0.005** | 1.33 | 0.83–2.13 | 0.346 |
| Family category (Blended family) | 1.72 | 1.50–1.97 | **< 0.001** | 1.71 | 1.55–1.89 | **< 0.001** | 1.89 | 1.51–2.36 | **< 0.001** | 1.70 | 1.49–1.92 | **< 0.001** | 1.58 | 1.35–1.86 | **< 0.001** | 1.52 | 1.37–1.70 | **< 0.001** |
| Family category (Single-parent family) | 1.52 | 1.38–1.68 | **< 0.001** | 1.54 | 1.43–1.65 | **< 0.001** | 1.46 | 1.23–1.74 | **< 0.001** | 1.42 | 1.29–1.56 | **< 0.001** | 1.33 | 1.18–1.50 | **< 0.001** | 1.32 | 1.22–1.43 | **< 0.001** |
| Family category (Left-behind family) | 2.07 | 1.71–2.51 | **< 0.001** | 2.23 | 1.95–2.55 | **< 0.001** | 1.93 | 1.42–2.60 | **< 0.001** | 1.74 | 1.47–2.07 | **< 0.001** | 1.82 | 1.47–2.25 | **< 0.001** | 1.46 | 1.26–1.70 | **< 0.001** |
| Fathers education level (Primary school graduation) | 0.69 | 0.53–0.90 | **0.017** | 0.99 | 0.82–1.19 | 0.956 | 0.54 | 0.35–0.85 | **0.019** | 0.67 | 0.53–0.84 | **< 0.001** | 0.93 | 0.67–1.28 | 0.809 | 0.94 | 0.76–1.15 | 0.641 |
| Fathers education level (Junior high school graduation) | 0.62 | 0.47–0.81 | **0.002** | 0.85 | 0.71–1.03 | 0.143 | 0.44 | 0.28–0.69 | **< 0.001** | 0.57 | 0.45–0.72 | **< 0.001** | 0.79 | 0.57–1.10 | 0.305 | 0.87 | 0.71–1.07 | 0.298 |
| Fathers education level (High school/Technical school graduation) | 0.66 | 0.50–0.87 | **0.009** | 0.90 | 0.75–1.10 | 0.415 | 0.44 | 0.28–0.70 | **< 0.001** | 0.57 | 0.45–0.73 | **< 0.001** | 0.84 | 0.60–1.17 | 0.502 | 0.91 | 0.74–1.13 | 0.545 |
| Fathers education level (Associate degree graduation) | 0.74 | 0.56–0.99 | 0.085 | 1.04 | 0.86–1.27 | 0.772 | 0.52 | 0.32–0.84 | **0.019** | 0.61 | 0.48–0.79 | **< 0.001** | 0.87 | 0.61–1.23 | 0.639 | 0.92 | 0.74–1.14 | 0.557 |
| Fathers education level (Bachelor's degree graduation) | 0.70 | 0.52–0.94 | **0.041** | 1.08 | 0.89–1.33 | 0.570 | 0.55 | 0.33–0.89 | **0.036** | 0.68 | 0.53–0.89 | **0.007** | 0.92 | 0.65–1.32 | 0.809 | 0.92 | 0.73–1.15 | 0.559 |
| Fathers education level (Master's/Ph.D./ Postdoctoral graduation) | 0.77 | 0.51–1.17 | 0.377 | 0.99 | 0.75–1.31 | 0.956 | 0.41 | 0.20–0.86 | **0.040** | 0.92 | 0.64–1.32 | 0.670 | 1.52 | 0.96–2.40 | 0.147 | 0.99 | 0.72–1.35 | 0.961 |
| Fathers education level (Not sure) | 0.83 | 0.58–1.18 | 0.463 | 0.92 | 0.72–1.19 | 0.644 | 0.71 | 0.38–1.34 | 0.385 | 0.86 | 0.62–1.18 | 0.406 | 0.84 | 0.55–1.31 | 0.648 | 1.05 | 0.80–1.38 | 0.802 |
| Fathers education level (Others [father's role missing, such as father passing away, etc.]) | 0.74 | 0.54–1.01 | 0.109 | 1.01 | 0.81–1.26 | 0.956 | 0.59 | 0.35–0.99 | 0.090 | 0.68 | 0.52–0.90 | **0.012** | 1.03 | 0.70–1.50 | 0.958 | 1.02 | 0.80–1.30 | 0.938 |
| Mothers education level (Primary school graduation) | 0.94 | 0.77–1.14 | 0.685 | 0.89 | 0.79–1.01 | 0.123 | 1.10 | 0.79–1.54 | 0.627 | 0.96 | 0.81–1.13 | 0.663 | 0.96 | 0.77–1.19 | 0.816 | 0.90 | 0.79–1.04 | 0.238 |
| Mothers education level (Junior high school graduation) | 0.85 | 0.69–1.03 | 0.172 | 0.80 | 0.70–0.90 | **< 0.001** | 0.82 | 0.59–1.16 | 0.370 | 0.86 | 0.72–1.01 | 0.107 | 0.85 | 0.68–1.05 | 0.263 | 0.81 | 0.71–0.93 | **0.005** |
| Mothers education level (High school/Technical school graduation) | 0.92 | 0.75–1.13 | 0.618 | 0.82 | 0.72–0.93 | **0.006** | 0.90 | 0.63–1.28 | 0.615 | 0.87 | 0.73–1.04 | 0.177 | 0.88 | 0.70–1.11 | 0.491 | 0.74 | 0.64–0.85 | **< 0.001** |
| Mothers education level (Associate degree graduation) | 1.06 | 0.85–1.32 | 0.744 | 0.88 | 0.76–1.01 | 0.123 | 0.87 | 0.59–1.28 | 0.557 | 0.90 | 0.74–1.09 | 0.338 | 1.01 | 0.79–1.30 | 0.959 | 0.80 | 0.69–0.94 | **0.013** |
| Mothers education level (Bachelor's degree graduation) | 1.07 | 0.85–1.36 | 0.710 | 0.77 | 0.67–0.90 | **0.002** | 0.93 | 0.61–1.40 | 0.733 | 0.84 | 0.68–1.03 | 0.119 | 0.93 | 0.72–1.21 | 0.770 | 0.77 | 0.66–0.91 | **0.005** |
| Mothers education level (Master's/Ph.D./ Postdoctoral graduation) | 1.24 | 0.83–1.84 | 0.463 | 0.99 | 0.76–1.29 | 0.956 | 1.55 | 0.81–2.98 | 0.301 | 0.80 | 0.55–1.15 | 0.284 | 1.02 | 0.66–1.57 | 0.959 | 0.88 | 0.65–1.18 | 0.545 |
| Mothers education level (Not sure) | 0.98 | 0.72–1.33 | 0.884 | 0.83 | 0.68–1.02 | 0.123 | 0.67 | 0.36–1.22 | 0.301 | 0.89 | 0.67–1.17 | 0.443 | 0.83 | 0.57–1.19 | 0.502 | 0.60 | 0.48–0.75 | **< 0.001** |
| Mothers education level (Others [mother's role missing, such as the mother passing away, etc.]) | 1.02 | 0.76–1.38 | 0.884 | 1.13 | 0.93–1.39 | 0.309 | 1.19 | 0.74–1.91 | 0.557 | 1.10 | 0.86–1.41 | 0.508 | 0.97 | 0.69–1.34 | 0.916 | 0.92 | 0.74–1.14 | 0.557 |
| Household income￥6000–14 000 | 1.01 | 0.94–1.08 | 0.865 | 1.05 | 1.01–1.10 | **0.049** | 0.84 | 0.74–0.96 | **0.021** | 0.88 | 0.82–0.93 | **< 0.001** | 0.90 | 0.83–0.98 | **0.028** | 0.94 | 0.89–0.98 | **0.020** |
| Household income￥14 000–23 000 | 1.01 | 0.93–1.11 | 0.817 | 1.10 | 1.04–1.16 | **< 0.001** | 0.93 | 0.80–1.08 | 0.416 | 0.83 | 0.77–0.90 | **< 0.001** | 0.83 | 0.76–0.92 | **< 0.001** | 0.87 | 0.81–0.92 | **< 0.001** |
| Household income￥23 000–36 000 | 0.98 | 0.88–1.09 | 0.757 | 1.08 | 1.01–1.15 | **0.039** | 0.82 | 0.67–0.99 | 0.086 | 0.81 | 0.73–0.89 | **< 0.001** | 0.94 | 0.84–1.06 | 0.502 | 0.87 | 0.81–0.94 | **< 0.001** |
| Household income￥36 000–70 000 | 1.04 | 0.93–1.18 | 0.668 | 1.09 | 1.01–1.18 | **0.045** | 0.87 | 0.69–1.09 | 0.339 | 0.75 | 0.67–0.83 | **< 0.001** | 0.81 | 0.70–0.93 | **0.008** | 0.90 | 0.83–0.98 | **0.034** |
| Household income (＞￥70,000) | 0.96 | 0.83–1.11 | 0.723 | 0.97 | 0.89–1.07 | 0.674 | 1.04 | 0.81–1.34 | 0.757 | 0.79 | 0.69–0.90 | **< 0.001** | 1.03 | 0.89–1.21 | 0.809 | 0.90 | 0.82–1.00 | 0.095 |
| Drinking (≤1 time/month) | 1.60 | 1.49–1.71 | **< 0.001** | 1.45 | 1.39–1.51 | **< 0.001** | 1.29 | 1.14–1.45 | **< 0.001** | 1.33 | 1.25–1.40 | **< 0.001** | 1.14 | 1.06–1.23 | **< 0.001** | 1.38 | 1.32–1.45 | **< 0.001** |
| Drinking 2–4 times/month | 2.59 | 2.36–2.85 | **< 0.001** | 1.79 | 1.68–1.90 | **< 0.001** | 2.08 | 1.76–2.46 | **< 0.001** | 1.96 | 1.80–2.13 | **< 0.001** | 1.66 | 1.49–1.85 | **< 0.001** | 1.90 | 1.78–2.03 | **< 0.001** |
| Drinking 2–3 times/week | 3.30 | 2.79–3.90 | **< 0.001** | 2.17 | 1.92–2.47 | **< 0.001** | 3.18 | 2.41–4.18 | **< 0.001** | 2.99 | 2.58–3.46 | **< 0.001** | 2.74 | 2.29–3.29 | **< 0.001** | 2.65 | 2.33–3.01 | **< 0.001** |
| Drinking (≥ 4 times/week) | 3.30 | 2.69–4.06 | **< 0.001** | 2.03 | 1.71–2.41 | **< 0.001** | 4.03 | 2.97–5.45 | **< 0.001** | 2.98 | 2.47–3.60 | **< 0.001** | 2.81 | 2.24–3.52 | **< 0.001** | 2.94 | 2.49–3.47 | **< 0.001** |
| Smoking (Yes) | 1.82 | 1.67–1.99 | **< 0.001** | 1.14 | 1.08–1.21 | **< 0.001** | 1.94 | 1.66–2.27 | **< 0.001** | 1.70 | 1.58–1.84 | **< 0.001** | 1.46 | 1.32–1.61 | **< 0.001** | 1.53 | 1.44–1.62 | **< 0.001** |
| Observations | 79 011 | | | 79 011 | | | 79 011 | | | 79 011 | | | 79 011 | | | 79 011 | | |
| R^2^/R^2^ adjusted | 0.052/0.052 | | | 0.035/0.035 | | | 0.050/0.049 | | | 0.042/0.041 | | | 0.032/0.032 | | | 0.027/0.026 | | |

Abbreviations: CI, confidence interval; FDR, false discovery rate; NSSI, non-suicidal self-injury; PTSD, post-traumatic stress disorder; SA, suicide attempt; SI, suicidal ideation.

| S Table 6. Exercise type, reference level: medium intensity aerobics. | | | | | | | | | | | | | | | | | | |
| --- | --- | --- | --- | --- | --- | --- | --- | --- | --- | --- | --- | --- | --- | --- | --- | --- | --- | --- |
|  | **Lifetime NSSI** | | | **Lifetime SI** | | | **Lifetime SA** | | | **Depression** | | | **Anxiety** | | | **PTSD** | | |
| *Predictors* | *Odds ratios* | *CI (95%)* | *P value (fdr)* | *Odds ratios* | *CI (95%)* | *P value (fdr)* | *Odds ratios* | *CI (95%)* | *P value (fdr)* | *Odds ratios* | *CI (95%)* | *P value (fdr)* | *Odds ratios* | *CI (95%)* | *P valued (fdr)* | *Odds ratios* | *CI (95%)* | *P value (fdr)* |
| Exercise type (double swing) | 0.84 | 0.77–0.91 | **< 0.001** | 0.86 | 0.81–0.90 | **< 0.001** | 0.80 | 0.70–0.93 | **0.009** | 0.89 | 0.83–0.96 | **0.004** | 0.81 | 0.74–0.89 | **< 0.001** | 0.93 | 0.88–0.99 | **0.030** |
| Exercise type (low intensity aerobics) | 1.12 | 1.02–1.22 | **0.034** | 1.12 | 1.05–1.18 | **< 0.001** | 1.09 | 0.93–1.27 | 0.385 | 1.25 | 1.15–1.35 | **< 0.001** | 1.19 | 1.08–1.31 | **0.002** | 1.08 | 1.01–1.15 | **0.042** |
| Exercise type (single anaerobics) | 0.91 | 0.84–0.99 | 0.073 | 0.79 | 0.75–0.84 | **< 0.001** | 0.88 | 0.75–1.02 | 0.157 | 0.89 | 0.83–0.97 | **0.007** | 0.89 | 0.81–0.98 | **0.048** | 0.93 | 0.88–0.99 | **0.049** |
| Exercise type (team ball) | 0.80 | 0.73–0.88 | **< 0.001** | 0.69 | 0.65–0.73 | **< 0.001** | 0.74 | 0.62–0.88 | **0.002** | 0.77 | 0.70–0.83 | **< 0.001** | 0.72 | 0.65–0.81 | **< 0.001** | 0.84 | 0.78–0.90 | **< 0.001** |
| Education level (Postgraduate) | 1.08 | 0.89–1.30 | 0.635 | 1.00 | 0.88–1.12 | 0.956 | 1.17 | 0.83–1.63 | 0.447 | 1.08 | 0.91–1.27 | 0.432 | 1.03 | 0.84–1.27 | 0.862 | 1.00 | 0.88–1.15 | 0.966 |
| Education level (Doctoral candidate) | 0.85 | 0.41–1.78 | 0.757 | 0.82 | 0.49–1.37 | 0.579 | 1.78 | 0.60–5.29 | 0.385 | 0.36 | 0.14–0.92 | **0.049** | 0.35 | 0.12–1.00 | 0.105 | 1.10 | 0.65–1.86 | 0.780 |
| Age | 1.00 | 0.98–1.02 | 0.817 | 0.99 | 0.98–1.00 | 0.319 | 0.98 | 0.94–1.02 | 0.385 | 0.99 | 0.97–1.01 | 0.232 | 1.00 | 0.98–1.02 | 0.977 | 0.99 | 0.98–1.01 | 0.336 |
| BMI | 0.99 | 0.99–1.00 | **0.023** | 1.00 | 0.99–1.00 | 0.053 | 0.99 | 0.98–1.00 | **0.018** | 1.00 | 0.99–1.00 | 0.116 | 1.00 | 0.99–1.01 | 0.862 | 1.00 | 1.00–1.00 | 0.700 |
| Gender (Female) | 1.73 | 1.61–1.85 | **< 0.001** | 1.66 | 1.59–1.72 | **< 0.001** | 2.03 | 1.78–2.31 | **< 0.001** | 1.19 | 1.12–1.26 | **< 0.001** | 1.21 | 1.12–1.30 | **< 0.001** | 1.26 | 1.20–1.31 | **< 0.001** |
| Gender identification (Transgender) | 2.39 | 2.01–2.83 | **< 0.001** | 2.01 | 1.77–2.28 | **< 0.001** | 1.88 | 1.41–2.52 | **< 0.001** | 2.40 | 2.06–2.80 | **< 0.001** | 2.74 | 2.29–3.28 | **< 0.001** | 1.84 | 1.61–2.12 | **< 0.001** |
| Gender identification (Nonbinary or genderquee) | 3.91 | 3.41–4.47 | **< 0.001** | 3.28 | 2.92–3.68 | **< 0.001** | 3.64 | 2.93–4.51 | **< 0.001** | 2.98 | 2.61–3.40 | **< 0.001** | 3.03 | 2.60–3.54 | **< 0.001** | 2.45 | 2.17–2.76 | **< 0.001** |
| Gender identification (Uncertainty) | 2.04 | 1.71–2.44 | **< 0.001** | 1.74 | 1.52–1.98 | **< 0.001** | 1.98 | 1.46–2.69 | **< 0.001** | 2.17 | 1.86–2.54 | **< 0.001** | 1.92 | 1.58–2.34 | **< 0.001** | 1.90 | 1.66–2.17 | **< 0.001** |
| Gender identification (Questioning) | 1.08 | 0.94–1.24 | 0.463 | 0.87 | 0.80–0.95 | **0.006** | 1.15 | 0.91–1.45 | 0.366 | 1.20 | 1.07–1.34 | **0.003** | 1.08 | 0.93–1.26 | 0.502 | 1.09 | 0.99–1.20 | 0.104 |
| Ethnicity (Han) | 1.03 | 0.94–1.13 | 0.701 | 1.02 | 0.96–1.08 | 0.623 | 0.96 | 0.82–1.12 | 0.629 | 0.95 | 0.88–1.03 | 0.304 | 0.97 | 0.88–1.07 | 0.767 | 0.98 | 0.92–1.05 | 0.722 |
| Family category (Extended family [three generations or more]) | 0.98 | 0.91–1.06 | 0.744 | 1.00 | 0.96–1.05 | 0.956 | 0.97 | 0.84–1.12 | 0.733 | 0.98 | 0.92–1.05 | 0.655 | 1.03 | 0.94–1.12 | 0.767 | 1.05 | 1.00–1.11 | 0.088 |
| Family category (Foster/Adoptive family) | 1.81 | 1.04–3.15 | 0.074 | 1.79 | 1.17–2.74 | **0.015** | 1.86 | 0.79–4.38 | 0.262 | 2.12 | 1.37–3.27 | **< 0.001** | 2.26 | 1.35–3.78 | **0.005** | 1.33 | 0.83–2.13 | 0.336 |
| Family category (Blended family) | 1.72 | 1.50–1.97 | **< 0.001** | 1.71 | 1.55–1.89 | **< 0.001** | 1.89 | 1.51–2.36 | **< 0.001** | 1.70 | 1.49–1.92 | **< 0.001** | 1.58 | 1.35–1.86 | **< 0.001** | 1.52 | 1.37–1.70 | **< 0.001** |
| Family category (Single-parent family) | 1.52 | 1.38–1.68 | **< 0.001** | 1.54 | 1.43–1.65 | **< 0.001** | 1.46 | 1.23–1.74 | **< 0.001** | 1.42 | 1.29–1.56 | **< 0.001** | 1.33 | 1.18–1.50 | **< 0.001** | 1.32 | 1.22–1.43 | **< 0.001** |
| Family category (Left-behind family) | 2.07 | 1.71–2.51 | **< 0.001** | 2.23 | 1.95–2.55 | **< 0.001** | 1.93 | 1.42–2.60 | **< 0.001** | 1.74 | 1.47–2.07 | **< 0.001** | 1.82 | 1.47–2.25 | **< 0.001** | 1.46 | 1.26–1.70 | **< 0.001** |
| Fathers education level (Primary school graduation) | 0.69 | 0.53–0.90 | **0.017** | 0.99 | 0.82–1.19 | 0.956 | 0.54 | 0.35–0.85 | **0.018** | 0.67 | 0.53–0.84 | **< 0.001** | 0.93 | 0.67–1.28 | 0.809 | 0.94 | 0.76–1.15 | 0.625 |
| Fathers education level (Junior high school graduation) | 0.62 | 0.47–0.81 | **0.002** | 0.85 | 0.71–1.03 | 0.143 | 0.44 | 0.28–0.69 | **< 0.001** | 0.57 | 0.45–0.72 | **< 0.001** | 0.79 | 0.57–1.10 | 0.305 | 0.87 | 0.71–1.07 | 0.288 |
| Fathers education level (High school/Technical school graduation) | 0.66 | 0.50–0.87 | **0.009** | 0.90 | 0.75–1.10 | 0.415 | 0.44 | 0.28–0.70 | **< 0.001** | 0.57 | 0.45–0.73 | **< 0.001** | 0.84 | 0.60–1.17 | 0.502 | 0.91 | 0.74–1.13 | 0.529 |
| Fathers education level (Associate degree graduation) | 0.74 | 0.56–0.99 | 0.085 | 1.04 | 0.86–1.27 | 0.772 | 0.52 | 0.32–0.84 | **0.018** | 0.61 | 0.48–0.79 | **< 0.001** | 0.87 | 0.61–1.23 | 0.639 | 0.92 | 0.74–1.14 | 0.542 |
| Fathers education level (Bachelor's degree graduation) | 0.70 | 0.52–0.94 | **0.039** | 1.08 | 0.89–1.33 | 0.570 | 0.55 | 0.33–0.89 | **0.034** | 0.68 | 0.53–0.89 | **0.007** | 0.92 | 0.65–1.32 | 0.809 | 0.92 | 0.73–1.15 | 0.544 |
| Fathers education level (Master's/Ph.D./ Postdoctoral graduation) | 0.77 | 0.51–1.17 | 0.377 | 0.99 | 0.75–1.31 | 0.956 | 0.41 | 0.20–0.86 | **0.038** | 0.92 | 0.64–1.32 | 0.655 | 1.52 | 0.96–2.40 | 0.147 | 0.99 | 0.72–1.35 | 0.961 |
| Fathers education level (Not sure) | 0.83 | 0.58–1.18 | 0.463 | 0.92 | 0.72–1.19 | 0.644 | 0.71 | 0.38–1.34 | 0.385 | 0.86 | 0.62–1.18 | 0.396 | 0.84 | 0.55–1.31 | 0.648 | 1.05 | 0.80–1.38 | 0.783 |
| Fathers education level (Others [father's role missing, such as the father passing away, etc.]) | 0.74 | 0.54–1.01 | 0.109 | 1.01 | 0.81–1.26 | 0.956 | 0.59 | 0.35–0.99 | 0.087 | 0.68 | 0.52–0.90 | **0.011** | 1.03 | 0.70–1.50 | 0.958 | 1.02 | 0.80–1.30 | 0.938 |
| Mothers education level (Primary school graduation) | 0.94 | 0.77–1.14 | 0.685 | 0.89 | 0.79–1.01 | 0.123 | 1.10 | 0.79–1.54 | 0.627 | 0.96 | 0.81–1.13 | 0.648 | 0.96 | 0.77–1.19 | 0.816 | 0.90 | 0.79–1.04 | 0.230 |
| Mothers education level (Junior high school graduation) | 0.85 | 0.69–1.03 | 0.172 | 0.80 | 0.70–0.90 | **< 0.001** | 0.82 | 0.59–1.16 | 0.381 | 0.86 | 0.72–1.01 | 0.104 | 0.85 | 0.68–1.05 | 0.263 | 0.81 | 0.71–0.93 | **0.006** |
| Mothers education level (High school/Technical school graduation) | 0.92 | 0.75–1.13 | 0.618 | 0.82 | 0.72–0.93 | **0.006** | 0.90 | 0.63–1.28 | 0.615 | 0.87 | 0.73–1.04 | 0.172 | 0.88 | 0.70–1.11 | 0.491 | 0.74 | 0.64–0.85 | **< 0.001** |
| Mothers education level (Associate degree graduation) | 1.06 | 0.85–1.32 | 0.744 | 0.88 | 0.76–1.01 | 0.123 | 0.87 | 0.59–1.28 | 0.557 | 0.90 | 0.74–1.09 | 0.329 | 1.01 | 0.79–1.30 | 0.959 | 0.80 | 0.69–0.94 | **0.014** |
| Mothers education level (Bachelor's degree graduation) | 1.07 | 0.85–1.36 | 0.710 | 0.77 | 0.67–0.90 | **0.002** | 0.93 | 0.61–1.40 | 0.733 | 0.84 | 0.68–1.03 | 0.116 | 0.93 | 0.72–1.21 | 0.770 | 0.77 | 0.66–0.91 | **0.006** |
| Mothers education level (Master's/Ph.D./ Postdoctoral graduation) | 1.24 | 0.83–1.84 | 0.463 | 0.99 | 0.76–1.29 | 0.956 | 1.55 | 0.81–2.98 | 0.301 | 0.80 | 0.55–1.15 | 0.276 | 1.02 | 0.66–1.57 | 0.959 | 0.88 | 0.65–1.18 | 0.529 |
| Mothers education level (Not sure) | 0.98 | 0.72–1.33 | 0.884 | 0.83 | 0.68–1.02 | 0.123 | 0.67 | 0.36–1.22 | 0.301 | 0.89 | 0.67–1.17 | 0.432 | 0.83 | 0.57–1.19 | 0.502 | 0.60 | 0.48–0.75 | **< 0.001** |
| Mothers education level (Others [mother's role missing, such as the mother passing away, etc.]) | 1.02 | 0.76–1.38 | 0.884 | 1.13 | 0.93–1.39 | 0.309 | 1.19 | 0.74–1.91 | 0.557 | 1.10 | 0.86–1.41 | 0.496 | 0.97 | 0.69–1.34 | 0.916 | 0.92 | 0.74–1.14 | 0.542 |
| Household income￥6000–14 000 | 1.01 | 0.94–1.08 | 0.865 | 1.05 | 1.01–1.10 | **0.049** | 0.84 | 0.74–0.96 | **0.020** | 0.88 | 0.82–0.93 | **< 0.001** | 0.90 | 0.83–0.98 | **0.027** | 0.94 | 0.89–0.98 | **0.021** |
| Household income￥14 000–23 000 | 1.01 | 0.93–1.11 | 0.817 | 1.10 | 1.04–1.16 | **< 0.001** | 0.93 | 0.80–1.08 | 0.416 | 0.83 | 0.77–0.90 | **< 0.001** | 0.83 | 0.76–0.92 | **0.001** | 0.87 | 0.81–0.92 | **< 0.001** |
| Household income￥23 000–36 000 | 0.98 | 0.88–1.09 | 0.757 | 1.08 | 1.01–1.15 | **0.039** | 0.82 | 0.67–0.99 | 0.082 | 0.81 | 0.73–0.89 | **< 0.001** | 0.94 | 0.84–1.06 | 0.502 | 0.87 | 0.81–0.94 | **< 0.001** |
| Household income￥36 000–70 000 | 1.04 | 0.93–1.18 | 0.668 | 1.09 | 1.01–1.18 | **0.045** | 0.87 | 0.69–1.09 | 0.339 | 0.75 | 0.67–0.83 | **< 0.001** | 0.81 | 0.70–0.93 | **0.007** | 0.90 | 0.83–0.98 | **0.034** |
| Household income (＞￥70,000) | 0.96 | 0.83–1.11 | 0.723 | 0.97 | 0.89–1.07 | 0.674 | 1.04 | 0.81–1.34 | 0.757 | 0.79 | 0.69–0.90 | **< 0.001** | 1.03 | 0.89–1.21 | 0.809 | 0.90 | 0.82–1.00 | 0.092 |
| Drinking (≤1 time/month) | 1.60 | 1.49–1.71 | **< 0.001** | 1.45 | 1.39–1.51 | **< 0.001** | 1.29 | 1.14–1.45 | **< 0.001** | 1.33 | 1.25–1.40 | **< 0.001** | 1.14 | 1.06–1.23 | **< 0.001** | 1.38 | 1.32–1.45 | **< 0.001** |
| Drinking 2–4 times/month | 2.59 | 2.36–2.85 | **< 0.001** | 1.79 | 1.68–1.90 | **< 0.001** | 2.08 | 1.76–2.46 | **< 0.001** | 1.96 | 1.80–2.13 | **< 0.001** | 1.66 | 1.49–1.85 | **< 0.001** | 1.90 | 1.78–2.03 | **< 0.001** |
| Drinking 2–3 times/week | 3.30 | 2.79–3.90 | **< 0.001** | 2.17 | 1.92–2.47 | **< 0.001** | 3.18 | 2.41–4.18 | **< 0.001** | 2.99 | 2.58–3.46 | **< 0.001** | 2.74 | 2.29–3.29 | **< 0.001** | 2.65 | 2.33–3.01 | **< 0.001** |
| Drinking (≥4 times/week) | 3.30 | 2.69–4.06 | **< 0.001** | 2.03 | 1.71–2.41 | **< 0.001** | 4.03 | 2.97–5.45 | **< 0.001** | 2.98 | 2.47–3.60 | **< 0.001** | 2.81 | 2.24–3.52 | **< 0.001** | 2.94 | 2.49–3.47 | **< 0.001** |
| Smoking (Yes) | 1.82 | 1.67–1.99 | **< 0.001** | 1.14 | 1.08–1.21 | **< 0.001** | 1.94 | 1.66–2.27 | **< 0.001** | 1.70 | 1.58–1.84 | **< 0.001** | 1.46 | 1.32–1.61 | **< 0.001** | 1.53 | 1.44–1.62 | **< 0.001** |
| Observations | 79 011 | | | 79 011 | | | 79 011 | | | 79 011 | | | 79 011 | | | 79 011 | | |
| R^2^/R^2^ adjusted | 0.052/0.052 | | | 0.035/0.035 | | | 0.050/0.049 | | | 0.042/0.041 | | | 0.032/0.032 | | | 0.027/0.026 | | |

Abbreviations: CI, confidence interval; FDR, false discovery rate; NSSI, non-suicidal self-injury; PTSD, post-traumatic stress disorder; SA, suicide attempt; SI, suicidal ideation.

| S Table 7. Exercise type, reference level: low intensity aerobics. | | | | | | | | | | | | | | | | | | |
| --- | --- | --- | --- | --- | --- | --- | --- | --- | --- | --- | --- | --- | --- | --- | --- | --- | --- | --- |
|  | **Lifetime NSSI** | | | **Lifetime SI** | | | **Lifetime SA** | | | **Depression** | | | **Anxiety** | | | **PTSD** | | |
| *Predictors* | *Odds ratios* | *CI (95%)* | *P value (fdr)* | *Odds ratios* | *CI (95%)* | *P value (fdr)* | *Odds ratios* | *CI (95%)* | *P value (fdr)* | *Odds ratios* | *CI (95%)* | *P value (fdr)* | *Odds ratios* | *CI (95%)* | *P value (fdr)* | *Odds ratios* | *CI (95%)* | *P value (fdr)* |
| Exercise type (double swing) | 0.75 | 0.69–0.81 | **< 0.001** | 0.77 | 0.73–0.81 | **< 0.001** | 0.74 | 0.64–0.86 | **< 0.001** | 0.72 | 0.66–0.77 | **< 0.001** | 0.68 | 0.62–0.75 | **< 0.001** | 0.86 | 0.81–0.92 | **< 0.001** |
| Exercise type (medium intensity aerobics) | 0.89 | 0.82–0.98 | **0.032** | 0.90 | 0.85–0.95 | **< 0.001** | 0.92 | 0.79–1.08 | 0.385 | 0.80 | 0.74–0.87 | **< 0.001** | 0.84 | 0.76–0.93 | **0.002** | 0.93 | 0.87–0.99 | **0.040** |
| Exercise type (single anaerobics) | 0.81 | 0.75–0.89 | **< 0.001** | 0.71 | 0.67–0.75 | **< 0.001** | 0.81 | 0.69–0.94 | **0.017** | 0.72 | 0.66–0.77 | **< 0.001** | 0.75 | 0.68–0.83 | **< 0.001** | 0.87 | 0.81–0.92 | **< 0.001** |
| Exercise type (team ball) | 0.71 | 0.65–0.79 | **< 0.001** | 0.62 | 0.58–0.66 | **< 0.001** | 0.68 | 0.57–0.82 | **< 0.001** | 0.61 | 0.56–0.67 | **< 0.001** | 0.61 | 0.54–0.68 | **< 0.001** | 0.78 | 0.72–0.83 | **< 0.001** |
| Education level (Postgraduate) | 1.08 | 0.89–1.30 | 0.635 | 1.00 | 0.88–1.12 | 0.956 | 1.17 | 0.83–1.63 | 0.447 | 1.08 | 0.91–1.27 | 0.432 | 1.03 | 0.84–1.27 | 0.862 | 1.00 | 0.88–1.15 | 0.966 |
| Education level (Doctoral candidate) | 0.85 | 0.41–1.78 | 0.757 | 0.82 | 0.49–1.37 | 0.579 | 1.78 | 0.60–5.29 | 0.385 | 0.36 | 0.14–0.92 | **0.049** | 0.35 | 0.12–1.00 | 0.105 | 1.10 | 0.65–1.86 | 0.780 |
| Age | 1.00 | 0.98–1.02 | 0.817 | 0.99 | 0.98–1.00 | 0.319 | 0.98 | 0.94–1.02 | 0.385 | 0.99 | 0.97–1.01 | 0.232 | 1.00 | 0.98–1.02 | 0.977 | 0.99 | 0.98–1.01 | 0.336 |
| BMI | 0.99 | 0.99–1.00 | **0.022** | 1.00 | 0.99–1.00 | 0.053 | 0.99 | 0.98–1.00 | **0.017** | 1.00 | 0.99–1.00 | 0.116 | 1.00 | 0.99–1.01 | 0.862 | 1.00 | 1.00–1.00 | 0.700 |
| Gender (Female) | 1.73 | 1.61–1.85 | **< 0.001** | 1.66 | 1.59–1.72 | **< 0.001** | 2.03 | 1.78–2.31 | **< 0.001** | 1.19 | 1.12–1.26 | **< 0.001** | 1.21 | 1.12–1.30 | **< 0.001** | 1.26 | 1.20–1.31 | **< 0.001** |
| Gender identification (Transgender) | 2.39 | 2.01–2.83 | **< 0.001** | 2.01 | 1.77–2.28 | **< 0.001** | 1.88 | 1.41–2.52 | **< 0.001** | 2.40 | 2.06–2.80 | **< 0.001** | 2.74 | 2.29–3.28 | **< 0.001** | 1.84 | 1.61–2.12 | **< 0.001** |
| Gender identification (Nonbinary or genderquee) | 3.91 | 3.41–4.47 | **< 0.001** | 3.28 | 2.92–3.68 | **< 0.001** | 3.64 | 2.93–4.51 | **< 0.001** | 2.98 | 2.61–3.40 | **< 0.001** | 3.03 | 2.60–3.54 | **< 0.001** | 2.45 | 2.17–2.76 | **< 0.001** |
| Gender identification (Uncertainty) | 2.04 | 1.71–2.44 | **< 0.001** | 1.74 | 1.52–1.98 | **< 0.001** | 1.98 | 1.46–2.69 | **< 0.001** | 2.17 | 1.86–2.54 | **< 0.001** | 1.92 | 1.58–2.34 | **< 0.001** | 1.90 | 1.66–2.17 | **< 0.001** |
| Gender identification (Questioning) | 1.08 | 0.94–1.24 | 0.463 | 0.87 | 0.80–0.95 | **0.006** | 1.15 | 0.91–1.45 | 0.366 | 1.20 | 1.07–1.34 | **0.002** | 1.08 | 0.93–1.26 | 0.502 | 1.09 | 0.99–1.20 | 0.104 |
| Ethnicity (Han) | 1.03 | 0.94–1.13 | 0.701 | 1.02 | 0.96–1.08 | 0.623 | 0.96 | 0.82–1.12 | 0.629 | 0.95 | 0.88–1.03 | 0.304 | 0.97 | 0.88–1.07 | 0.767 | 0.98 | 0.92–1.05 | 0.722 |
| Family category (Extended family [three generations or more]) | 0.98 | 0.91–1.06 | 0.744 | 1.00 | 0.96–1.05 | 0.956 | 0.97 | 0.84–1.12 | 0.733 | 0.98 | 0.92–1.05 | 0.655 | 1.03 | 0.94–1.12 | 0.767 | 1.05 | 1.00–1.11 | 0.088 |
| Family category (Foster/Adoptive family) | 1.81 | 1.04–3.15 | 0.074 | 1.79 | 1.17–2.74 | **0.015** | 1.86 | 0.79–4.38 | 0.262 | 2.12 | 1.37–3.27 | **< 0.001** | 2.26 | 1.35–3.78 | **0.005** | 1.33 | 0.83–2.13 | 0.336 |
| Family category (Blended family) | 1.72 | 1.50–1.97 | **< 0.001** | 1.71 | 1.55–1.89 | **< 0.001** | 1.89 | 1.51–2.36 | **< 0.001** | 1.70 | 1.49–1.92 | **< 0.001** | 1.58 | 1.35–1.86 | **< 0.001** | 1.52 | 1.37–1.70 | **< 0.001** |
| Family category (Single-parent family) | 1.52 | 1.38–1.68 | **< 0.001** | 1.54 | 1.43–1.65 | **< 0.001** | 1.46 | 1.23–1.74 | **< 0.001** | 1.42 | 1.29–1.56 | **< 0.001** | 1.33 | 1.18–1.50 | **< 0.001** | 1.32 | 1.22–1.43 | **< 0.001** |
| Family category (Left-behind family) | 2.07 | 1.71–2.51 | **< 0.001** | 2.23 | 1.95–2.55 | **< 0.001** | 1.93 | 1.42–2.60 | **< 0.001** | 1.74 | 1.47–2.07 | **< 0.001** | 1.82 | 1.47–2.25 | **< 0.001** | 1.46 | 1.26–1.70 | **< 0.001** |
| Fathers education level (Primary school graduation) | 0.69 | 0.53–0.90 | **0.016** | 0.99 | 0.82–1.19 | 0.956 | 0.54 | 0.35–0.85 | **0.017** | 0.67 | 0.53–0.84 | **< 0.001** | 0.93 | 0.67–1.28 | 0.809 | 0.94 | 0.76–1.15 | 0.625 |
| Fathers education level (Junior high school graduation) | 0.62 | 0.47–0.81 | **< 0.001** | 0.85 | 0.71–1.03 | 0.143 | 0.44 | 0.28–0.69 | **< 0.001** | 0.57 | 0.45–0.72 | **< 0.001** | 0.79 | 0.57–1.10 | 0.305 | 0.87 | 0.71–1.07 | 0.288 |
| Fathers education level (High school/Technical school graduation) | 0.66 | 0.50–0.87 | **0.008** | 0.90 | 0.75–1.10 | 0.415 | 0.44 | 0.28–0.70 | **< 0.001** | 0.57 | 0.45–0.73 | **< 0.001** | 0.84 | 0.60–1.17 | 0.502 | 0.91 | 0.74–1.13 | 0.529 |
| Fathers education level (Associate degree graduation) | 0.74 | 0.56–0.99 | 0.085 | 1.04 | 0.86–1.27 | 0.772 | 0.52 | 0.32–0.84 | **0.017** | 0.61 | 0.48–0.79 | **< 0.001** | 0.87 | 0.61–1.23 | 0.639 | 0.92 | 0.74–1.14 | 0.542 |
| Fathers education level (Bachelor's degree graduation) | 0.70 | 0.52–0.94 | **0.037** | 1.08 | 0.89–1.33 | 0.570 | 0.55 | 0.33–0.89 | **0.033** | 0.68 | 0.53–0.89 | **0.006** | 0.92 | 0.65–1.32 | 0.809 | 0.92 | 0.73–1.15 | 0.544 |
| Fathers education level (Master's/Ph.D./ Postdoctoral graduation) | 0.77 | 0.51–1.17 | 0.377 | 0.99 | 0.75–1.31 | 0.956 | 0.41 | 0.20–0.86 | **0.037** | 0.92 | 0.64–1.32 | 0.655 | 1.52 | 0.96–2.40 | 0.147 | 0.99 | 0.72–1.35 | 0.961 |
| Fathers education level (Not sure) | 0.83 | 0.58–1.18 | 0.463 | 0.92 | 0.72–1.19 | 0.644 | 0.71 | 0.38–1.34 | 0.385 | 0.86 | 0.62–1.18 | 0.396 | 0.84 | 0.55–1.31 | 0.648 | 1.05 | 0.80–1.38 | 0.783 |
| Fathers education level (Others [father's role missing, such as the father passing away, etc.]) | 0.74 | 0.54–1.01 | 0.109 | 1.01 | 0.81–1.26 | 0.956 | 0.59 | 0.35–0.99 | 0.083 | 0.68 | 0.52–0.90 | **0.011** | 1.03 | 0.70–1.50 | 0.958 | 1.02 | 0.80–1.30 | 0.938 |
| Mothers education level (Primary school graduation) | 0.94 | 0.77–1.14 | 0.685 | 0.89 | 0.79–1.01 | 0.123 | 1.10 | 0.79–1.54 | 0.627 | 0.96 | 0.81–1.13 | 0.648 | 0.96 | 0.77–1.19 | 0.816 | 0.90 | 0.79–1.04 | 0.230 |
| Mothers education level (Junior high school graduation) | 0.85 | 0.69–1.03 | 0.172 | 0.80 | 0.70–0.90 | **< 0.001** | 0.82 | 0.59–1.16 | 0.381 | 0.86 | 0.72–1.01 | 0.104 | 0.85 | 0.68–1.05 | 0.263 | 0.81 | 0.71–0.93 | **0.005** |
| Mothers education level (High school/Technical school graduation) | 0.92 | 0.75–1.13 | 0.618 | 0.82 | 0.72–0.93 | **0.006** | 0.90 | 0.63–1.28 | 0.615 | 0.87 | 0.73–1.04 | 0.172 | 0.88 | 0.70–1.11 | 0.491 | 0.74 | 0.64–0.85 | **< 0.001** |
| Mothers education level (Associate degree graduation) | 1.06 | 0.85–1.32 | 0.744 | 0.88 | 0.76–1.01 | 0.123 | 0.87 | 0.59–1.28 | 0.557 | 0.90 | 0.74–1.09 | 0.329 | 1.01 | 0.79–1.30 | 0.959 | 0.80 | 0.69–0.94 | **0.013** |
| Mothers education level (Bachelor's degree graduation) | 1.07 | 0.85–1.36 | 0.710 | 0.77 | 0.67–0.90 | **0.002** | 0.93 | 0.61–1.40 | 0.733 | 0.84 | 0.68–1.03 | 0.116 | 0.93 | 0.72–1.21 | 0.770 | 0.77 | 0.66–0.91 | **0.005** |
| Mothers education level (Master's/Ph.D./ Postdoctoral graduation) | 1.24 | 0.83–1.84 | 0.463 | 0.99 | 0.76–1.29 | 0.956 | 1.55 | 0.81–2.98 | 0.301 | 0.80 | 0.55–1.15 | 0.276 | 1.02 | 0.66–1.57 | 0.959 | 0.88 | 0.65–1.18 | 0.529 |
| Mothers education level (Not sure) | 0.98 | 0.72–1.33 | 0.884 | 0.83 | 0.68–1.02 | 0.123 | 0.67 | 0.36–1.22 | 0.301 | 0.89 | 0.67–1.17 | 0.432 | 0.83 | 0.57–1.19 | 0.502 | 0.60 | 0.48–0.75 | **< 0.001** |
| Mothers education level (Others [mother's role missing, such as the mother passing away, etc.]) | 1.02 | 0.76–1.38 | 0.884 | 1.13 | 0.93–1.39 | 0.309 | 1.19 | 0.74–1.91 | 0.557 | 1.10 | 0.86–1.41 | 0.496 | 0.97 | 0.69–1.34 | 0.916 | 0.92 | 0.74–1.14 | 0.542 |
| Household income￥6000–14 000 | 1.01 | 0.94–1.08 | 0.865 | 1.05 | 1.01–1.10 | **0.049** | 0.84 | 0.74–0.96 | **0.019** | 0.88 | 0.82–0.93 | **< 0.001** | 0.90 | 0.83–0.98 | **0.025** | 0.94 | 0.89–0.98 | **0.019** |
| Household income￥14 000–23 000 | 1.01 | 0.93–1.11 | 0.817 | 1.10 | 1.04–1.16 | **< 0.001** | 0.93 | 0.80–1.08 | 0.416 | 0.83 | 0.77–0.90 | **< 0.001** | 0.83 | 0.76–0.92 | **< 0.001** | 0.87 | 0.81–0.92 | **< 0.001** |
| Household income￥23 000–36 000 | 0.98 | 0.88–1.09 | 0.757 | 1.08 | 1.01–1.15 | **0.039** | 0.82 | 0.67–0.99 | 0.079 | 0.81 | 0.73–0.89 | **< 0.001** | 0.94 | 0.84–1.06 | 0.502 | 0.87 | 0.81–0.94 | **< 0.001** |
| Household income￥36 000–70 000 | 1.04 | 0.93–1.18 | 0.668 | 1.09 | 1.01–1.18 | **0.045** | 0.87 | 0.69–1.09 | 0.339 | 0.75 | 0.67–0.83 | **< 0.001** | 0.81 | 0.70–0.93 | **0.007** | 0.90 | 0.83–0.98 | **0.033** |
| Household income (＞￥70,000) | 0.96 | 0.83–1.11 | 0.723 | 0.97 | 0.89–1.07 | 0.674 | 1.04 | 0.81–1.34 | 0.757 | 0.79 | 0.69–0.90 | **< 0.001** | 1.03 | 0.89–1.21 | 0.809 | 0.90 | 0.82–1.00 | 0.092 |
| Drinking (≤1 time/month) | 1.60 | 1.49–1.71 | **< 0.001** | 1.45 | 1.39–1.51 | **< 0.001** | 1.29 | 1.14–1.45 | **< 0.001** | 1.33 | 1.25–1.40 | **< 0.001** | 1.14 | 1.06–1.23 | **< 0.001** | 1.38 | 1.32–1.45 | **< 0.001** |
| Drinking 2–4 times/month | 2.59 | 2.36–2.85 | **< 0.001** | 1.79 | 1.68–1.90 | **< 0.001** | 2.08 | 1.76–2.46 | **< 0.001** | 1.96 | 1.80–2.13 | **< 0.001** | 1.66 | 1.49–1.85 | **< 0.001** | 1.90 | 1.78–2.03 | **< 0.001** |
| Drinking 2–3 times/week | 3.30 | 2.79–3.90 | **< 0.001** | 2.17 | 1.92–2.47 | **< 0.001** | 3.18 | 2.41–4.18 | **< 0.001** | 2.99 | 2.58–3.46 | **< 0.001** | 2.74 | 2.29–3.29 | **< 0.001** | 2.65 | 2.33–3.01 | **< 0.001** |
| Drinking (≥4 times/week) | 3.30 | 2.69–4.06 | **< 0.001** | 2.03 | 1.71–2.41 | **< 0.001** | 4.03 | 2.97–5.45 | **< 0.001** | 2.98 | 2.47–3.60 | **< 0.001** | 2.81 | 2.24–3.52 | **< 0.001** | 2.94 | 2.49–3.47 | **< 0.001** |
| Smoking (Yes) | 1.82 | 1.67–1.99 | **< 0.001** | 1.14 | 1.08–1.21 | **< 0.001** | 1.94 | 1.66–2.27 | **< 0.001** | 1.70 | 1.58–1.84 | **< 0.001** | 1.46 | 1.32–1.61 | **< 0.001** | 1.53 | 1.44–1.62 | **< 0.001** |
| Observations | 79 011 | | | 79 011 | | | 79 011 | | | 79 011 | | | 79 011 | | | 79 011 | | |
| R^2^/R^2^ adjusted | 0.052/0.052 | | | 0.035/0.035 | | | 0.050/0.049 | | | 0.042/0.041 | | | 0.032/0.032 | | | 0.027/0.026 | | |

Abbreviations: CI, confidence interval; FDR, false discovery rate; NSSI, non-suicidal self-injury; PTSD, post-traumatic stress disorder; SA, suicide attempt; SI, suicidal ideation.

| S Table 8. Exercise frequency as determined by logistic generalised additive modelling (GAM) (N=79 011). | | | | | | | | | | | | | | | | | | |
| --- | --- | --- | --- | --- | --- | --- | --- | --- | --- | --- | --- | --- | --- | --- | --- | --- | --- | --- |
|  | **Lifetime NSSI** | | | **Lifetime SI** | | | **Lifetime SA** | | | **Depression** | | | **Anxiety** | | | **PTSD** | | |
| *Predictors* | *Odds ratios* | *CI (95%)* | *P value (fdr)* | *Odds ratios* | *CI (95%)* | *P value (fdr)* | *Odds ratios* | *CI (95%)* | *P value (fdr)* | *Odds ratios* | *CI (95%)* | *P value (fdr)* | *Odds ratios* | *CI (95%)* | *P value (fdr)* | *Odds ratios* | *CI (95%)* | *P value (fdr)* |
| Education (2) | 1.00 | 0.93–1.07 | 0.998 | 1.00 | 0.96–1.04 | 0.975 | 0.94 | 0.83–1.06 | 0.436 | 0.93 | 0.88–0.99 | **0.042** | 0.99 | 0.92–1.07 | 0.880 | 0.95 | 0.90–0.99 | 0.052 |
| Education (3) | 0.97 | 0.89–1.07 | 0.755 | 1.02 | 0.96–1.08 | 0.685 | 0.91 | 0.77–1.07 | 0.360 | 0.98 | 0.91–1.07 | 0.787 | 0.99 | 0.89–1.10 | 0.880 | 0.97 | 0.91–1.03 | 0.447 |
| Education (4) | 0.97 | 0.84–1.13 | 0.869 | 0.95 | 0.87–1.04 | 0.402 | 0.88 | 0.68–1.15 | 0.460 | 0.98 | 0.86–1.11 | 0.787 | 1.08 | 0.92–1.26 | 0.604 | 1.00 | 0.91–1.11 | 0.970 |
| Education (5) | 1.11 | 0.92–1.34 | 0.471 | 0.98 | 0.86–1.10 | 0.844 | 0.87 | 0.62–1.24 | 0.550 | 0.87 | 0.73–1.04 | 0.190 | 0.99 | 0.79–1.23 | 0.972 | 0.98 | 0.85–1.12 | 0.817 |
| Education (6) | 1.16 | 0.93–1.45 | 0.350 | 1.02 | 0.89–1.18 | 0.869 | 1.05 | 0.71–1.55 | 0.903 | 1.00 | 0.82–1.22 | 1.000 | 1.17 | 0.91–1.50 | 0.415 | 1.00 | 0.85–1.17 | 0.983 |
| Education (7) | 1.10 | 0.74–1.62 | 0.812 | 0.99 | 0.77–1.28 | 0.977 | 1.00 | 0.51–1.98 | 1.000 | 1.20 | 0.86–1.66 | 0.390 | 1.02 | 0.65–1.60 | 0.972 | 0.99 | 0.75–1.31 | 0.983 |
| Education (8) | 0.85 | 0.33–2.21 | 0.888 | 0.75 | 0.42–1.36 | 0.494 | 0.00 | 0.00–Inf | 1.000 | 0.87 | 0.38–1.95 | 0.787 | 0.45 | 0.11–1.90 | 0.488 | 0.43 | 0.19–0.96 | 0.068 |
| Education (9) | 1.81 | 0.81–4.03 | 0.272 | 0.60 | 0.30–1.19 | 0.245 | 0.58 | 0.08–4.39 | 0.680 | 0.57 | 0.20–1.62 | 0.390 | 0.53 | 0.13–2.26 | 0.628 | 0.82 | 0.40–1.67 | 0.689 |
| Education (10) | 0.00 | 0.00–Inf | 0.998 | 0.96 | 0.42–2.19 | 0.975 | 2.64 | 0.58–11.91 | 0.305 | 0.00 | 0.00–Inf | 1.000 | 0.52 | 0.07–3.88 | 0.710 | 1.35 | 0.59–3.08 | 0.583 |
| Education (11) | 4.54 | 0.86–23.87 | 0.149 | 1.58 | 0.37–6.84 | 0.703 | 5.18 | 0.56–47.72 | 0.241 | 0.00 | 0.00–Inf | 1.000 | 0.00 | 0.00–Inf | 0.999 | 2.67 | 0.61–11.60 | 0.297 |
| Education (12) | 0.00 | 0.00–Inf | 0.998 | 0.92 | 0.28–3.00 | 0.975 | 0.00 | 0.00–Inf | 1.000 | 0.54 | 0.07–4.28 | 0.667 | 1.04 | 0.13–8.29 | 0.988 | 0.28 | 0.04–2.16 | 0.318 |
| Age | 0.99 | 0.96–1.01 | 0.629 | 0.99 | 0.98–1.01 | 0.473 | 1.01 | 0.96–1.06 | 0.814 | 0.99 | 0.97–1.02 | 0.649 | 0.99 | 0.96–1.02 | 0.764 | 0.99 | 0.98–1.01 | 0.688 |
| BMI | 0.99 | 0.99–1.00 | **0.038** | 1.00 | 0.99–1.00 | 0.059 | 0.99 | 0.98–0.99 | **0.006** | 1.00 | 0.99–1.00 | 0.258 | 1.00 | 1.00–1.01 | 0.777 | 1.00 | 1.00–1.00 | 0.575 |
| Gender (Female) | 1.76 | 1.65–1.87 | **< 0.001** | 1.73 | 1.67–1.80 | **< 0.001** | 2.15 | 1.92–2.41 | **< 0.001** | 1.17 | 1.11–1.23 | **< 0.001** | 1.20 | 1.12–1.28 | **< 0.001** | 1.23 | 1.18–1.28 | **< 0.001** |
| Gender identification (Transgender) | 2.33 | 1.99–2.74 | **< 0.001** | 1.99 | 1.77–2.25 | **< 0.001** | 2.03 | 1.54–2.66 | **< 0.001** | 2.28 | 1.97–2.63 | **< 0.001** | 2.54 | 2.15–3.01 | **< 0.001** | 1.81 | 1.59–2.06 | **< 0.001** |
| Gender identification (Nonbinary or genderquee) | 3.99 | 3.52–4.52 | **< 0.001** | 3.41 | 3.06–3.80 | **< 0.001** | 3.62 | 2.97–4.41 | **< 0.001** | 3.10 | 2.75–3.51 | **< 0.001** | 3.13 | 2.72–3.61 | **< 0.001** | 2.47 | 2.21–2.76 | **< 0.001** |
| Gender identification (Uncertainty) | 2.14 | 1.81–2.52 | **< 0.001** | 1.69 | 1.49–1.90 | **< 0.001** | 1.94 | 1.46–2.57 | **< 0.001** | 2.14 | 1.85–2.48 | **< 0.001** | 1.92 | 1.59–2.31 | **< 0.001** | 1.87 | 1.64–2.13 | **< 0.001** |
| Gender identification (Questioning) | 1.05 | 0.92–1.18 | 0.683 | 0.85 | 0.78–0.92 | **< 0.001** | 1.12 | 0.90–1.38 | 0.436 | 1.14 | 1.03–1.26 | **0.024** | 1.03 | 0.89–1.18 | 0.830 | 1.06 | 0.97–1.15 | 0.297 |
| Ethnicity (Han) | 1.03 | 0.95–1.13 | 0.650 | 1.03 | 0.97–1.08 | 0.502 | 0.96 | 0.83–1.11 | 0.672 | 0.97 | 0.90–1.04 | 0.440 | 0.98 | 0.89–1.08 | 0.793 | 1.00 | 0.94–1.06 | 0.970 |
| Family category (Extended family [three generations or more]) | 1.00 | 0.93–1.07 | 0.998 | 1.00 | 0.96–1.05 | 0.977 | 0.95 | 0.83–1.08 | 0.550 | 0.99 | 0.93–1.05 | 0.850 | 1.02 | 0.95–1.11 | 0.718 | 1.07 | 1.02–1.12 | **0.017** |
| Family category (Foster/Adoptive family) | 1.86 | 1.10–3.12 | **0.048** | 1.96 | 1.34–2.88 | **< 0.001** | 1.93 | 0.87–4.26 | 0.195 | 2.39 | 1.55–3.68 | **< 0.001** | 2.70 | 1.64–4.46 | **< 0.001** | 1.38 | 0.91–2.11 | 0.215 |
| Family category (Blended family) | 1.80 | 1.58–2.05 | **< 0.001** | 1.71 | 1.56–1.87 | **< 0.001** | 2.01 | 1.63–2.48 | **< 0.001** | 1.73 | 1.54–1.94 | **< 0.001** | 1.63 | 1.41–1.90 | **< 0.001** | 1.52 | 1.37–1.68 | **< 0.001** |
| Family category (Single-parent family) | 1.58 | 1.44–1.74 | **< 0.001** | 1.54 | 1.45–1.64 | **< 0.001** | 1.59 | 1.36–1.87 | **< 0.001** | 1.43 | 1.32–1.56 | **< 0.001** | 1.36 | 1.22–1.52 | **< 0.001** | 1.28 | 1.19–1.37 | **< 0.001** |
| Family category (Left-behind family) | 2.15 | 1.81–2.56 | **< 0.001** | 2.27 | 2.01–2.57 | **< 0.001** | 2.20 | 1.67–2.91 | **< 0.001** | 1.79 | 1.53–2.09 | **< 0.001** | 1.80 | 1.47–2.19 | **< 0.001** | 1.58 | 1.38–1.81 | **< 0.001** |
| Fathers education level (Primary school graduation) | 0.67 | 0.52–0.85 | **0.003** | 0.95 | 0.80–1.13 | 0.720 | 0.66 | 0.45–0.96 | 0.082 | 0.68 | 0.55–0.83 | **< 0.001** | 0.90 | 0.68–1.19 | 0.648 | 0.91 | 0.76–1.09 | 0.418 |
| Fathers education level (Junior high school graduation) | 0.59 | 0.46–0.75 | **< 0.001** | 0.85 | 0.71–1.00 | 0.091 | 0.55 | 0.37–0.80 | **0.007** | 0.59 | 0.48–0.72 | **< 0.001** | 0.79 | 0.60–1.05 | 0.223 | 0.86 | 0.71–1.03 | 0.156 |
| Fathers education level (High school/Technical school graduation) | 0.65 | 0.51–0.83 | **0.002** | 0.91 | 0.77–1.08 | 0.418 | 0.55 | 0.37–0.81 | **0.009** | 0.61 | 0.50–0.75 | **< 0.001** | 0.85 | 0.64–1.13 | 0.469 | 0.89 | 0.74–1.07 | 0.318 |
| Fathers education level (Associate degree graduation) | 0.72 | 0.56–0.93 | **0.036** | 1.05 | 0.88–1.26 | 0.720 | 0.64 | 0.42–0.98 | 0.093 | 0.65 | 0.52–0.81 | **< 0.001** | 0.89 | 0.66–1.21 | 0.655 | 0.88 | 0.73–1.07 | 0.318 |
| Fathers education level (Bachelor's degree graduation) | 0.69 | 0.53–0.90 | **0.019** | 1.11 | 0.92–1.33 | 0.409 | 0.72 | 0.47–1.11 | 0.237 | 0.74 | 0.59–0.93 | **0.017** | 0.96 | 0.71–1.31 | 0.880 | 0.91 | 0.75–1.11 | 0.497 |
| Fathers education level (Master's/Ph.D./ Postdoctoral graduation) | 0.69 | 0.48–1.00 | 0.103 | 0.97 | 0.75–1.25 | 0.910 | 0.51 | 0.27–0.98 | 0.097 | 0.92 | 0.67–1.26 | 0.691 | 1.36 | 0.91–2.02 | 0.279 | 0.89 | 0.67–1.17 | 0.514 |
| Fathers education level (Not sure) | 0.72 | 0.52–1.00 | 0.101 | 0.87 | 0.69–1.09 | 0.371 | 0.82 | 0.48–1.38 | 0.550 | 0.81 | 0.62–1.07 | 0.202 | 0.82 | 0.56–1.20 | 0.510 | 0.98 | 0.77–1.25 | 0.958 |
| Fathers education level (Others [father's role missing, such as the father passing away, etc.]) | 0.68 | 0.51–0.91 | **0.025** | 0.96 | 0.79–1.18 | 0.844 | 0.72 | 0.46–1.14 | 0.258 | 0.65 | 0.51–0.83 | **< 0.001** | 0.95 | 0.69–1.33 | 0.880 | 0.99 | 0.80–1.23 | 0.970 |
| Mothers education level (Primary school graduation) | 0.90 | 0.75–1.08 | 0.461 | 0.85 | 0.76–0.95 | **0.010** | 0.91 | 0.68–1.22 | 0.609 | 0.89 | 0.77–1.03 | 0.186 | 0.88 | 0.72–1.07 | 0.392 | 0.87 | 0.77–0.98 | **0.049** |
| Mothers education level (Junior high school graduation) | 0.81 | 0.68–0.98 | 0.062 | 0.75 | 0.67–0.84 | **< 0.001** | 0.68 | 0.51–0.91 | **0.030** | 0.80 | 0.69–0.93 | **0.006** | 0.79 | 0.65–0.95 | **0.040** | 0.79 | 0.70–0.90 | **< 0.001** |
| Mothers education level (High school/Technical school graduation) | 0.90 | 0.74–1.08 | 0.452 | 0.76 | 0.68–0.86 | **< 0.001** | 0.75 | 0.55–1.02 | 0.151 | 0.80 | 0.69–0.94 | **0.009** | 0.82 | 0.67–1.01 | 0.133 | 0.73 | 0.64–0.83 | **< 0.001** |
| Mothers education level (Associate degree graduation) | 1.04 | 0.85–1.27 | 0.869 | 0.84 | 0.74–0.96 | **0.018** | 0.74 | 0.53–1.05 | 0.181 | 0.85 | 0.72–1.01 | 0.103 | 0.95 | 0.76–1.18 | 0.784 | 0.81 | 0.70–0.93 | **0.006** |
| Mothers education level (Bachelor's degree graduation) | 1.06 | 0.86–1.32 | 0.756 | 0.76 | 0.66–0.87 | **< 0.001** | 0.76 | 0.53–1.09 | 0.237 | 0.80 | 0.67–0.96 | **0.029** | 0.91 | 0.72–1.15 | 0.648 | 0.79 | 0.68–0.91 | **0.004** |
| Mothers education level (Master's/Ph.D./ Postdoctoral graduation) | 1.38 | 0.97–1.98 | 0.149 | 1.00 | 0.79–1.28 | 0.985 | 1.54 | 0.87–2.75 | 0.237 | 0.87 | 0.63–1.22 | 0.532 | 1.16 | 0.79–1.71 | 0.648 | 0.95 | 0.72–1.25 | 0.817 |
| Mothers education level (Not sure) | 1.03 | 0.77–1.36 | 0.955 | 0.83 | 0.69–1.00 | 0.091 | 0.56 | 0.34–0.93 | 0.069 | 0.90 | 0.71–1.14 | 0.486 | 0.78 | 0.56–1.08 | 0.279 | 0.64 | 0.51–0.78 | **< 0.001** |
| Mothers education level (Others [mother's role missing, such as the mother passing away, etc.]) | 1.01 | 0.77–1.32 | 0.998 | 1.03 | 0.86–1.23 | 0.901 | 0.96 | 0.62–1.48 | 0.908 | 1.04 | 0.83–1.31 | 0.787 | 0.88 | 0.65–1.20 | 0.648 | 0.92 | 0.75–1.12 | 0.514 |
| Household income￥6000–14 000 | 1.02 | 0.95–1.09 | 0.783 | 1.07 | 1.02–1.11 | **0.005** | 0.85 | 0.75–0.96 | **0.022** | 0.89 | 0.84–0.95 | **< 0.001** | 0.91 | 0.85–0.99 | **0.047** | 0.94 | 0.90–0.99 | **0.030** |
| Household income￥14 000–23 000 | 0.99 | 0.92–1.08 | 0.955 | 1.11 | 1.06–1.17 | **< 0.001** | 0.93 | 0.81–1.08 | 0.460 | 0.83 | 0.77–0.89 | **< 0.001** | 0.86 | 0.78–0.94 | **0.003** | 0.87 | 0.82–0.92 | **< 0.001** |
| Household income￥23 000–36 000 | 0.98 | 0.89–1.09 | 0.889 | 1.09 | 1.03–1.16 | **0.009** | 0.85 | 0.71–1.02 | 0.163 | 0.81 | 0.75–0.89 | **< 0.001** | 0.94 | 0.84–1.05 | 0.469 | 0.88 | 0.83–0.95 | **< 0.001** |
| Household income￥36 000–70 000 | 1.04 | 0.93–1.17 | 0.650 | 1.11 | 1.04–1.19 | **0.006** | 0.87 | 0.71–1.07 | 0.303 | 0.78 | 0.71–0.87 | **< 0.001** | 0.85 | 0.75–0.97 | **0.041** | 0.91 | 0.84–0.98 | **0.030** |
| Household income (＞￥70,000) | 0.95 | 0.84–1.09 | 0.683 | 0.97 | 0.89–1.05 | 0.576 | 0.98 | 0.78–1.23 | 0.908 | 0.77 | 0.69–0.87 | **< 0.001** | 1.03 | 0.90–1.19 | 0.788 | 0.90 | 0.82–0.99 | 0.051 |
| Drinking (≤1 time/month) | 1.57 | 1.48–1.68 | **< 0.001** | 1.44 | 1.39–1.49 | **< 0.001** | 1.31 | 1.18–1.47 | **< 0.001** | 1.33 | 1.26–1.40 | **< 0.001** | 1.14 | 1.07–1.22 | **< 0.001** | 1.37 | 1.31–1.42 | **< 0.001** |
| Drinking 2–4 times/month | 2.54 | 2.33–2.77 | **< 0.001** | 1.79 | 1.69–1.89 | **< 0.001** | 2.17 | 1.87–2.52 | **< 0.001** | 1.92 | 1.78–2.07 | **< 0.001** | 1.61 | 1.46–1.77 | **< 0.001** | 1.89 | 1.78–2.01 | **< 0.001** |
| Drinking 2–3 times/week | 3.15 | 2.71–3.66 | **< 0.001** | 2.09 | 1.86–2.34 | **< 0.001** | 3.30 | 2.59–4.20 | **< 0.001** | 2.93 | 2.56–3.34 | **< 0.001** | 2.66 | 2.26–3.12 | **< 0.001** | 2.58 | 2.30–2.90 | **< 0.001** |
| Drinking (≥4 times/week) | 3.65 | 3.01–4.41 | **< 0.001** | 2.02 | 1.73–2.37 | **< 0.001** | 4.47 | 3.37–5.93 | **< 0.001** | 3.17 | 2.67–3.76 | **< 0.001** | 3.03 | 2.47–3.72 | **< 0.001** | 3.10 | 2.66–3.60 | **< 0.001** |
| Smoking (Yes) | 1.78 | 1.65–1.92 | **< 0.001** | 1.13 | 1.07–1.19 | **< 0.001** | 1.84 | 1.60–2.10 | **< 0.001** | 1.62 | 1.52–1.73 | **< 0.001** | 1.40 | 1.29–1.53 | **< 0.001** | 1.49 | 1.41–1.57 | **< 0.001** |
| Smooth term (Exercise Frequency2) × Exercise Type double swing |  |  | **< 0.001** |  |  | **< 0.001** |  |  | 0.088 |  |  | **< 0.001** |  |  | **< 0.001** |  |  | **< 0.001** |
| Smooth term (Exercise Frequency2) × Exercise Typelow intensity aerobics |  |  | 0.570 |  |  | **< 0.001** |  |  | 0.181 |  |  | **< 0.001** |  |  | **< 0.001** |  |  | **< 0.001** |
| Smooth term (Exercise Frequency2) × Exercise Typemedium intensity aerobics |  |  | 0.955 |  |  | **< 0.001** |  |  | 0.181 |  |  | **< 0.001** |  |  | **< 0.001** |  |  | **< 0.001** |
| Smooth term (Exercise Frequency2) × Exercise Type single anaerobics |  |  | 0.101 |  |  | **< 0.001** |  |  | 0.260 |  |  | **< 0.001** |  |  | **< 0.001** |  |  | **< 0.001** |
| Smooth term (Exercise Frequency2) × Exercise Typeteam ball |  |  | **< 0.001** |  |  | **< 0.001** |  |  | **0.001** |  |  | **< 0.001** |  |  | **< 0.001** |  |  | **< 0.001** |
| Observations | 79 011 | | | 79 011 | | | 79 011 | | | 79 011 | | | 79 011 | | | 79 011 | | |
| R^2^ | 0.040 | | | 0.047 | | | 0.018 | | | 0.038 | | | 0.021 | | | 0.030 | | |
| AIC | 42583.296 | | | 89169.466 | | | 16850.072 | | | 52887.335 | | | 35823.020 | | | 75099.454 | | |

Abbreviations: CI, confidence interval; FDR, false discovery rate; NSSI, non-suicidal self-injury; PTSD, post-traumatic stress disorder; SA, suicide attempt; SI, suicidal ideation.

| S Table 9. Exercise duration as determined by logistic generalised additive modeling (GAM) (N=79 011). | | | | | | | | | | | | | | | | | | |
| --- | --- | --- | --- | --- | --- | --- | --- | --- | --- | --- | --- | --- | --- | --- | --- | --- | --- | --- |
|  | **Lifetime NSSI** | | | **Lifetime SI** | | | **Lifetime SA** | | | **Depression** | | | **Anxiety** | | | **PTSD** | | |
| *Predictors* | *Odds ratios* | *CI (95%)* | *P value (fdr)* | *Odds ratios* | *CI (95%)* | *P value (fdr)* | *Odds ratios* | *CI (95%)* | *P value (fdr)* | *Odds ratios* | *CI (95%)* | *P value (fdr)* | *Odds ratios* | *CI (95%)* | *P value (fdr)* | *Odds ratios* | *CI (95%)* | *P value (fdr)* |
| Education (2) | 1.00 | 0.93–1.07 | 0.989 | 1.00 | 0.95–1.04 | 0.883 | 0.94 | 0.83–1.06 | 0.430 | 0.93 | 0.88–0.99 | **0.032** | 0.99 | 0.92–1.07 | 0.851 | 0.95 | 0.90–0.99 | 0.051 |
| Education (3) | 0.97 | 0.89–1.07 | 0.737 | 1.02 | 0.96–1.08 | 0.744 | 0.91 | 0.77–1.07 | 0.361 | 0.98 | 0.91–1.06 | 0.727 | 0.99 | 0.89–1.10 | 0.860 | 0.97 | 0.91–1.03 | 0.417 |
| Education (4) | 0.97 | 0.84–1.12 | 0.808 | 0.94 | 0.86–1.03 | 0.323 | 0.88 | 0.68–1.14 | 0.442 | 0.97 | 0.85–1.10 | 0.698 | 1.07 | 0.91–1.25 | 0.647 | 1.00 | 0.91–1.11 | 0.992 |
| Education (5) | 1.10 | 0.91–1.33 | 0.499 | 0.97 | 0.86–1.09 | 0.753 | 0.87 | 0.61–1.23 | 0.540 | 0.85 | 0.72–1.02 | 0.119 | 0.97 | 0.78–1.21 | 0.860 | 0.97 | 0.84–1.11 | 0.733 |
| Education (6) | 1.17 | 0.93–1.46 | 0.305 | 1.03 | 0.89–1.19 | 0.828 | 1.05 | 0.71–1.55 | 0.894 | 1.00 | 0.82–1.22 | 1.000 | 1.17 | 0.91–1.50 | 0.411 | 1.00 | 0.85–1.17 | 0.992 |
| Education (7) | 1.10 | 0.75–1.63 | 0.791 | 0.99 | 0.77–1.28 | 0.967 | 0.99 | 0.50–1.96 | 1.000 | 1.18 | 0.85–1.65 | 0.420 | 1.00 | 0.64–1.58 | 1.000 | 0.99 | 0.75–1.30 | 0.987 |
| Education (8) | 0.85 | 0.33–2.19 | 0.855 | 0.72 | 0.40–1.29 | 0.408 | 0.00 | 0.00–Inf | 1.000 | 0.81 | 0.36–1.82 | 0.698 | 0.43 | 0.10–1.80 | 0.450 | 0.42 | 0.19–0.93 | 0.057 |
| Education (9) | 1.78 | 0.80–3.96 | 0.277 | 0.58 | 0.29–1.16 | 0.212 | 0.57 | 0.07–4.31 | 0.667 | 0.56 | 0.19–1.59 | 0.362 | 0.52 | 0.12–2.20 | 0.633 | 0.81 | 0.40–1.66 | 0.681 |
| Education (10) | 0.00 | 0.00–Inf | 0.999 | 1.03 | 0.45–2.35 | 0.967 | 2.64 | 0.58–11.95 | 0.314 | 0.00 | 0.00–Inf | 1.000 | 0.52 | 0.07–3.94 | 0.674 | 1.42 | 0.62–3.25 | 0.520 |
| Education (11) | 4.12 | 0.77–21.89 | 0.175 | 1.44 | 0.33–6.26 | 0.780 | 4.78 | 0.51–44.56 | 0.263 | 0.00 | 0.00–Inf | 1.000 | 0.00 | 0.00–Inf | 1.000 | 2.45 | 0.56–10.65 | 0.334 |
| Education (12) | 0.00 | 0.00–Inf | 0.999 | 0.90 | 0.27–2.95 | 0.890 | 0.00 | 0.00–Inf | 1.000 | 0.50 | 0.06–4.01 | 0.630 | 0.97 | 0.12–7.80 | 1.000 | 0.27 | 0.03–2.12 | 0.322 |
| Age | 0.99 | 0.96–1.02 | 0.662 | 0.99 | 0.98–1.01 | 0.612 | 1.01 | 0.96–1.06 | 0.776 | 1.00 | 0.97–1.02 | 0.763 | 0.99 | 0.97–1.02 | 0.850 | 1.00 | 0.98–1.01 | 0.793 |
| BMI | 0.99 | 0.99–1.00 | **0.032** | 1.00 | 0.99–1.00 | 0.070 | 0.99 | 0.98–0.99 | **0.006** | 1.00 | 0.99–1.00 | 0.308 | 1.00 | 1.00–1.01 | 0.710 | 1.00 | 1.00–1.00 | 0.624 |
| Gender (Female) | 1.69 | 1.59–1.80 | **< 0.001** | 1.68 | 1.62–1.75 | **< 0.001** | 2.09 | 1.87–2.35 | **< 0.001** | 1.14 | 1.08–1.20 | **< 0.001** | 1.18 | 1.10–1.26 | **< 0.001** | 1.21 | 1.16–1.26 | **< 0.001** |
| Gender identification (Transgender) | 2.36 | 2.01–2.76 | **< 0.001** | 2.02 | 1.79–2.28 | **< 0.001** | 2.03 | 1.54–2.66 | **< 0.001** | 2.30 | 1.99–2.66 | **< 0.001** | 2.57 | 2.17–3.05 | **< 0.001** | 1.82 | 1.60–2.07 | **< 0.001** |
| Gender identification (Nonbinary or genderquee) | 3.97 | 3.50–4.49 | **< 0.001** | 3.37 | 3.03–3.76 | **< 0.001** | 3.61 | 2.97–4.40 | **< 0.001** | 3.04 | 2.69–3.43 | **< 0.001** | 3.08 | 2.67–3.55 | **< 0.001** | 2.44 | 2.19–2.73 | **< 0.001** |
| Gender identification (Uncertainty) | 2.12 | 1.80–2.50 | **< 0.001** | 1.68 | 1.49–1.90 | **< 0.001** | 1.93 | 1.45–2.56 | **< 0.001** | 2.14 | 1.85–2.48 | **< 0.001** | 1.91 | 1.59–2.31 | **< 0.001** | 1.87 | 1.65–2.13 | **< 0.001** |
| Gender identification (Questioning) | 1.05 | 0.93–1.18 | 0.663 | 0.86 | 0.79–0.93 | **< 0.001** | 1.11 | 0.90–1.38 | 0.442 | 1.14 | 1.03–1.26 | **0.021** | 1.02 | 0.89–1.17 | 0.851 | 1.06 | 0.97–1.15 | 0.293 |
| Ethnicity (Han) | 1.02 | 0.94–1.11 | 0.774 | 1.02 | 0.96–1.07 | 0.753 | 0.95 | 0.82–1.10 | 0.573 | 0.95 | 0.88–1.02 | 0.234 | 0.96 | 0.88–1.06 | 0.647 | 0.99 | 0.93–1.05 | 0.820 |
| Family category (Extended family [three generations or more]) | 1.00 | 0.93–1.08 | 0.989 | 1.00 | 0.96–1.05 | 0.883 | 0.95 | 0.84–1.09 | 0.573 | 1.00 | 0.94–1.06 | 0.952 | 1.03 | 0.95–1.11 | 0.654 | 1.07 | 1.02–1.12 | **0.012** |
| Family category (Foster/Adoptive family) | 1.84 | 1.09–3.09 | **0.047** | 1.92 | 1.31–2.81 | **0.002** | 1.97 | 0.89–4.35 | 0.175 | 2.33 | 1.51–3.59 | **< 0.001** | 2.64 | 1.60–4.35 | **< 0.001** | 1.34 | 0.88–2.05 | 0.277 |
| Family category (Blended family) | 1.81 | 1.59–2.06 | **< 0.001** | 1.72 | 1.57–1.89 | **< 0.001** | 2.02 | 1.63–2.49 | **< 0.001** | 1.75 | 1.55–1.96 | **< 0.001** | 1.65 | 1.42–1.91 | **< 0.001** | 1.53 | 1.38–1.69 | **< 0.001** |
| Family category (Single-parent family) | 1.58 | 1.44–1.74 | **< 0.001** | 1.54 | 1.45–1.65 | **< 0.001** | 1.59 | 1.36–1.87 | **< 0.001** | 1.43 | 1.32–1.56 | **< 0.001** | 1.36 | 1.22–1.51 | **< 0.001** | 1.28 | 1.19–1.37 | **< 0.001** |
| Family category (Left-behind family) | 2.15 | 1.81–2.55 | **< 0.001** | 2.26 | 1.99–2.55 | **< 0.001** | 2.21 | 1.67–2.92 | **< 0.001** | 1.78 | 1.52–2.08 | **< 0.001** | 1.79 | 1.47–2.18 | **< 0.001** | 1.58 | 1.38–1.81 | **< 0.001** |
| Fathers education level (Primary school graduation) | 0.67 | 0.52–0.85 | **0.003** | 0.95 | 0.80–1.13 | 0.753 | 0.66 | 0.45–0.96 | 0.077 | 0.67 | 0.55–0.83 | **< 0.001** | 0.89 | 0.67–1.18 | 0.647 | 0.91 | 0.76–1.09 | 0.417 |
| Fathers education level (Junior high school graduation) | 0.59 | 0.46–0.75 | **< 0.001** | 0.84 | 0.71–1.00 | 0.084 | 0.55 | 0.37–0.80 | **0.007** | 0.59 | 0.48–0.72 | **< 0.001** | 0.78 | 0.59–1.04 | 0.199 | 0.86 | 0.71–1.02 | 0.153 |
| Fathers education level (High school/Technical school graduation) | 0.65 | 0.51–0.84 | **0.002** | 0.91 | 0.76–1.08 | 0.408 | 0.55 | 0.37–0.82 | **0.010** | 0.61 | 0.50–0.75 | **< 0.001** | 0.84 | 0.63–1.12 | 0.448 | 0.89 | 0.74–1.07 | 0.329 |
| Fathers education level (Associate degree graduation) | 0.72 | 0.56–0.94 | **0.032** | 1.05 | 0.87–1.25 | 0.780 | 0.65 | 0.43–0.98 | 0.083 | 0.65 | 0.52–0.80 | **< 0.001** | 0.89 | 0.66–1.20 | 0.647 | 0.88 | 0.73–1.07 | 0.318 |
| Fathers education level (Bachelor's degree graduation) | 0.69 | 0.53–0.90 | **0.018** | 1.11 | 0.92–1.33 | 0.409 | 0.73 | 0.48–1.12 | 0.243 | 0.74 | 0.59–0.93 | **0.015** | 0.95 | 0.70–1.29 | 0.851 | 0.91 | 0.75–1.11 | 0.493 |
| Fathers education level (Master's/Ph.D./ Postdoctoral graduation) | 0.69 | 0.48–1.00 | 0.101 | 0.97 | 0.75–1.24 | 0.860 | 0.50 | 0.26–0.96 | 0.078 | 0.89 | 0.65–1.23 | 0.610 | 1.31 | 0.88–1.95 | 0.367 | 0.88 | 0.67–1.17 | 0.499 |
| Fathers education level (Not sure) | 0.73 | 0.53–1.01 | 0.108 | 0.88 | 0.70–1.10 | 0.408 | 0.83 | 0.49–1.40 | 0.576 | 0.82 | 0.62–1.08 | 0.230 | 0.82 | 0.56–1.21 | 0.550 | 0.99 | 0.77–1.26 | 0.987 |
| Fathers education level (Others [father's role missing, such as the father passing away, etc.]) | 0.69 | 0.52–0.92 | **0.027** | 0.97 | 0.79–1.18 | 0.860 | 0.73 | 0.46–1.14 | 0.263 | 0.66 | 0.52–0.84 | **0.002** | 0.96 | 0.69–1.34 | 0.860 | 1.00 | 0.80–1.24 | 0.992 |
| Mothers education level (Primary school graduation) | 0.90 | 0.75–1.08 | 0.445 | 0.86 | 0.77–0.96 | **0.015** | 0.91 | 0.68–1.22 | 0.611 | 0.89 | 0.77–1.04 | 0.199 | 0.88 | 0.72–1.07 | 0.402 | 0.87 | 0.77–0.98 | 0.053 |
| Mothers education level (Junior high school graduation) | 0.82 | 0.68–0.98 | 0.059 | 0.76 | 0.68–0.85 | **< 0.001** | 0.68 | 0.51–0.92 | **0.030** | 0.80 | 0.69–0.93 | **0.007** | 0.79 | 0.65–0.96 | **0.043** | 0.80 | 0.70–0.90 | **< 0.001** |
| Mothers education level (High school/Technical school graduation) | 0.90 | 0.75–1.09 | 0.445 | 0.78 | 0.69–0.87 | **< 0.001** | 0.75 | 0.56–1.03 | 0.145 | 0.81 | 0.69–0.95 | **0.015** | 0.83 | 0.68–1.02 | 0.164 | 0.74 | 0.65–0.83 | **< 0.001** |
| Mothers education level (Associate degree graduation) | 1.05 | 0.86–1.29 | 0.791 | 0.86 | 0.76–0.98 | **0.043** | 0.75 | 0.53–1.05 | 0.177 | 0.87 | 0.73–1.03 | 0.156 | 0.96 | 0.77–1.20 | 0.850 | 0.81 | 0.71–0.94 | **0.009** |
| Mothers education level (Bachelor's degree graduation) | 1.08 | 0.87–1.33 | 0.684 | 0.77 | 0.68–0.88 | **< 0.001** | 0.76 | 0.53–1.09 | 0.234 | 0.81 | 0.68–0.98 | **0.043** | 0.92 | 0.73–1.16 | 0.654 | 0.79 | 0.69–0.92 | **0.006** |
| Mothers education level (Master's/Ph.D./ Postdoctoral graduation) | 1.43 | 1.00–2.04 | 0.101 | 1.05 | 0.82–1.34 | 0.828 | 1.59 | 0.89–2.82 | 0.204 | 0.90 | 0.65–1.26 | 0.645 | 1.18 | 0.80–1.74 | 0.647 | 0.97 | 0.74–1.28 | 0.929 |
| Mothers education level (Not sure) | 1.02 | 0.77–1.36 | 0.987 | 0.84 | 0.69–1.01 | 0.106 | 0.56 | 0.34–0.92 | 0.060 | 0.90 | 0.70–1.14 | 0.469 | 0.77 | 0.56–1.07 | 0.268 | 0.64 | 0.52–0.79 | **< 0.001** |
| Mothers education level (Others [mother's role missing, such as the mother passing away, etc.]) | 1.01 | 0.77–1.33 | 0.994 | 1.03 | 0.86–1.24 | 0.828 | 0.96 | 0.62–1.48 | 0.911 | 1.06 | 0.84–1.33 | 0.704 | 0.90 | 0.66–1.22 | 0.654 | 0.93 | 0.76–1.13 | 0.551 |
| Household income￥6000–14 000 | 1.02 | 0.95–1.09 | 0.737 | 1.07 | 1.02–1.11 | **0.005** | 0.85 | 0.76–0.96 | **0.027** | 0.90 | 0.85–0.95 | **< 0.001** | 0.92 | 0.86–0.99 | 0.092 | 0.95 | 0.90–0.99 | **0.037** |
| Household income￥14 000–23 000 | 1.00 | 0.92–1.09 | 0.999 | 1.12 | 1.07–1.18 | **< 0.001** | 0.94 | 0.81–1.08 | 0.516 | 0.84 | 0.78–0.90 | **< 0.001** | 0.87 | 0.80–0.96 | **0.011** | 0.88 | 0.83–0.93 | **< 0.001** |
| Household income￥23 000–36 000 | 0.99 | 0.90–1.10 | 0.989 | 1.10 | 1.03–1.17 | **0.005** | 0.86 | 0.72–1.03 | 0.175 | 0.83 | 0.76–0.90 | **< 0.001** | 0.96 | 0.86–1.07 | 0.647 | 0.89 | 0.83–0.95 | **0.002** |
| Household income￥36 000–70 000 | 1.06 | 0.95–1.19 | 0.445 | 1.13 | 1.05–1.21 | **0.002** | 0.89 | 0.72–1.09 | 0.376 | 0.81 | 0.73–0.89 | **< 0.001** | 0.87 | 0.77–1.00 | 0.104 | 0.92 | 0.85–0.99 | 0.057 |
| Household income (＞￥70,000) | 0.97 | 0.85–1.11 | 0.808 | 0.99 | 0.91–1.08 | 0.860 | 0.99 | 0.79–1.25 | 1.000 | 0.79 | 0.70–0.89 | **< 0.001** | 1.05 | 0.92–1.21 | 0.649 | 0.91 | 0.83–1.00 | 0.096 |
| Drinking (≤1 time/month) | 1.59 | 1.50–1.69 | **< 0.001** | 1.46 | 1.41–1.51 | **< 0.001** | 1.32 | 1.18–1.48 | **< 0.001** | 1.36 | 1.29–1.44 | **< 0.001** | 1.17 | 1.09–1.25 | **< 0.001** | 1.38 | 1.33–1.44 | **< 0.001** |
| Drinking 2–4 times/month | 2.57 | 2.36–2.80 | **< 0.001** | 1.81 | 1.71–1.92 | **< 0.001** | 2.18 | 1.88–2.54 | **< 0.001** | 1.96 | 1.82–2.11 | **< 0.001** | 1.64 | 1.49–1.81 | **< 0.001** | 1.91 | 1.80–2.03 | **< 0.001** |
| Drinking 2–3 times/week | 3.21 | 2.76–3.73 | **< 0.001** | 2.15 | 1.91–2.41 | **< 0.001** | 3.33 | 2.61–4.24 | **< 0.001** | 3.01 | 2.63–3.43 | **< 0.001** | 2.72 | 2.31–3.20 | **< 0.001** | 2.63 | 2.34–2.95 | **< 0.001** |
| Drinking (≥4 times/week) | 3.70 | 3.06–4.48 | **< 0.001** | 2.07 | 1.78–2.42 | **< 0.001** | 4.44 | 3.35–5.89 | **< 0.001** | 3.23 | 2.72–3.84 | **< 0.001** | 3.08 | 2.51–3.78 | **< 0.001** | 3.14 | 2.70–3.66 | **< 0.001** |
| Smoking (Yes) | 1.83 | 1.69–1.97 | **< 0.001** | 1.16 | 1.10–1.22 | **< 0.001** | 1.86 | 1.62–2.12 | **< 0.001** | 1.67 | 1.57–1.79 | **< 0.001** | 1.44 | 1.32–1.57 | **< 0.001** | 1.51 | 1.43–1.60 | **< 0.001** |
| Smooth term (Exercise Duration2) × Exercise Typedouble swing |  |  | **0.032** |  |  | **< 0.001** |  |  | 0.078 |  |  | **< 0.001** |  |  | **< 0.001** |  |  | **< 0.001** |
| Smooth term (Exercise Duration2) × Exercise Typelow intensity aerobics |  |  | **0.003** |  |  | **< 0.001** |  |  | 0.078 |  |  | **< 0.001** |  |  | **< 0.001** |  |  | **< 0.001** |
| Smooth term (Exercise Duration2) × Exercise Typemedium intensity aerobics |  |  | **< 0.001** |  |  | **< 0.001** |  |  | **0.014** |  |  | **< 0.001** |  |  | **< 0.001** |  |  | **< 0.001** |
| Smooth term (Exercise Duration2) × Exercise Typesingle anaerobics |  |  | **< 0.001** |  |  | **< 0.001** |  |  | 0.060 |  |  | **< 0.001** |  |  | **< 0.001** |  |  | **< 0.001** |
| Smooth term (Exercise Duration2) × Exercise Typeteam ball |  |  | **< 0.001** |  |  | **< 0.001** |  |  | **0.001** |  |  | **< 0.001** |  |  | **< 0.001** |  |  | **< 0.001** |
| Observations | 79011 | | | 79 011 | | | 79 011 | | | 79 011 | | | 79 011 | | | 79 011 | | |
| R^2^ | 0.041 | | | 0.048 | | | 0.019 | | | 0.038 | | | 0.022 | | | 0.030 | | |
| AIC | 42541.393 | | | 89085.266 | | | 16833.521 | | | 52927.093 | | | 35842.355 | | | 75130.868 | | |

Abbreviations: CI, confidence interval; FDR, false discovery rate; NSSI, non-suicidal self-injury; PTSD, post-traumatic stress disorder; SA, suicide attempt; SI, suicidal ideation.

**S Table 10.** Logistic regression results of exercise types on various mental health outcomes: Female Group (N=45 432).

|  | **Lifetime NSSI** | | | **Lifetime SI** | | | **Lifetime SA** | | | **Depression** | | | **Anxiety** | | | **PTSD** | | |
| --- | --- | --- | --- | --- | --- | --- | --- | --- | --- | --- | --- | --- | --- | --- | --- | --- | --- | --- |
| ***Predictors*** | ***Odds ratios*** | ***CI (95%)*** | ***P value (fdr)*** | ***Odds ratios*** | ***CI (95%)*** | ***P value (fdr)*** | ***Odds ratios*** | ***CI (95%)*** | ***P value (fdr)*** | ***Odds ratios*** | ***CI (95%)*** | ***P value (fdr)*** | ***Odds ratios*** | ***CI (95%)*** | ***P value (fdr)*** | ***Odds ratios*** | ***CI (95%)*** | ***P value (fdr)*** |
| **Reference: team ball**  **Reference** | | | | | | | | | | | | | | | | | | |
| double swing | 0.86 | 0.77–0.97 | **0.036** | 1.04 | 0.97–1.12 | 0.265 | 0.91 | 0.74–1.13 | 0.503 | 0.95 | 0.85–1.05 | 0.515 | 0.95 | 0.83–1.10 | 0.507 | 1.01 | 0.93–1.10 | 0.830 |
| low intensity aerobics | 1.11 | 0.99–1.25 | 0.126 | 1.26 | 1.17–1.36 | **< 0.001** | 1.09 | 0.89–1.34 | 0.503 | 1.20 | 1.08–1.33 | **0.002** | 1.26 | 1.10–1.45 | **0.002** | 1.11 | 1.01–1.21 | 0.055 |
| medium intensity aerobics | 1.07 | 0.95–1.21 | 0.343 | 1.28 | 1.19–1.39 | **< 0.001** | 1.16 | 0.94–1.44 | 0.404 | 1.04 | 0.93–1.16 | 0.631 | 1.17 | 1.01–1.35 | 0.065 | 1.10 | 1.01–1.21 | 0.063 |
| single anaerobics | 0.96 | 0.84–1.09 | 0.501 | 1.05 | 0.97–1.14 | 0.258 | 1.01 | 0.80–1.26 | 0.959 | 1.00 | 0.89–1.13 | 0.960 | 1.09 | 0.93–1.26 | 0.359 | 1.06 | 0.97–1.17 | 0.256 |
| **Reference: two-player racket**  **Reference** | | | | | | | | | | | | | | | | | | |
| low intensity aerobics | 1.29 | 1.18–1.41 | **<0 .001** | 1.21 | 1.15–1.28 | **< 0.001** | 1.20 | 1.02–1.40 | **0.040** | 1.27 | 1.17–1.37 | **< 0.001** | 1.33 | 1.20–1.47 | **< 0.001** | 1.10 | 1.03–1.17 | **0.011** |
| medium intensity aerobics | 1.24 | 1.13–1.37 | **< 0.001** | 1.23 | 1.16–1.30 | **< 0.001** | 1.27 | 1.08–1.50 | **0.010** | 1.10 | 1.01–1.20 | 0.057 | 1.22 | 1.10–1.37 | **<0 .001** | 1.09 | 1.02–1.17 | **0.023** |
| single anaerobics | 1.11 | 1.00–1.23 | **0.047** | 1.01 | 0.95–1.08 | 0.760 | 1.10 | 0.92–1.31 | 0.371 | 1.06 | 0.97–1.16 | 0.258 | 1.14 | 1.01–1.28 | **0.038** | 1.05 | 0.98–1.13 | 0.210 |
| team ball | 1.16 | 1.03–1.31 | **0.018** | 0.96 | 0.89–1.03 | 0.332 | 1.09 | 0.89–1.35 | 0.403 | 1.06 | 0.95–1.18 | 0.309 | 1.05 | 0.91–1.21 | 0.507 | 0.99 | 0.91–1.08 | 0.830 |
| **Reference: low intensity aerobics**  **Reference** | | | | | | | | | | | | | | | | | | |
| double swing | 0.78 | 0.71–0.85 | **< 0.001** | 0.82 | 0.78–0.87 | **< 0.001** | 0.84 | 0.72–0.98 | 0.060 | 0.79 | 0.73–0.85 | **< 0.001** | 0.75 | 0.68–0.83 | **< 0.001** | 0.91 | 0.86–0.97 | **0.011** |
| medium intensity aerobics | 0.96 | 0.88–1.06 | 0.430 | 1.01 | 0.96–1.08 | 0.626 | 1.06 | 0.91–1.25 | 0.450 | 0.87 | 0.80–0.94 | **<0 .001** | 0.92 | 0.83–1.03 | 0.145 | 1.00 | 0.93–1.07 | 0.888 |
| single anaerobics | 0.86 | 0.78–0.95 | **0.006** | 0.83 | 0.78–0.89 | **< 0.001** | 0.92 | 0.77–1.10 | 0.450 | 0.84 | 0.76–0.92 | **< 0.001** | 0.86 | 0.77–0.96 | **0.011** | 0.96 | 0.89–1.03 | 0.334 |
| team ball | 0.90 | 0.80–1.01 | 0.094 | 0.79 | 0.73–0.85 | **< 0.001** | 0.91 | 0.74–1.12 | 0.450 | 0.83 | 0.75–0.93 | **<0 .001** | 0.79 | 0.69–0.91 | **0.002** | 0.90 | 0.83–0.99 | **0.036** |
| **Reference: medium intensity aerobics** | | | | | | | | | | | | | | | | | | |
| double swing | 0.81 | 0.73–0.89 | **< 0.001** | 0.81 | 0.77–0.86 | **< 0.001** | 0.79 | 0.67–0.93 | **0.010** | 0.91 | 0.83–0.99 | 0.057 | 0.82 | 0.73–0.91 | **<0 .001** | 0.92 | 0.86–0.98 | **0.034** |
| low intensity aerobics | 1.04 | 0.95–1.14 | 0.430 | 0.99 | 0.93–1.04 | 0.626 | 0.94 | 0.80–1.10 | 0.450 | 1.15 | 1.06–1.26 | **0.003** | 1.08 | 0.97–1.21 | 0.181 | 1.00 | 0.94–1.08 | 0.888 |
| single anaerobics | 0.89 | 0.80–1.00 | 0.067 | 0.82 | 0.77–0.88 | **< 0.001** | 0.86 | 0.72–1.04 | 0.197 | 0.96 | 0.87–1.06 | 0.505 | 0.93 | 0.82–1.05 | 0.252 | 0.96 | 0.89–1.04 | 0.452 |
| team ball | 0.93 | 0.83–1.06 | 0.343 | 0.78 | 0.72–0.84 | **< 0.001** | 0.86 | 0.69–1.06 | 0.202 | 0.96 | 0.86–1.08 | 0.505 | 0.86 | 0.74–0.99 | 0.065 | 0.91 | 0.83–0.99 | 0.063 |
| **Reference: single anaerobic**  **Reference** | | | | | | | | | | | | | | | | | | |
| double swing | 0.90 | 0.81–1.00 | 0.059 | 0.99 | 0.93–1.05 | 0.760 | 0.91 | 0.76–1.09 | 0.432 | 0.94 | 0.86–1.03 | 0.345 | 0.88 | 0.78–0.99 | 0.051 | 0.95 | 0.88–1.02 | 0.334 |
| low intensity aerobics | 1.16 | 1.05–1.28 | **0.009** | 1.20 | 1.13–1.28 | **< 0.001** | 1.09 | 0.91–1.30 | 0.432 | 1.20 | 1.09–1.31 | **< 0.001** | 1.16 | 1.04–1.31 | **0.023** | 1.04 | 0.97–1.12 | 0.334 |
| medium intensity aerobics | 1.12 | 1.01–1.24 | 0.059 | 1.22 | 1.14–1.30 | **< 0.001** | 1.16 | 0.96–1.39 | 0.295 | 1.04 | 0.94–1.14 | 0.595 | 1.07 | 0.95–1.22 | 0.287 | 1.04 | 0.96–1.12 | 0.362 |
| team ball | 1.04 | 0.92–1.19 | 0.501 | 0.95 | 0.88–1.03 | 0.258 | 0.99 | 0.80–1.24 | 0.959 | 1.00 | 0.89–1.12 | 0.960 | 0.92 | 0.79–1.07 | 0.287 | 0.94 | 0.86–1.03 | 0.334 |

Note: Propensity scores for the five exercise types were included as weights, and several covariates were included in the model. These covariates included age, gender identity, ethnicity, only-child status, education level, smoking status and alcohol consumption, family structure, household income, parental education level, and BMI.

Abbreviations: CI, confidence interval; FDR, false discovery rate; NSSI, non-suicidal self-injury; PTSD, post-traumatic stress disorder; SA, suicide attempt; SI, suicidal ideation.

**S Table 11.** Logistic regression results of exercise types on various mental health outcomes:Male Group (N=33 579).

|  | **Lifetime NSSI** | | | **Lifetime SI** | | | **Lifetime SA** | | | **Depression** | | | **Anxiety** | | | **PTSD** | | |
| --- | --- | --- | --- | --- | --- | --- | --- | --- | --- | --- | --- | --- | --- | --- | --- | --- | --- | --- |
| ***Predictors*** | ***Odds ratios*** | ***CI (95%)*** | ***p.adjusted (fdr)*** | ***Odds ratios*** | ***CI (95%)*** | ***p.adjusted (fdr)*** | ***Odds ratios*** | ***CI (95%)*** | ***p.adjusted (fdr)*** | ***Odds ratios*** | ***CI (95%)*** | ***p.adjusted (fdr)*** | ***Odds ratios*** | ***CI (95%)*** | ***p.adjusted (fdr)*** | ***Odds ratios*** | ***CI (95%)*** | ***p.adjusted (fdr)*** |
| **Reference: team ball**  **Reference** | | | | | | | | | | | | | | | | | | |
| double swing | 1.26 | 1.12–1.43 | **< 0.001** | 1.55 | 1.44–1.67 | **< 0.001** | 1.29 | 1.01–1.65 | **0.041** | 1.38 | 1.25–1.53 | **< 0.001** | 1.30 | 1.13–1.49 | **< 0.001** | 1.15 | 1.06–1.24 | **<0 .001** |
| low intensity aerobics | 1.74 | 1.49–2.04 | **< 0.001** | 2.26 | 2.05–2.49 | **< 0.001** | 2.18 | 1.63–2.91 | **< 0.001** | 2.13 | 1.88–2.41 | **< 0.001** | 2.16 | 1.83–2.54 | **< 0.001** | 1.40 | 1.26–1.56 | **< 0.001** |
| medium intensity aerobics | 1.53 | 1.34–1.75 | **< 0.001** | 1.73 | 1.59–1.88 | **< 0.001** | 1.68 | 1.29–2.19 | **< 0.001** | 1.61 | 1.44–1.81 | **< 0.001** | 1.69 | 1.46–1.96 | **< 0.001** | 1.23 | 1.12–1.35 | **< 0.001** |
| single anaerobics | 1.34 | 1.19–1.50 | **< 0.001** | 1.24 | 1.15–1.33 | **< 0.001** | 1.45 | 1.15–1.82 | **0.002** | 1.31 | 1.19–1.44 | **< 0.001** | 1.37 | 1.20–1.56 | **< 0.001** | 1.10 | 1.02–1.18 | **0.013** |
| **Reference: two-player racket**  **Reference** | | | | | | | | | | | | | | | | | | |
| low intensity aerobics | 1.38 | 1.17–1.62 | **< 0.001** | 1.45 | 1.32–1.60 | **< 0.001** | 1.69 | 1.26–2.27 | **<0 .001** | 1.54 | 1.35–1.75 | **< 0.001** | 1.66 | 1.40–1.96 | **< 0.001** | 1.22 | 1.09–1.36 | **<0 .001** |
| medium intensity aerobics | 1.21 | 1.05–1.39 | **0.010** | 1.11 | 1.02–1.21 | **0.016** | 1.30 | 0.99–1.71 | 0.074 | 1.17 | 1.04–1.31 | **0.010** | 1.30 | 1.12–1.52 | **<0 .001** | 1.07 | 0.97–1.18 | 0.193 |
| single anaerobics | 1.06 | 0.94–1.19 | 0.373 | 0.80 | 0.74–0.86 | **< 0.001** | 1.12 | 0.88–1.42 | 0.343 | 0.95 | 0.86–1.05 | 0.303 | 1.05 | 0.92–1.20 | 0.438 | 0.96 | 0.88–1.04 | 0.272 |
| team ball | 0.79 | 0.70–0.89 | **< 0.001** | 0.64 | 0.60–0.69 | **< 0.001** | 0.77 | 0.61–0.99 | 0.069 | 0.72 | 0.65–0.80 | **< 0.001** | 0.77 | 0.67–0.88 | **< 0.001** | 0.87 | 0.81–0.94 | **<0 .001** |
| **Reference: low intensity aerobics**  **Reference** | | | | | | | | | | | | | | | | | | |
| double swing | 0.73 | 0.62–0.85 | **< 0.001** | 0.69 | 0.62–0.76 | **< 0.001** | 0.59 | 0.44–0.80 | **<0 .001** | 0.65 | 0.57–0.74 | **< 0.001** | 0.60 | 0.51–0.71 | **< 0.001** | 0.82 | 0.73–0.92 | **<0 .001** |
| medium intensity aerobics | 0.88 | 0.74–1.04 | 0.140 | 0.77 | 0.69–0.85 | **< 0.001** | 0.77 | 0.56–1.05 | 0.104 | 0.76 | 0.66–0.87 | **< 0.001** | 0.78 | 0.66–0.94 | **0.008** | 0.88 | 0.78–0.99 | **0.034** |
| single anaerobics | 0.77 | 0.65–0.90 | **<0 .001** | 0.55 | 0.50–0.60 | **< 0.001** | 0.66 | 0.50–0.88 | **0.006** | 0.62 | 0.54–0.70 | **< 0.001** | 0.63 | 0.54–0.75 | **< 0.001** | 0.78 | 0.70–0.87 | **< 0.001** |
| team ball | 0.57 | 0.49–0.67 | **< 0.001** | 0.44 | 0.40–0.49 | **< 0.001** | 0.46 | 0.34–0.61 | **< 0.001** | 0.47 | 0.41–0.53 | **< 0.001** | 0.46 | 0.39–0.55 | **< 0.001** | 0.71 | 0.64–0.79 | **< 0.001** |
| **Reference: medium intensity aerobics** | | | | | | | | | | | | | | | | | | |
| double swing | 0.83 | 0.72–0.95 | **0.013** | 0.90 | 0.82–0.98 | **0.016** | 0.77 | 0.58–1.01 | 0.099 | 0.86 | 0.76–0.96 | **0.008** | 0.77 | 0.66–0.89 | **<0 .001** | 0.93 | 0.85–1.03 | 0.155 |
| low intensity aerobics | 1.14 | 0.96–1.35 | 0.140 | 1.31 | 1.17–1.45 | **< 0.001** | 1.30 | 0.95–1.77 | 0.130 | 1.32 | 1.15–1.51 | **< 0.001** | 1.27 | 1.07–1.52 | **0.008** | 1.14 | 1.01–1.28 | **0.043** |
| single anaerobics | 0.87 | 0.76–1.00 | 0.059 | 0.72 | 0.66–0.78 | **< 0.001** | 0.86 | 0.67–1.12 | 0.258 | 0.81 | 0.73–0.91 | **< 0.001** | 0.81 | 0.70–0.94 | **0.005** | 0.89 | 0.81–0.98 | **0.026** |
| team ball | 0.65 | 0.57–0.75 | **< 0.001** | 0.58 | 0.53–0.63 | **< 0.001** | 0.59 | 0.46–0.78 | **< 0.001** | 0.62 | 0.55–0.69 | **< 0.001** | 0.59 | 0.51–0.68 | **< 0.001** | 0.81 | 0.74–0.89 | **< 0.001** |
| **Reference: single anaerobic**  **Reference** | | | | | | | | | | | | | | | | | | |
| double swing | 0.95 | 0.84–1.07 | 0.373 | 1.25 | 1.16–1.35 | **< 0.001** | 0.89 | 0.70–1.13 | 0.343 | 1.05 | 0.95–1.16 | 0.303 | 0.95 | 0.83–1.08 | 0.438 | 1.05 | 0.97–1.13 | 0.272 |
| low intensity aerobics | 1.31 | 1.12–1.53 | **<0 .001** | 1.82 | 1.65–2.01 | **< 0.001** | 1.50 | 1.14–1.99 | **0.007** | 1.62 | 1.43–1.84 | **< 0.001** | 1.58 | 1.34–1.85 | **< 0.001** | 1.28 | 1.14–1.42 | **< 0.001** |
| medium intensity aerobics | 1.15 | 1.00–1.31 | 0.059 | 1.40 | 1.28–1.52 | **< 0.001** | 1.16 | 0.90–1.50 | 0.322 | 1.23 | 1.10–1.38 | **< 0.001** | 1.24 | 1.07–1.43 | **0.005** | 1.12 | 1.02–1.23 | **0.020** |
| team ball | 0.75 | 0.67–0.84 | **< 0.001** | 0.81 | 0.75–0.87 | **< 0.001** | 0.69 | 0.55–0.87 | **0.004** | 0.76 | 0.69–0.84 | **< 0.001** | 0.73 | 0.64–0.83 | **< 0.001** | 0.91 | 0.85–0.98 | **0.020** |

Note: Propensity scores for the five exercise types were included as weights, and several covariates were included in the model. These covariates included age, gender identity, ethnicity, only-child status, education level, smoking status and alcohol consumption, family structure, household income, parental education level, and BMI.

Abbreviations: CI, confidence interval; FDR, false discovery rate; NSSI, non-suicidal self-injury; PTSD, post-traumatic stress disorder; SA, suicide attempt; SI, suicidal ideation.

**S Table 12** Smooth terms influencing exercise level as determined by logistic generalised additive modeling: Female Group (N=45 432).

|  | **NSSI** | | | **SI** | | | **SA** | | | **Depression** | | | **Anxiety** | | | **PTSD** | | |
| --- | --- | --- | --- | --- | --- | --- | --- | --- | --- | --- | --- | --- | --- | --- | --- | --- | --- | --- |
| ***Predictors*** | ***edf*** | ***Chi.sq*** | ***p*** | ***edf*** | ***Chi.sq*** | ***p*** | ***edf*** | ***Chi.sq*** | ***p*** | ***edf*** | ***Chi.sq*** | ***p*** | ***edf*** | ***Chi.sq*** | ***p*** | ***edf*** | ***Chi.sq*** | ***p*** |
| **Frequency** |  |  |  |  |  |  |  |  |  |  |  |  |  |  |  |  |  |  |
| Two-player racket | 2.218 | 12.519 | ***0.009*** | 2.391 | 53.613 | **<0 .001** | 1.588 | 1.161 | 0.484 | 2.545 | 72.551 | **<0 .001** | 2.293 | 28.887 | **<0 .001** | 2.109 | 23.608 | **<0 .001** |
| Low intensity aerobics | 1.708 | 1.976 | 0.403 | 2.115 | 12.326 | ***0.005*** | 1.586 | 3.251 | 0.25 | 2.555 | 61.921 | **<0 .001** | 2.323 | 24.703 | **<0 .001** | 2.54 | 62.9 | **<0 .001** |
| Medium intensity aerobics | 1 | 0.173 | 0.677 | 2.865 | 38.272 | **<0 .001** | 1.859 | 3.4 | 0.238 | 2.878 | 37.098 | **<0 .001** | 2.779 | 37.698 | **<0 .001** | 1.894 | 5.754 | 0.091 |
| Single anaerobics | 1.15 | 0.868 | 0.531 | 2.768 | 46.293 | **<0 .001** | 2.683 | 11.354 | ***0.006*** | 2.635 | 41.825 | **<0 .001** | 2.589 | 14.905 | ***0.004*** | 2.616 | 19.033 | **<0 .001** |
| Team ball | 1.006 | 2.806 | 0.095 | 2.33 | 21.563 | **<0 .001** | 1.005 | 3.93 | ***0.048*** | 2.097 | 28.549 | **<0 .001** | 1.608 | 23.459 | **<0 .001** | 2.11 | 14.761 | ***0.002*** |
| **Duration** |  |  |  |  |  |  |  |  |  |  |  |  |  |  |  |  |  |  |
| Two-player racket | 2.486 | 6.983 | 0.124 | 1.79 | 11.13 | ***0.006*** | 2.011 | 7.871 | ***0.042*** | 2.56 | 46.101 | **<0 .001** | 2.288 | 33.039 | **<0 .001** | 1.804 | 22.689 | **<0 .001** |
| Low intensity aerobics | 2.297 | 5.677 | 0.061 | 1.991 | 30.052 | **<0 .001** | 2.091 | 8.877 | ***0.03*** | 2.245 | 65.151 | **<0 .001** | 2.342 | 25.933 | **<0 .001** | 2.314 | 24.091 | **<0 .001** |
| Medium intensity aerobics | 1.009 | 14.639 | **<0 .001** | 1.662 | 54.279 | **<0 .001** | 1.891 | 8.512 | ***0.025*** | 2.121 | 45.412 | **<0 .001** | 2.12 | 20.953 | **<0 .001** | 1.001 | 11.28 | **<0 .001** |
| Single anaerobics | 1.887 | 8.169 | ***0.028*** | 1.839 | 29.985 | **<0 .001** | 2.06 | 6.906 | 0.074 | 2.077 | 40.086 | **<0 .001** | 2.2 | 13.454 | ***0.006*** | 1.686 | 22.829 | **<0 .001** |
| Team ball | 1.001 | 1.557 | 0.213 | 1.009 | 12.158 | **<0 .001** | 1.01 | 3.752 | 0.055 | 2.094 | 19.229 | **<0 .001** | 1.907 | 12.743 | ***0.003*** | 1.582 | 9.33 | ***0.017*** |

Note: ^a^ The smooth terms included in the generalised additive model are summarised by their estimated degrees of freedom (edf).

Abbreviations: Chi.sq, chi-squared test statistic; Edf, estimated degrees of freedom; GAM, generalised additive model; NSSI, non-suicidal self-injury; PTSD, post-traumatic stress disorder; SA, suicide attempt; SI, suicidal ideation.

Significant values are in *italic* and **bold**. These covariates included age, gender identity, ethnicity, only-child status, education level, smoking status and alcohol consumption, family structure, household income, parental education level, and BMI.

**S Table 13** Smooth terms influencing exercise level as determined by logistic generalised additive modeling: Male Group (N=33 579).

|  | **NSSI** | | | **SI** | | | **SA** | | | **Depression** | | | **Anxiety** | | | **PTSD** | | |
| --- | --- | --- | --- | --- | --- | --- | --- | --- | --- | --- | --- | --- | --- | --- | --- | --- | --- | --- |
| ***Predictors*** | ***edf*** | ***Chi.sq*** | ***p*** | ***edf*** | ***Chi.sq*** | ***p*** | ***edf*** | ***Chi.sq*** | ***p*** | ***edf*** | ***Chi.sq*** | ***p*** | ***edf*** | ***Chi.sq*** | ***p*** | ***edf*** | ***Chi.sq*** | ***p*** |
| **Frequency** |  |  |  |  |  |  |  |  |  |  |  |  |  |  |  |  |  |  |
| Two-player racket | 2.209 | 23.127 | **<0 .001** | 2.509 | 128.584 | **<0 .001** | 2.052 | 14.518 | ***0.002*** | 2.439 | 126.444 | **<0 .001** | 2.408 | 70.65 | **<0 .001** | 2.169 | 66.598 | **<0 .001** |
| Low intensity aerobics | 1.003 | 3.368 | 0.067 | 1.839 | 52.612 | **<0 .001** | 1.824 | 4.068 | 0.145 | 2.39 | 36.456 | **<0 .001** | 2.097 | 12.221 | ***0.003*** | 1.035 | 9.508 | ***0.003*** |
| Medium intensity aerobics | 1.254 | 0.501 | 0.768 | 2.321 | 10.009 | ***0.025*** | 1.003 | 3.826 | 0.051 | 2.627 | 43.868 | **<0 .001** | 2.029 | 12.152 | ***0.005*** | 2.023 | 25.915 | **<0 .001** |
| Single anaerobics | 1.727 | 7.204 | ***0.03*** | 2.548 | 27.638 | **<0 .001** | 1.003 | 0.338 | 0.564 | 2.367 | 64.955 | **<0 .001** | 2.002 | 37.8 | **<0 .001** | 1.96 | 14.09 | ***0.002*** |
| Team ball | 2.434 | 48.085 | **<0 .001** | 2.498 | 139.414 | **<0 .001** | 1.003 | 6.356 | ***0.012*** | 2.739 | 135.542 | **<0 .001** | 2.876 | 89.107 | **<0 .001** | 2.455 | 73.198 | **<0 .001** |
| **Duration** |  |  |  |  |  |  |  |  |  |  |  |  |  |  |  |  |  |  |
| Two-player racket | 1.062 | 12.019 | **<0 .001** | 1.01 | 47.856 | **<0 .001** | 1.009 | 0.166 | 0.688 | 2.252 | 67.422 | **<0 .001** | 2.371 | 51.33 | **<0 .001** | 2.103 | 47.835 | **<0 .001** |
| Low intensity aerobics | 1.047 | 25.655 | **<0 .001** | 2.839 | 137.378 | **<0 .001** | 1.004 | 3.707 | 0.055 | 2.369 | 73.292 | **<0 .001** | 1.926 | 57.296 | **<0 .001** | 1.001 | 34.833 | **<0 .001** |
| Medium intensity aerobics | 2.602 | 18.914 | **<0 .001** | 2.079 | 91.297 | **<0 .001** | 1.848 | 2.701 | 0.247 | 2.428 | 38.943 | **<0 .001** | 2.118 | 12.022 | ***0.004*** | 1.721 | 23.327 | **<0 .001** |
| Single anaerobics | 1.699 | 26.859 | **<0 .001** | 2.719 | 100.783 | **<0 .001** | 1.001 | 3.192 | 0.074 | 2.595 | 57.911 | **<0 .001** | 2.577 | 43.133 | **<0 .001** | 1.395 | 25.94 | **<0 .001** |
| Team ball | 1.062 | 12.019 | **<0 .001** | 1.01 | 47.856 | **<0 .001** | 1.009 | 0.166 | 0.688 | 2.252 | 67.422 | **<0 .001** | 2.371 | 51.33 | **<0 .001** | 2.103 | 47.835 | **<0 .001** |

Note: ^a^ The smooth terms included in the generalised additive model are summarised by their estimated degrees of freedom (edf).

Abbreviations: Chi.sq, chi-squared test statistic; Edf, estimated degrees of freedom; GAM, generalised additive model; NSSI, non-suicidal self-injury; PTSD, post-traumatic stress disorder; SA, suicide attempt; SI, suicidal ideation.

Significant values are in *italic* and **bold**. These covariates included age, gender identity, ethnicity, only-child status, education level, smoking status and alcohol consumption, family structure, household income, parental education level, and BMI.


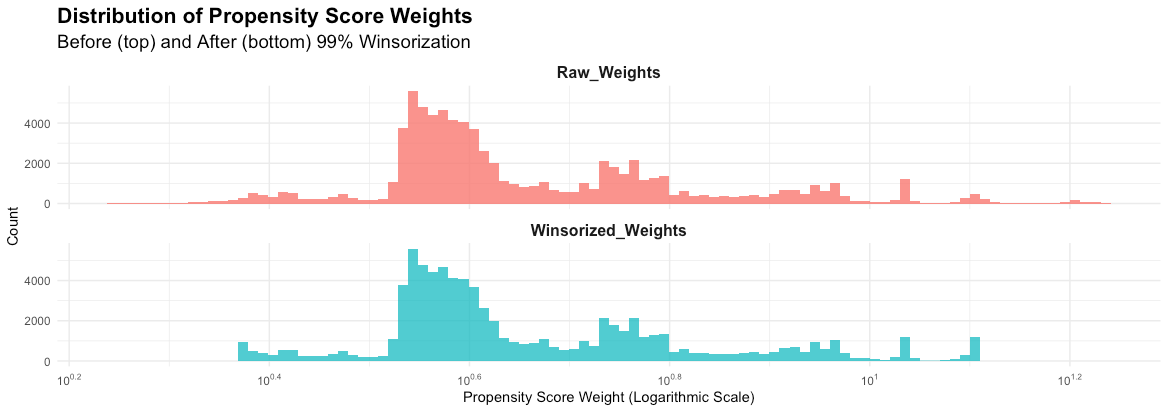


**S Fig1. The weight distribution before and after winsorization.**


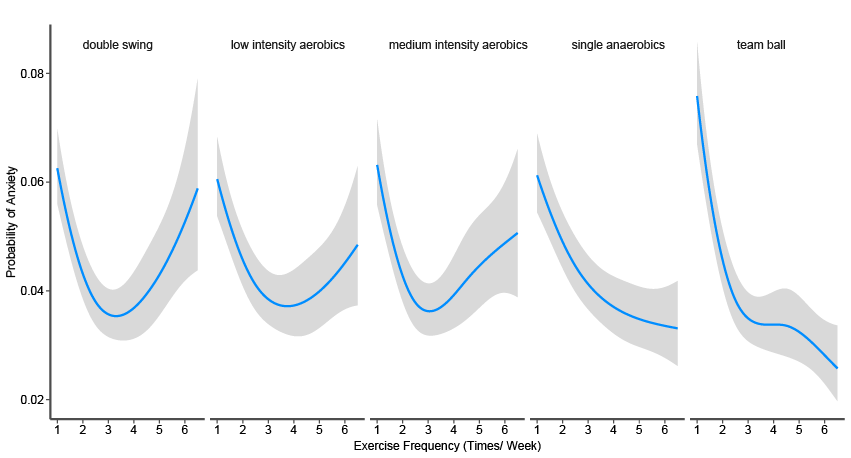


**S Fig2. Anxiety symptom as a function of exercise frequency.** The lines in the figure represent smooth conditional means using generalised additive model smoothing with cubic regression splines, whereas the ribbons represent 95% confidence intervals.


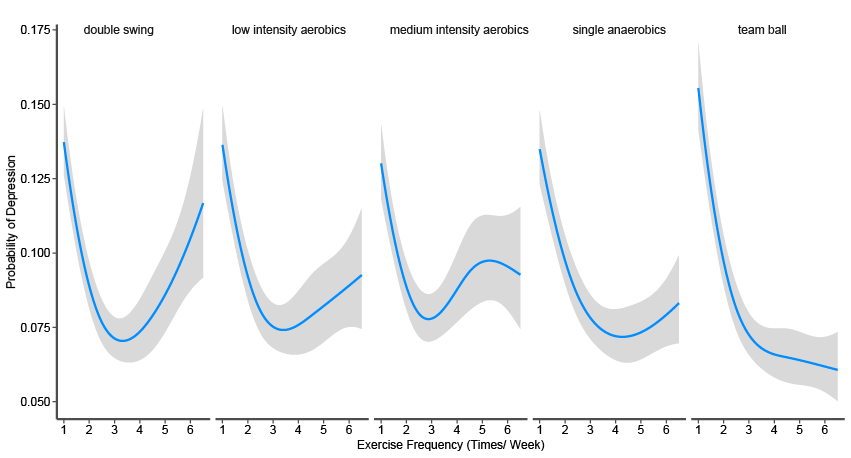


**S Fig3. Depression symptom as a function of exercise frequency.** The lines in the figure represent smooth conditional means using generalised additive model smoothing with cubic regression splines, whereas the ribbons represent 95% confidence intervals.


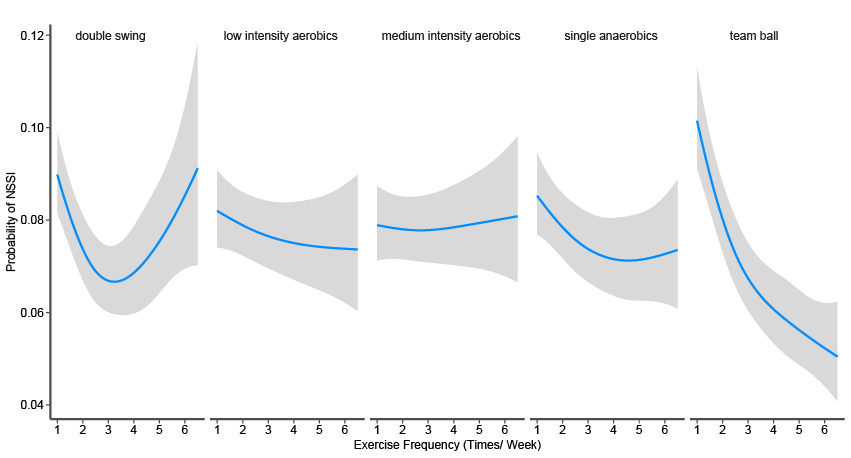


**S Fig4. NSSI as a function of exercise frequency.** The lines in the figure represent smooth conditional means using generalised additive model smoothing with cubic regression splines, whereas the ribbons represent 95% confidence intervals.


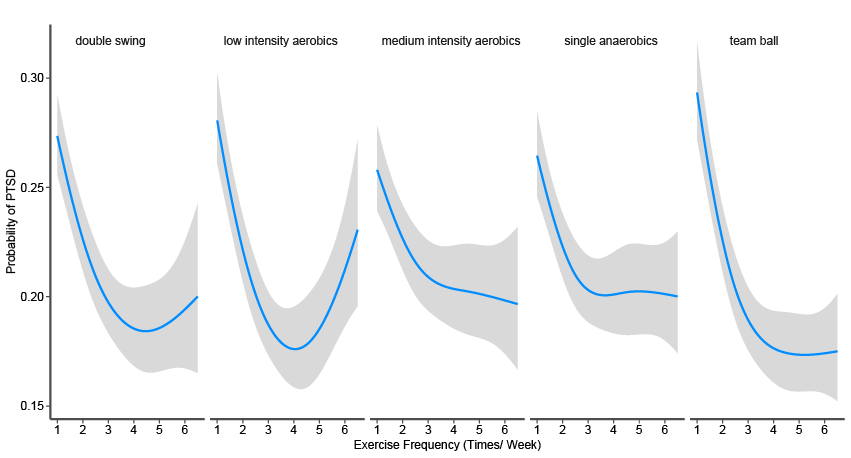


**S Fig5. PTSD as a function of exercise frequency.** The lines in the figure represent smooth conditional means using generalised additive model smoothing with cubic regression splines, whereas the ribbons represent 95% confidence intervals.


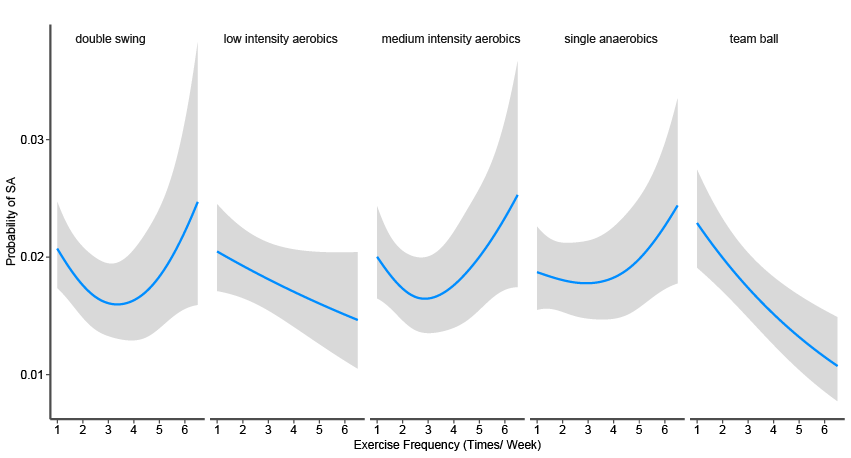


**S Fig6. SA as a function of exercise frequency.** The lines in the figure represent smooth conditional means using generalised additive model smoothing with cubic regression splines, whereas the ribbons represent 95% confidence intervals.


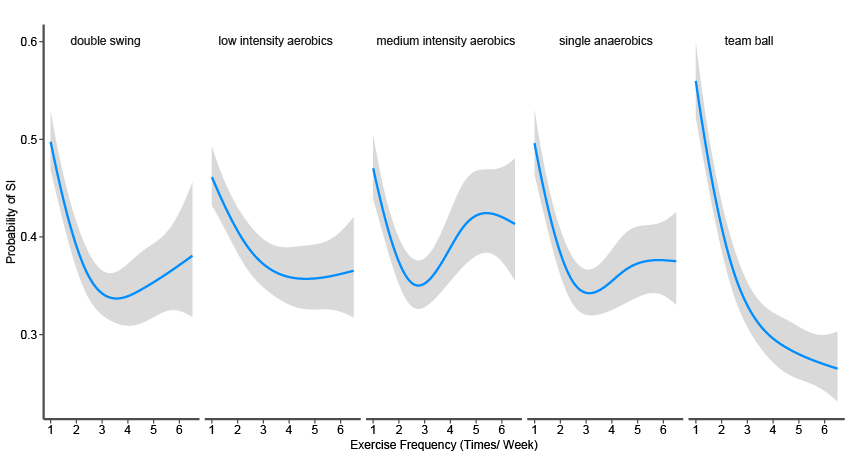


**S Fig7. SI as a function of exercise frequency.** The lines in the figure represent smooth conditional means using generalised additive model smoothing with cubic regression splines, whereas the ribbons represent 95% confidence intervals.


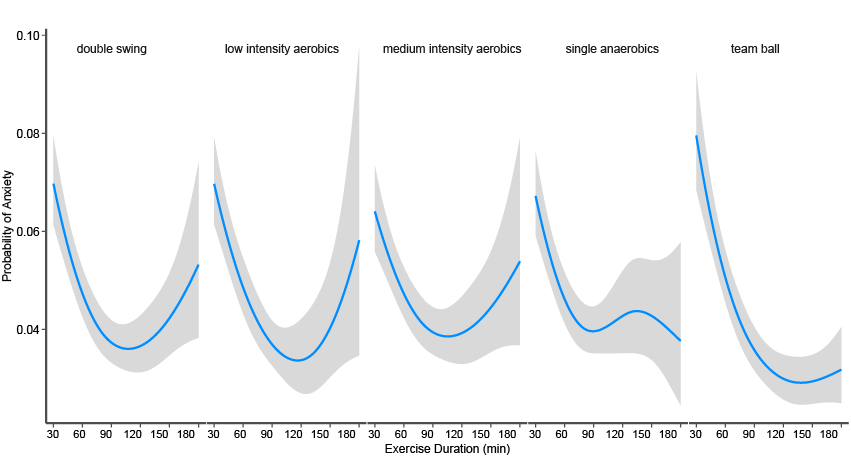


**S Fig8. Anxiety symptom as a function of exercise duration.** The lines in the figure represent smooth conditional means using generalised additive model smoothing with cubic regression splines, whereas the ribbons represent 95% confidence intervals.


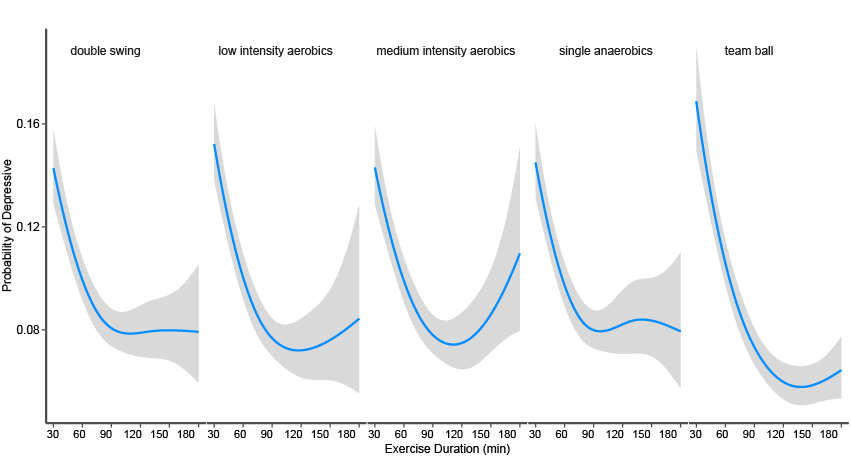


**S Fig9. Depression symptom as a function of exercise duration.** The lines in the figure represent smooth conditional means using generalised additive model smoothing with cubic regression splines, whereas the ribbons represent 95% confidence intervals.


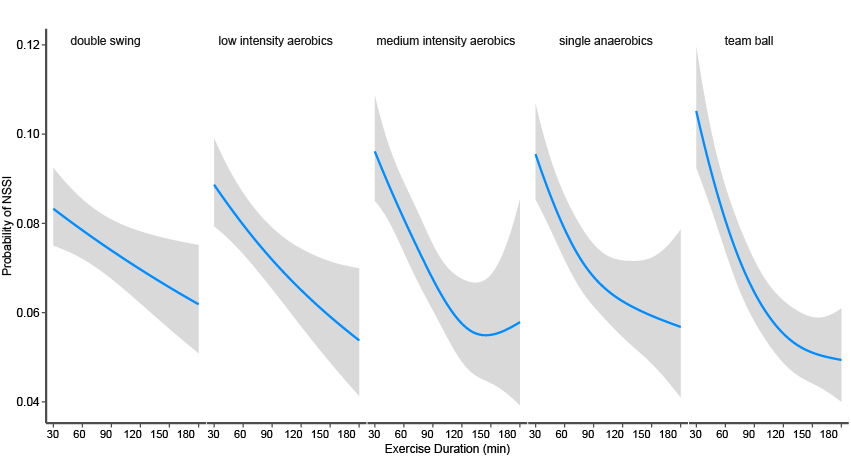


**S Fig10. NSSI as a function of exercise duration.** The lines in the figure represent smooth conditional means using generalised additive model smoothing with cubic regression splines, whereas the ribbons represent 95% confidence intervals.


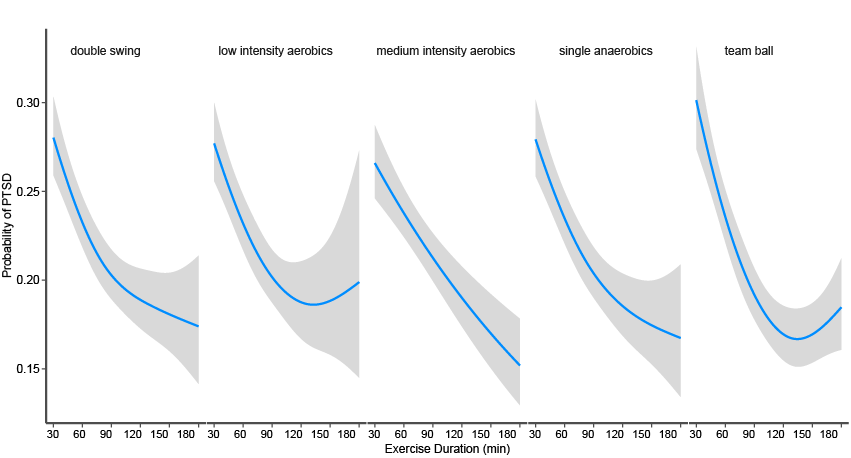


**S Fig11. PTSD as a function of exercise duration.** The lines in the figure represent smooth conditional means using generalised additive model smoothing with cubic regression splines, whereas the ribbons represent 95% confidence intervals.


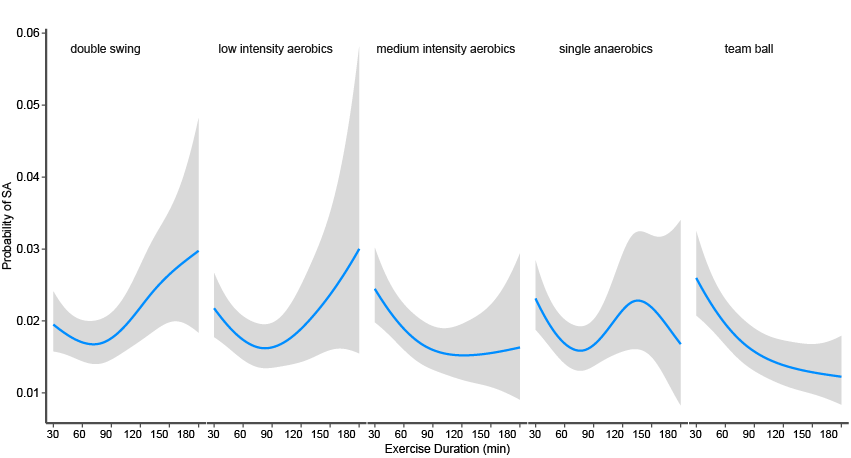


**S Fig12. SA as a function of exercise duration.** The lines in the figure represent smooth conditional means using generalised additive model smoothing with cubic regression splines, whereas the ribbons represent 95% confidence intervals.


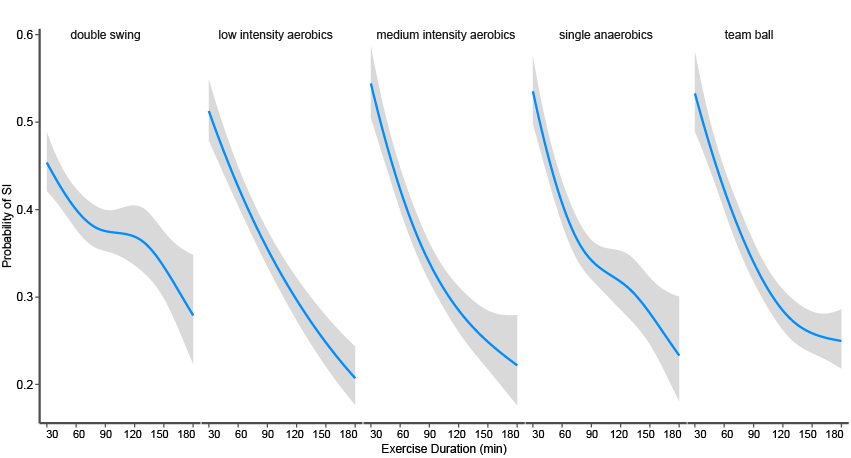


**S Fig13. SI as a function of exercise duration.** The lines in the figure represent smooth conditional means using generalised additive model smoothing with cubic regression splines whereas the ribbons represent 95% confidence intervals.

**S Fig14. Mental health outcomes as a function of exercise frequency: Female Group (N =45 432 )**

**
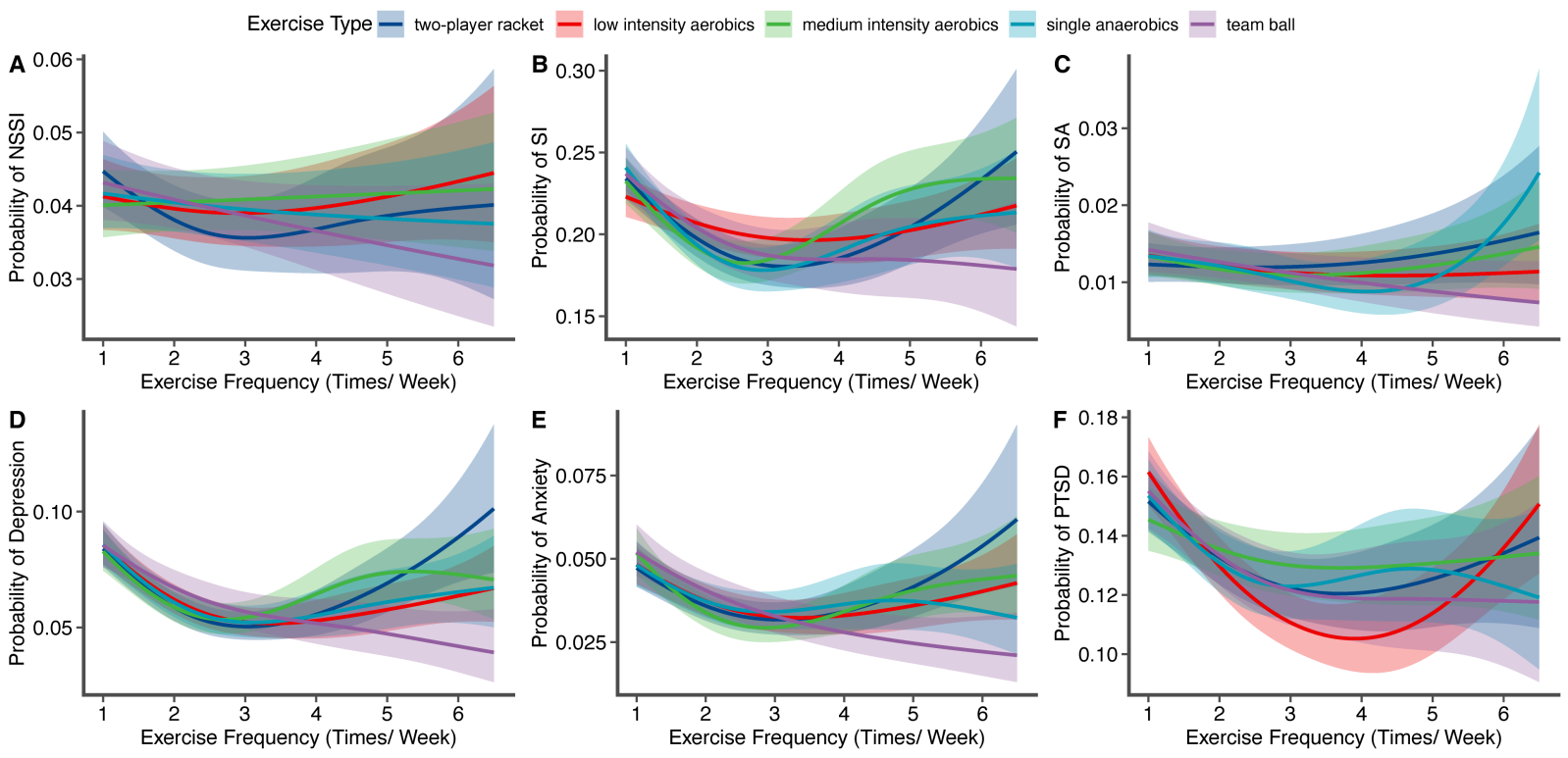
**

**S Fig15. Mental health outcomes as a function of exercise duration: Female Group (N = 45432)**


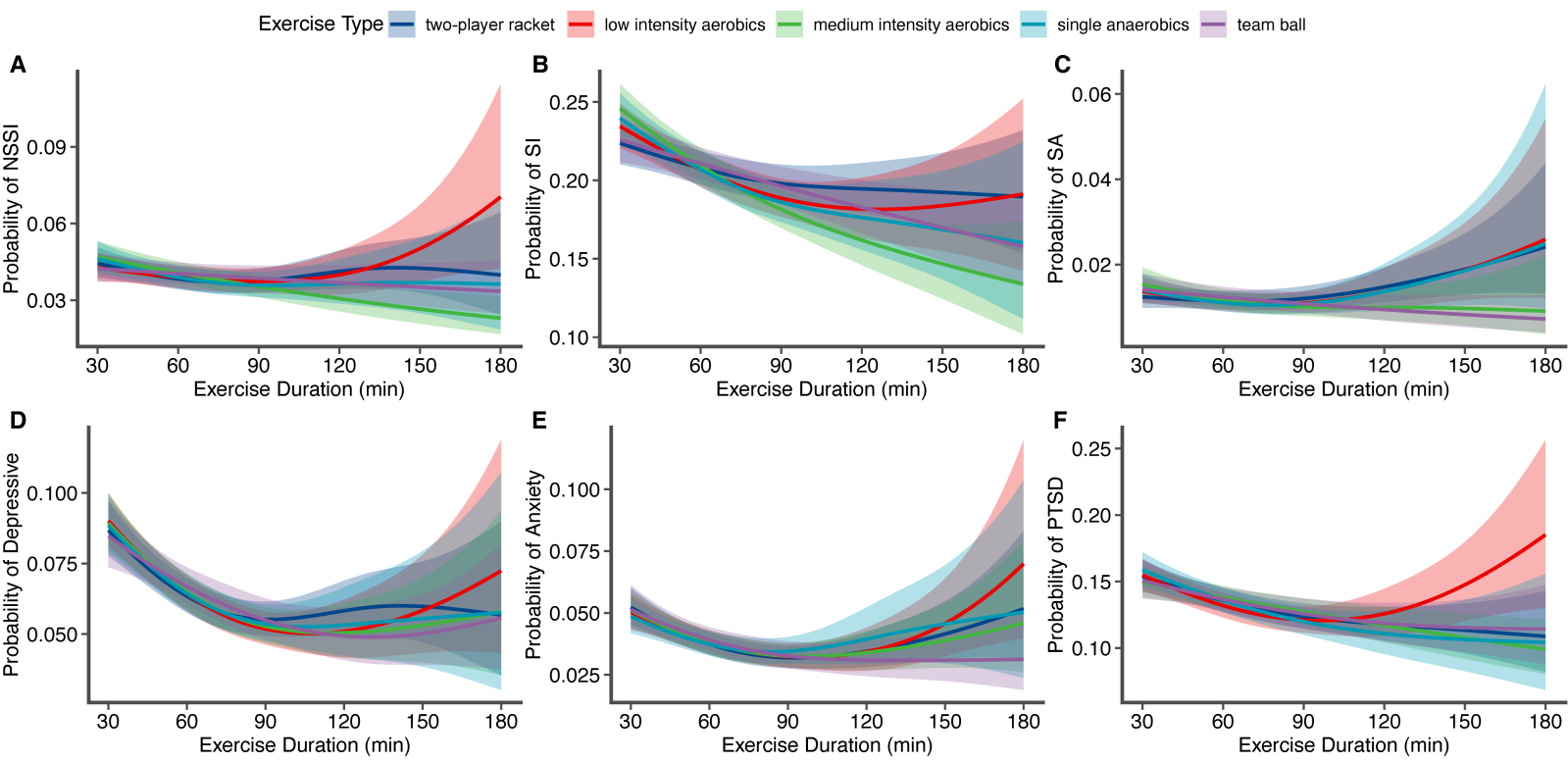


**S Fig16. Mental health outcomes as a function of exercise frequency: Male Group (N =33579 )**

**
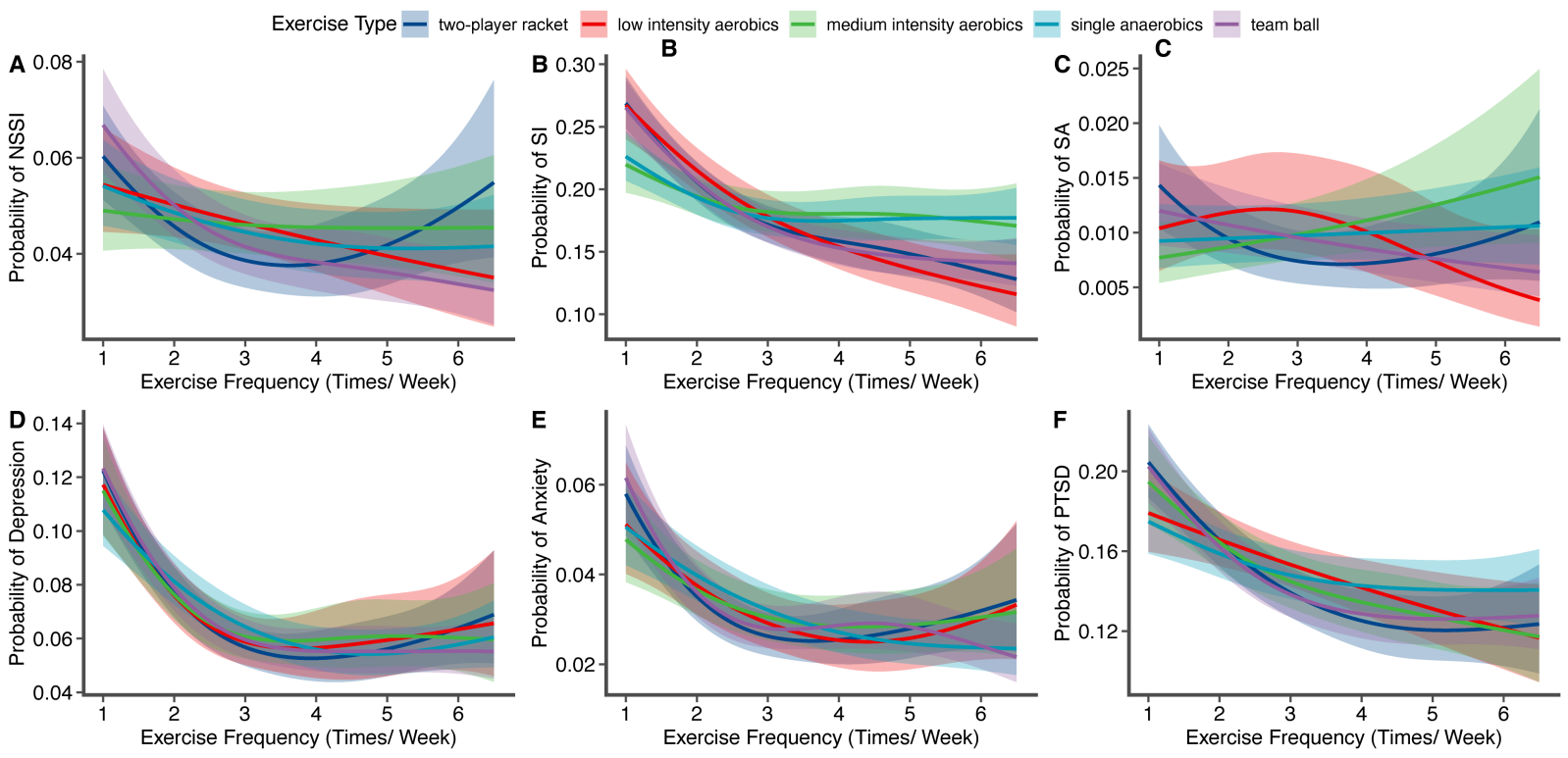
**

**S Fig17. Mental health outcomes as a function of exercise duration: Male Group (N = 33579)**


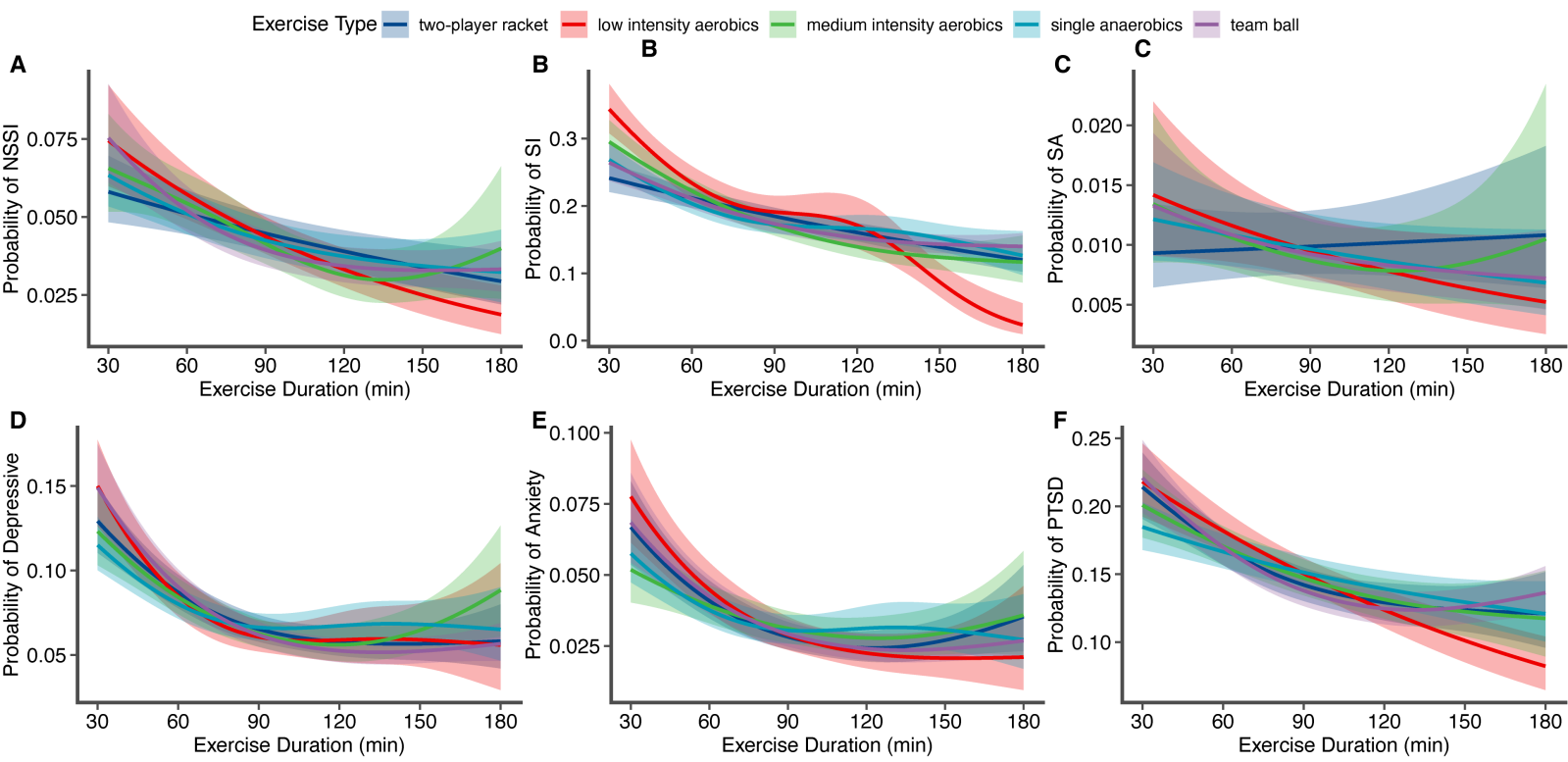

Supplement: Supplementary file 1 — Supporting Information S1 [file GPS3-39-e70031-s002.docx]
